# Lupane Triterpenes from the Leaves of *Acanthopanax* *gracilistylus*

**DOI:** 10.3390/molecules23010087

**Published:** 2018-01-01

**Authors:** Xiao-Jun Li, Qin-Peng Zou, Xiang Wang, Kwan-Woo Kim, Mao-Fang Lu, Sung-Kwon Ko, Chang-Soo Yook, Youn-Chul Kim, Xiang-Qian Liu

**Affiliations:** 1 School of Pharmacy, Hunan University of Chinese Medicine, Changsha 410208, Hunan, China; lixiaojun2017@yahoo.com (X.-J.L.); hzywtlwx@163.com (X.W.); lumf7983@126.com (M.-F.L.); 2 College of Pharmacy, Wonkwang University, Iksan 570-749, Korea; swamp1@naver.com; 3 Broad-Ocean Bio-Science and Technique Co., Ltd. of Changsha, Changsha 410205, Hunan, China; zouqinpeng@163.com; 4 Department of Oriental Medical Food & Nutrition, Semyung University, Jecheon 27136, Korea; skko@semyung.ac.kr; 5 School of Pharmacy, KyungHee University, Seoul 130-701, Korea; yookcs@khu.ac.kr

**Keywords:** *Acanthopanax gracilistylus*, Araliaceae, lupane-triterpene, acangraciligenin S, acangraciliside S

## Abstract

The phytochemical study on the leaves of *Acanthopanax gracilistylus* (Araliaceae) resulted in the discovery of a new lupane-triterpene compound, acangraciligenin S (**1**), and a new lupane-triterpene glycoside, acangraciliside S (**2**), as well as two known ones, 3α,11α-dihydroxy-lup-20(29)-en-23,28-dioic acid (**3**) and acankoreoside C (**4**). Their chemical structures were elucidated by mass, 1D- and 2D-nuclear magnetic resonance (NMR) spectroscopy. The chemical structures of the new compounds **1** and **2** were determined to be 1β,3α-dihydroxy-lup-20(29)-en-23, 28-dioic acid and 1β,3α-dihydroxy-lup-20(29)-en-23,28-dioic acid 28-*O*-[α-l-rhamnopyranosyl-(1→4)-β-d-glucopyranosyl-(1→6)-β-d-glucopyranosyl] ester, respectively. The anti-neuroinflammatory activity of the selective compounds, **1** and **3**, were evaluated with lipopolysaccharide (LPS)-induced BV2 microglia. The tested compounds showed moderate inhibitory effect of nitric oxide (NO) production.

## 1. Introduction

**Figure 1:**
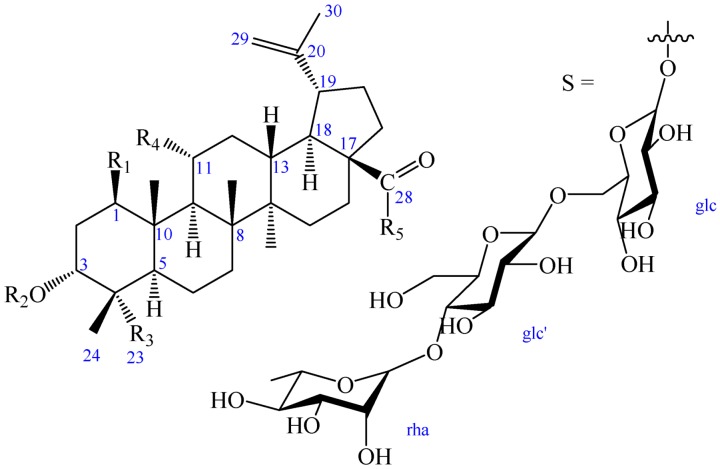
Chemical structures of compounds **1**–**4**.

*Acanthopanax gracilistylus* W. W. Smith belongs to Araliaceae, which is widely distributed in China, and its dried roots and stem barks are listed officially in the Chinese Pharmacopoeia (2015 edition) as *Acanthopanax Cortex* (named as Wujiapi), which has been used as medicine for the treatment of paralysis, arthritis, rheumatism, lameness, and liver disease [1,2,3]. Previous phytochemical investigations of this plant have led to the identification of triterpenoids [4,5,6,7], diterpenoids [8,9], monoterpenoids [10,11,12], lignans [12], steroids, cerebrosides [13], and volatile components [10,14,15,16,17,18,19], which showed diverse biological activities, such as anti-tumor [20,21], anti-inflammatory [22,23,24], and liver protective effects [3]. As part of our ongoing research to identify bioactive substances from the genus *Acanthopanax*, we investigated a MeOH extract of the leaves of *A. gracilistylus* and identified two new lupane triterpenoids (**1** and **2**), and two known compounds (**3** and **4**) by using high-speed countercurrent chromatography (HSCCC) in conjunction with a high-performance liquid chromatography (HPLC) isolation system (Figure 1). In this paper, the isolation and structural elucidation of the new isolates, as well as an evaluation of their bio-activities, are reported.

## 2. Results and Discussion

**Figure 2:**
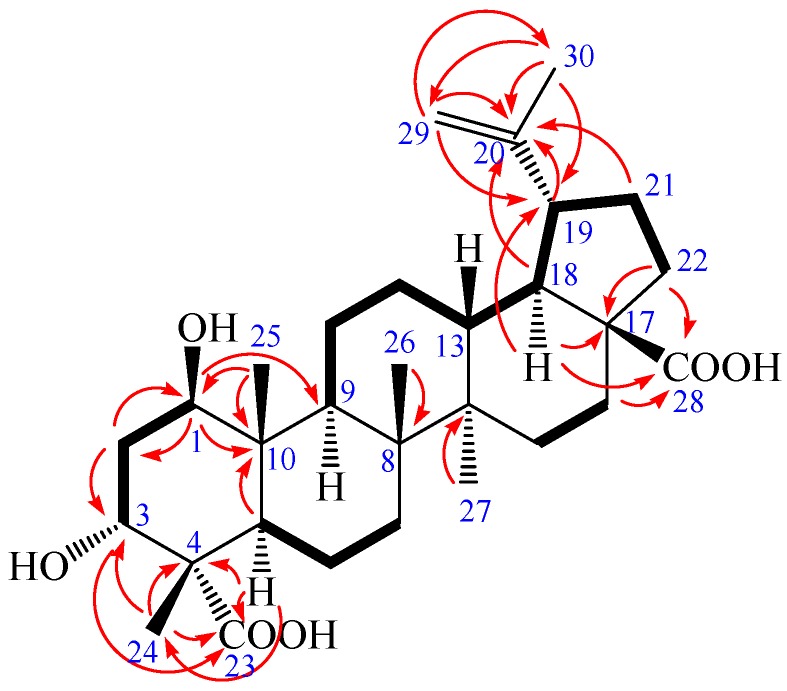
^1^H-^1^H COSY (bold) and selected HMBC (arrow) correlations of **1**.

**Figure 3:**
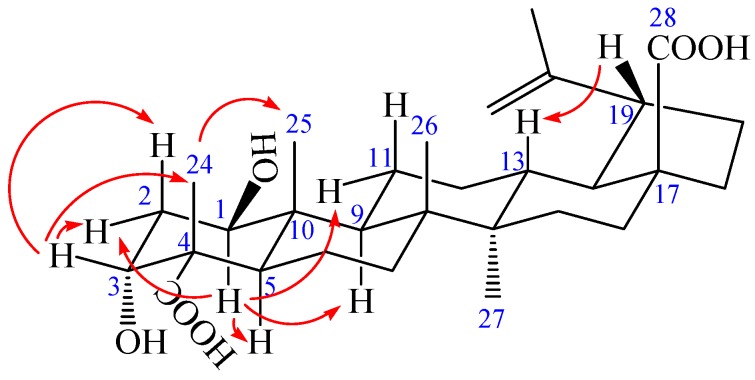
The key NOESY (arrow) correlations of **1**.

Compound **1** was obtained as a colorless needle, m.p. 282.6 °C, and gave a positive reaction in the Liebermann–Burchard test. Its molecular formula of C_30_H_46_O_6_ was determined on the basis of ESI-MS at *m*/*z* = 501.3 [M − H]^−^ (negative) and HR-ESI-MS at *m*/*z* = 501.3221 [M − H]^−^ (calcd. for C_30_H_45_O_6_: 501.3222), indicating eight degrees of unsaturation. The ^1^H-NMR spectrum of **1** (in acetone-*d*_6_) showed the following signals: five tertiary methyl groups at δ 0.97, 0.99, 1.07, 1.17 and 1.71 (each 3H, s); two olefinic protons at δ 4.58 (1H, m) and 4.72 (1H, d, *J* = 1.68 Hz); two hydroxy-substituted methines at δ 3.82 (1H, t, *J* = 2.40 Hz) and 3.84 (1H, dd, *J* = 8.16, 3.96 Hz). The ^13^C-NMR and distortionless enhancement by polarization transfer (DEPT) spectra revealed 30 carbon signals. The skeleton of **1** was recognized to be lupane triterpenoid by ^1^H- and ^13^C-NMR analysis (see Table 1), with the typical olefinic carbons at δ 151.89 (C-20) and 110.26 (C-29), five quaternary methyl carbons at δ 13.24, 15.40, 17.31, 17.92, and 19.72, two oxymethine carbons at δ 73.99 and 75.46, and two carboxyl signals at δ 177.84 and 178.16, respectively. The assignment of the α-hydroxyl group at C-3 was performed by comparing its spectral data with literature values [25,26,27,28]. The chemical shifts of C-3 (δ 73.99) and C-4 (δ 52.27) further confirmed the axial configuration of the 3-hydroxyl group by comparing with the corresponding data of the 3β-hydroxy-lup-20(29)-en-23,28-dioic acid [δ values for 84.4 (C-3) and 43.0 (C-4)] [29]. Additionally, the carbon chemical shifts of C-23 (δ 178.16) and C-28 (δ 177.84) were also similar to those of 3β-hydroxy-lup-20(29)-en-23,28-dioic acid [δ values for 178.7 (C-23) and 177.3 (C-28)] suggesting that the two carboxyl groups were at C-23 and C-28 [29]. In heteronuclear multiple bond connectivity (HMBC) spectrum, the H-1 proton signal at δ 3.84 (1H, dd, *J* = 8.16, 3.96 Hz) correlated with carbons C-2 (δ 37.36), C-9 (δ 52.98), C-10 (δ 44.33), and C-25 (δ 13.24); protons H-2 at δ 1.82 (1H, m) and H-25 at δ 0.97 (3H, s) correlated with carbon C-1 (δ 75.46); the H-3 proton signal at δ 3.82 (1H, t, *J* = 2.40 Hz) correlated with carbons C-5 (δ 45.25) and C-23 (δ 178.16); protons H-2 at δ 1.82 (1H, m) and H-24 at δ 1.17 (3H, s) correlated with carbon C-3 (δ 73.99); protons H-5 at δ 1.95 (1H, m) and H-24 at δ 1.17 (3H, s) correlated with carbon C-23 (δ 178.16); protons H-16 at δ 1.48 (1H, m), H-18 at δ 1.64 (1H, m), and H-22 at δ 1.92 (1H, m) correlated with carbon C-28 (δ 177.84); the correlations from H-29 at δ 4.58 (1H, m, Ha) and δ 4.72 (1H, d, *J* = 1.68 Hz, Hb) to C-19 (δ 48.24), C-20 (δ 151.89), and C-30 (δ 19.72); the correlations from H-30 at δ 1.71 (3H, s) to C-19 (δ 48.24), C-20 (δ 151.89), and C-29 (δ 110.26). These pieces of evidence confirmed that the double bond was at C-20/C-29 and the other hydroxyl group was at C-1 (Figure 2 and Table S1). The nuclear Overhauser enhancement spectroscopy (NOESY) spectrum of **1** showed the correlations between the proton of H-1 and H-2*_eq_*, H-1 and H-5, H-1, and H-9, H-1 and H-11*_eq_*, suggesting that H-1 was axial in orientation, which, in turn, suggested that the hydroxyl group at C-1 was β-positioned. Similarly, cross-peaks between H-24 and H-25, as well as H-3, H-3 and H-2*_eq_* as well as H-2*_ax_*, which indicated that the methyl group (H-24) was axial and H-3 was equatorial, which, in turn, suggested that the hydroxyl group at C-3 and carboxyl group at C-4 were α-positioned. Furthermore, cross-peaks between H-19 and H-13 confirmed that the isopropenyl group at C-19 was α-positioned (Figure 3). Based on the above spectral data, the structure of **1** was determined as 1β,3α-dihydroxy-lup-20(29)-en-23,28-dioic acid, a new compound named acangraciligenin S.

**Figure 4:**
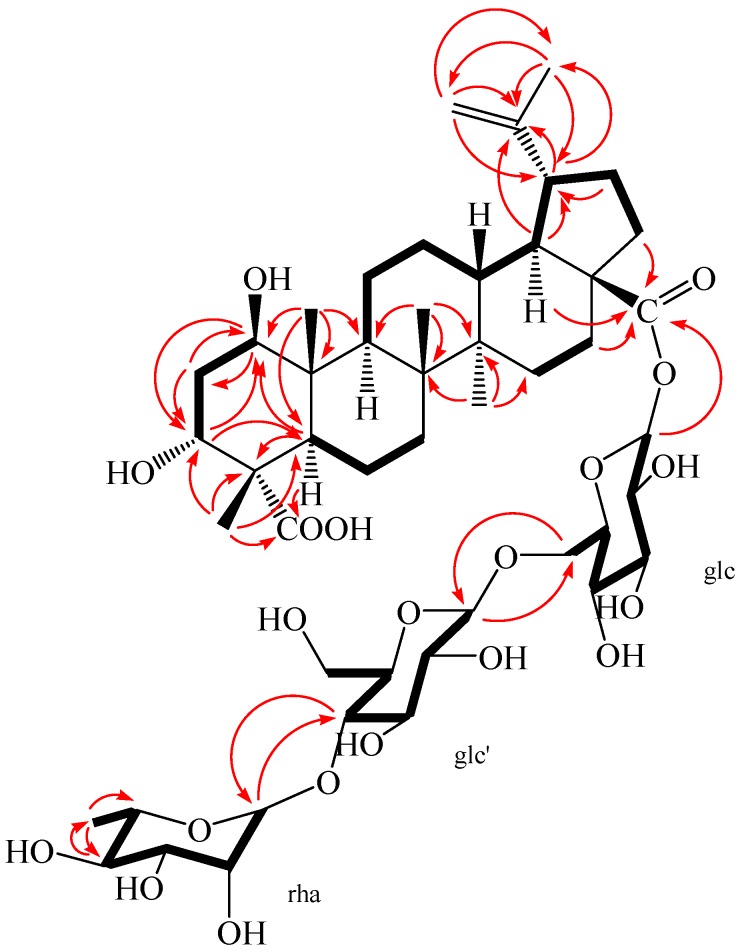
^1^H-^1^H COSY (bold) and selected HMBC (arrow) correlations of **2**.

Compound **2** was obtained as a white amorphous powder, m.p. 230.5 °C, and gave positive responses in Liebermann–Burchard and Molish tests. Its molecular formula of C_48_H_76_O_20_ was determined on the basis of ESI-MS at *m*/*z* = 972.5 [M]^+^ and HR-ESI-MS at *m*/*z* = 971.4865 [M − H]^−^ (negative) (calcd. for C_48_H_75_O_20_: 971.4857), indicating eleven degrees of unsaturation. The ^1^H-NMR spectrum of **2** (in methanol-*d*_4_) showed the following signals: five tertiary methyl groups at δ 0.95, 0.98, 1.03, 1.09 and 1.70 (each 3H, s); one secondary methyl group at δ 1.25 (3H, d, *J* = 4.96 Hz), assigned to H-6″ of the rhamnose; and two olefinic protons at δ 4.58 (1H, brs) and 4.72 (1H, brs); two hydroxy-substituted methines at δ 3.67 (1H, m) and 3.80 (1H, m). In addition, three anomeric protons were at δ 4.37 (1H, d, *J* = 6.28 Hz), 4.84 (1H, overlapped), and 5.45 (1H, d, *J* = 6.56 Hz), suggesting the appearance of three sugar units. The ^13^C-NMR and DEPT spectra revealed 48 carbon signals, of which, 30 signals were assigned to a triterpenoid sapogenol moiety and 18 signals belong to three monosaccharide moieties. The aglycone of **2** was recognized to be lupane triterpene type by ^1^H- and ^13^C-NMR analysis (see Table 1), with the typical olefinic carbons at δ 151.77 (C-20) and 110.41 (C-29), five quaternary methyl carbons at δ 13.17, 15.10, 17.14, 18.08, and 19.49, two oxymethine carbons at δ 74.14 and 76.33, and two carboxyl signals at δ 176.40 and 182.60, respectively. Compared with the ^1^H- and ^13^C-NMR data of the aglycone of **2** and compound **1**, the high similarity indicated that the aglycone of **2** was the same as that of compound **1**. Assignments of the β-hydroxyl group at C-1 (δ 76.33), α-hydroxyl group at C-3 (δ 74.14), and two carboxyl groups at C-23 (δ 182.60) and C-28 (δ 176.40) were performed by comparing its spectral data with compound **1** [δ values for 75.46 (C-1), 73.99 (C-3), 178.16 (C-23) and 177.84 (C-28)] and literature values [28]. In HMBC spectrum, the H-1 proton signal at δ 3.80 (1H, m) correlated with carbons C-2 (δ 36.79), C-3 (δ 74.14), C-9 (δ 53.06), C-10 (δ 44.47), and C-25 (δ 13.17); protons H-2 at δ 1.72 (1H, m), H-3 at δ 3.67 (1H, m), H-5 at δ 1.87 (1H, m), and H-25 at δ 0.95 (3H, s) correlated with carbon C-1 (δ 76.33); the H-3 proton signal at δ 3.67 (1H, m) correlated with carbons C-1 (δ 76.33) and C-5 (δ 46.11); protons H-2 at δ 1.80 (1H, m) and H-24 at δ 1.09 (3H, s) correlated with carbon C-3 (δ 74.14); protons H-5 at δ 1.87 (1H, m) and H-24 at δ 1.09 (3H, s) correlated with carbon C-23 (δ 182.60); protons H-16 at δ 1.44 (1H, m), H-18 at δ 1.65 (1H, m), and H-22 at δ 1.94 (1H, m) correlated with carbon C-28 (δ 176.40); the correlations from H-29 at δ 4.58 (1H, brs, Ha) and δ 4.72 (1H, brs, Hb) to C-19 (δ 48.36), C-20 (δ 151.77), and C-30 (δ 19.49); the correlations from H-30 at δ 1.70 (3H, s) to C-19 (δ 48.36), C-20 (δ 151.77), and C-29 (δ 110.41). These pieces of evidences further confirmed that the double bond was at C-20/C-29 and the two hydroxyl groups were at C-1 and C-3, respectively (Figure 4 and Table S2).

**Figure 5:**
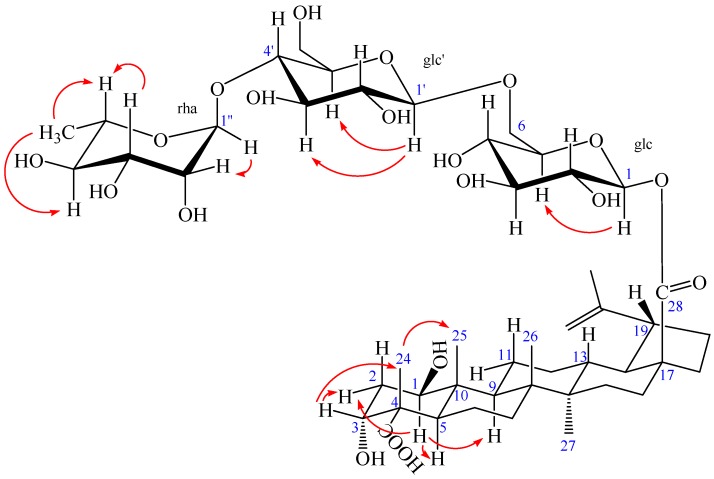
The key NOESY (arrow) correlations of **2**.

The NOESY spectrum of **2** showed the correlations between the proton of H-1 and H-2*_eq_*, H-1 and H-5, H-1, and H-9, suggesting that H-1 had an *axial* orientation, which, in turn, suggested that the hydroxyl group at C-1 was β-positioned. Similarly, cross-peaks between H-24 and H-25, as well as H-3, H-3 and H-2*_eq_*, indicated that the methyl group (H-24) was axial and H-3 was equatorial, which, in turn, suggested that the hydroxyl group at C-3 and carboxyl group at C-4 were α-positioned (Figure 5). Alkaline hydrolysis of **2** with 5% KOH in MeOH gave a sapogenol (**2a**), a colorless needle, mp 282.5 °C, together with a mixture of sugars. The sugar mixture was identified to be composed of D-glucose and L-rhamnose by thin-layer chromatography (TLC) with their authentic sample. The ^1^H and ^13^C NMR data of **2a** (see Experimental Section) were the same as those of **1**, which further confirmed that this aglycone (**2a**) was 1β,3α-dihydroxy-lup-20(29)-en-23,28-dioic acid. Moreover, the HMBC correlations between the inner glc H-1 (δ 5.45) and C-28 of the aglycone (δ 176.40), between outer glc H-1′ (δ 4.37) and inner glc C-6 (δ 69.55), between rha H-1″ (δ 4.84) and glc C-4′ (δ 79.51) were observed (Figure 4). In addition, the NOESY correlations between the inner glc H-1 and glc H-5, between outer glc H-1′ and glc H-3′ as well as glc H-5′, between rha H-1″ and rha H-2″, rha H-5″ and rha H-3″, as well as rha H-6″, rha H-4″ and rha H-6″, which further confirmed the inner glucose, outer glucose, and rhamnose were *β*-D, *β*-D, and *α*-L positioned, respectively (Figure 5). These results suggest the sequence of sugar linkages of **2**. The carbon signals of the sugar moiety were superimposable on those of characteristic triterpene glycosides isolated from *Acanthopanax* species [4,5,6,7,25,26,27,28]. Consequently, the structure of **2** was determined as 1*β*,3*α*-dihydroxy-lup-20(29)-en-23,28-dioic acid 28-*O*-[*α*-l-rhamnopyranosyl-(1→4)-*β*-d-glucopyranosyl-(1→6)-*β*-d-glucopyranosyl] ester, a new compound named acangraciliside S.

Compounds **3** and **4** were identified as 3α,11α-dihydroxy-lup-20(29)-en-23,28-dioic acid [30] and acankoreoside C [31], respectively, by comparing their NMR and mass spectral data with the literature values. (The ^1^H- and ^13^C-NMR data of **3** and **4** see Supplementary Materials: Table S3)

Moreover, the cytotoxicity and inhibition of production of nitric oxide (NO) of selected isolates (**1** and **3**) from *A. gracilistylus* were investigated on LPS-stimulated BV2 microglias. Compounds **2** and **4** were excluded from biological evaluation because of sample shortage. As shown in Table 2, the production of NO was down-regulated moderately when their concentration was 80 μM with the inhibition as 38.6% and 40.4%, respectively.

## 3. Experimental Section

### 3.1. General Experimental Procedures

Melting points (uncorrected) were measured using a Boetius micromelting point apparatus (Beijing Jingjing Science and Technology Co., Ltd., Beijing, China). ^1^H-NMR (400 MHz), ^13^C-NMR (100 MHz) and 2D-NMR were recorded at room temperature in CD_3_COCD_3_ or CD_3_OD using Bruker DRX-400 NMR spectrometer (Bruker Biospin, Zurich, Switzerland) and chemical shifts were presented in δ (ppm) values relative to tetramethylsilane (TMS) as internal standard. HR-ESI-MS was performed on an API QSTAR spectrometer (Agilent Technologies, Santa Clara, CA, USA). The high-speed countercurrent chromatography (HSCCC) instrument was performed in the present study using a model an TBE-300A HSCCC (Shanghai Tauto Biotech Co., Ltd., Shanghai, China). Semi-preparative HPLC was performed on an LC-20A liquid chromatograph (Shimadzu Technologies, Kyoto, Japan). Column chromatography was carried out on silica gel (200–300 mesh and 100–200 mesh, Qingdao Marine Chemical Co., Ltd., Qingdao, China), and Sephadex LH-20 (Merck, Darmstadt, Germany). RP-TLC was measured on a precoated RP-18F_254s_ (Merck, Darmstadt, Germany) plates. TLC was conducted on self-made silica gel G (Qingdao Marine Chemical Industry, Qingdao, China) plates and spots were visualized by spraying with 10% H_2_SO_4_ in ethanol (*v*/*v*) followed by heating at 105 °C. The standard d-glucose and l-rhamnose were purchased from Beijing North Carolina Souren Biotechnology Research Institute (Beijing, China).

### 3.2. Collection and Identification of Biological Materials

The leaves of *A. gracilistylus* were collected from its natural habitat in Yuanling, Hunan Province, China, in July 2015 and identified by X.Q. Liu, the corresponding author of this work. A voucher specimen has been deposited in the Herbarium of Hunan University of Chinese Medicine, Hunan, China (no. 201507).

### 3.3. Extraction and Isolation

The dried, powdered leaves of *A. gracilistylus* (600 g) were extracted with methanol under reflux three times (3 × 3 L). The solvent was then removed under reduced pressure to yield a residue (120 g), which was suspended in distilled water and successively partitioned with petroleum ether (PE, 60–90), EtOAc, and *n*-BuOH, respectively. The EtOAc extract (28 g) was subjected to column chromatography (CC) on silica gel eluting with a gradient of CHCl_3_-MeOH (35:1 to 10:1, *v*/*v*) to obtain the discolored fraction (E1). E1 was partitioned by HSCCC using two-phase solvent system composing of n-hexane-ethyl acetate-methanol-water (1:2:1.6:1, *v*/*v*/*v*/*v*) with the flow rate of mobile phase of 2.0 mL/min, rotation rate of 800 r.p.m, to generate five fractions (F1–F5). Compounds **1** (5.0 mg) and **3** (6.0 mg) were yielded from F2 (25 mg) by using Pre-HPLC with CH_3_CN-H_2_O (40:60, *v*/*v*) for further purification. Similarly, the *n*-BuOH extract (25 g) was partitioned by HSCCC using two-phase solvent system composing of ethyl acetate-butanol-methanol-water (3:0.3:0.8:4, *v*/*v*/*v*/*v*) with the flow rate of mobile phase of 2.0 mL/min, rotation rate of 900 r.p.m, to obtain five fractions (F6–F10). F6 (35 mg) was further chromatographed on Pre-HPLC with CH_3_CN-H_2_O (25:75, *v*/*v*) to produce two compounds **2** (11.3 mg) and **4** (5.3 mg).

Acangraciligenin S (**1**) was obtained as a colorless needle: mp. 282.6 °C; ^1^H- and ^13^C-NMR data: (Table 1); (−)-HR-ESI-MS *m*/*z* 501.3221 [M − H]^−^ (calcd. for C_30_H_45_O_6_: 501.3222).

Acangraciliside S (**2**) was obtained as a white amorphous powder: mp. 230.5 °C; ^1^H- and ^13^C-NMR data: (Table 1); (−)-HR-ESI-MS *m*/*z* 971.4865 [M − H]^−^ (calcd. for C_48_H_75_O_20_: 971.4857).

### 3.4. Alkaline Hydrolysis of **2**

Compound **2** (10 mg) was hydrolyzed with 8 mL of 5% KOH in MeOH for 2 h at 80 °C. The reaction mixture was neutralized with 2 M HCl in H_2_O and extracted with EtOAc. The aqueous layer was filtered, concentrated and chromatographed on TLC plate in which d-glucose and l-rhamnose were detected by comparing with standard samples [4]. The EtOAc layer was evaporated *in vacuo* and the residue was performed on a preparative HPLC column by using elution with CH_3_CN-H_2_O (40:60, *v*/*v*) to obtain sapogenol fraction **2a** (2 mg).

Compound **2a**: Colorless needles, m.p. 282.5 °C. ^1^H-NMR (400 MHz, CD_3_COCD_3_) δ: 3.84 (1H, dd, *J* = 8.16, 3.96 Hz, H-1), 3.82 (1H, t, *J* = 2.40 Hz, H-3), 1.95 (1H, m, H-5), 1.72 (1H, m, H-9), 2.34 (1H, td, *J* = 10.32, 3.0 Hz, H-13), 1.64 (1H, m, H-18), 3.05 (1H, td, *J* = 8.76, 4.0 Hz, H-19), 1.17 (3H, s, H-24), 0.97 (3H, s, H-25), 0.99 (3H, s, H-26), 1.08 (3H, s, H-27), 4.59 (1H, m, H-29a), 4.73 (1H, d, *J* = 1.68 Hz, H-29b), 1.71 (3H, s, H-30); ^13^C-NMR (100 MHz, CD_3_COCD_3_) δ: 75.45 (C-1), 37.35 (C-2), 73.98 (C-3), 52.26 (C-4), 45.23 (C-5), 22.08 (C-6), 35.18 (C-7), 42.75 (C-8), 52.97 (C-9), 44.32 (C-10), 24.66 (C-11), 26.85 (C-12), 39.13 (C-13), 43.71 (C-14), 30.73 (C-15), 33.14 (C-16), 56.96 (C-17), 50.29 (C-18), 48.22 (C-19), 151.88 (C-20), 31.59 (C-21), 37.82 (C-22), 178.14 (C-23), 17.90 (C-24), 13.23 (C-25), 17.29 (C-26), 15.38 (C-27), 177.82 (C-28), 110.25 (C-29), 19.70 (C-30).

### 3.5. MTT Assay for Cell Viability

BV2 microglias were maintained at 5 × 10^5^ cells/mL in Dulbecco minimum essential medium (DMEM) medium supplemented with 10% heat-inactivated fetal bovine serum (FBS), penicillin G (100 U/mL), streptomycin (100 mg/L), and L-glutamine (2 mM) and incubated at 37 °C in a humidified atmosphere containing 5% CO_2_. Cell viability was determined by adding 100 mg/mL of 3-[4,5-dimethylthiazol-2-yl]-2,5-diphenyltetrazolium bromide (MTT) to 1 mL of a cell suspension (1 × 10^5^ cells/mL in 96-well plates) and incubated for 30 min. The formazan formed was dissolved in acidic 2-propanol, and the optical density was measured at 540 nm.

### 3.6. Nitrite Assay

The concentration of nitric oxide (NO) in the conditioned media was determined by a method based on the Griess reaction [32]. An aliquot of each supernatant (100 mL) was mixed with the same volume of Griess reagent (0.1% (*w*/*v*) *N*-(1-naphathyl)-ethylenediamine and 1% (*w*/*v*) sulfanilamide in 5% (*v*/*v*) phosphoric acid) for 10 min at room temperature. The absorbance of the final product was measured spectrophotometrically at 540 nm using an enzyme-linked immunosorbent assay (ELISA) plate reader. The nitrite concentration in the samples was determined from a standard curve of sodium nitrite prepared in phenol red-free DMEM.

### 3.7. Statistical Analysis

All values are expressed as the mean ± S.D. Differences between mean values of normally-distributed data were assessed with one-way analysis of variance (ANOVA) (Newman Keuls *t*-test). Statistical analysis was performed using GraphPad Prism software, version 3.03 (GraphPad Software Inc., San Diego, CA, USA). Statistical significance was accepted at *p* < 0.05.

## 4. Conclusions

In summary, in this work, a phytochemical study on the leaves of *A. gracilistylus* (Araliaceae) by using HSCCC-HPLC combinatorial chromatography method resulted in the discovery of four lupane-triterpenoids, including two new compounds, acangraciligenin S (**1**) and acangraciliside S (**2**), as well as two known species, 3α,11α-dihydroxy-lup-20(29)-en-23,28-dioic acid (**3**) and acankoreoside C (**4**). Two selected compounds were also evaluated for their inhibitory activity against lipopolysaccharide (LPS)-induced nitric oxide (NO) production in murine microglia BV2 cells. Compounds **1** and **3** showed moderate abilities to inhibit NO production and had no influence on cell viability, as determined by the MTT method.

## Figures and Tables

**Table:** 

	**R_1_**	**R_2_**	**R_3_**	**R_4_**	**R_5_**
**1**	OH	OH	COOH	H	OH
**2**	OH	OH	COOH	H	S
**2a**	OH	OH	COOH	H	OH
**3**	H	OH	COOH	OH	OH
**4**	H	-β-d-glc	CH_3_	OH	S

**Table 1:** NMR spectral data of compounds **1**–**2**.

Position	1 δ_C_^a,c^	δ_H_^a,d^ [mult. (*J* in Hz)]	2 δ_C_^b,c^	δ_H_^b,d^ [mult. (*J* in Hz)]
**Aglycone**				
1	75.46	3.84 (1H, dd, 8.16, 3.96)	76.33	3.80 (1H, m)
2	37.36	1.82 (1H, m); 1.87 (1H, m)	36.79	1.72 (1H, m); 1.80 (1H, m)
3	73.99	3.82 (1H, t, 2.40)	74.14	3.67 (1H, m)
4	52.27	-	52.24	-
5	45.25	1.95 (1H, m)	46.11	1.87 (1H, m)
6	22.09	1.35 (1H, m); 1.57 (1H, m)	22.17	1.26 (1H, m); 1.58 (1H, m)
7	35.19	1.31 (1H, m); 1.56 (1H, m)	35.09	1.30 (1H, m); 1.55 (1H, m)
8	42.76	-	42.96	-
9	52.98	1.72 (1H, dd, 10.08, 2.68)	53.06	1.72 (1H, m)
10	44.33	-	44.47	-
11	24.68	1.32 (1H, m); 2.43 (1H, brd, 9.80)	24.82	1.36 (1H, m); 2.28 (1H, m)
12	26.87	1.12 (1H, dd, 10.52, 3.76); 1.68 (1H, m)	26.94	1.12 (1H, m); 1.68 (1H, m)
13	39.14	2.34 (1H, td, 10.32, 3.0)	39.13	2.27 (1H, m)
14	43.72	-	43.82	-
15	30.75	1.20 (1H, m); 1.46 (1H, m)	30.86	1.15 (1H, m); 1.54 (1H, m)
16	33.15	1.48 (1H, m); 2.25 (1H, dt, 9.84, 2.8)	32.95	1.44 (1H, m); 2.33 (1H, m)
17	56.98	-	57.93	-
18	50.30	1.64 (1H, m)	50.60	1.65 (1H, m)
19	48.24	3.05 (1H, td, 8.76, 4.0)	48.36	3.00 (1H, m)
20	151.89	-	151.77	-
21	31.61	1.34 (1H, m); 1.90 (1H, m)	31.55	1.37 (1H, m); 1.94 (1H, m)
22	37.84	1.48 (1H, m); 1.92 (1H, m)	37.68	1.48 (1H, m); 1.94 (1H, m)
23	178.16	-	182.6	-
24	17.92	1.17 (3H, s)	18.08	1.09 (3H, s)
25	13.24	0.97 (3H, s)	13.17	0.95 (3H, s)
26	17.31	0.99 (3H, s)	17.14	0.98 (3H, s)
27	15.40	1.07 (3H, s)	15.10	1.03 (3H, s)
28	177.84	-	176.40	-
29	110.26	4.58 (1H, m); 4.72 (1H, d, 1.68)	110.41	4.58 (1H, brs); 4.72 (1H, brs)
30	19.72	1.71 (3H, s)	19.49	1.70 (3H, s)
**C-28 O-glc**				
1			95.26	5.45 (1H, d, 6.56)
2			74.00	3.33 (1H, m)
3			78.28	3.42 (1H, m)
4			70.95	3.43 (1H, m)
5			78.06	3.54 (1H, m)
6			69.55	3.81 (1H, m); 4.11 (1H, dd, 9.48, 1.36)
**glc′(1→6)glc**				
1′			104.56	4.37 (1H, d, 6.28)
2′			75.32	3.23 (1H, m)
3′			76.71	3.45 (1H, m)
4′			79.51	3.53 (1H, m)
5′			76.89	3.30 (1H, m)
6′			61.90	3.65 (1H, m); 3.80 (1H, m)
**rha(1→4)glc′**				
1″			102.92	4.84 (1H, overlapped)
2″			72.44	3.81 (1H, m)
3″			72.16	3.62 (1H, m)
4″			73.75	3.38 (1H, m)
5″			70.64	3.95 (1H, m)
6″			17.84	1.25 (3H, d, 4.96)

Note: Assignments were performed by HMQC, HMBC, ^1^H-^1^H COSY, and NOESY experiments; Glc: d-glucopyranosyl; Rha: l-rhamnopyranosyl; ^a^ Measured in CD_3_COCD_3_; ^b^ Measured in CD_3_OD; ^c^ 100 MHz; ^d^ 400 MHz.

**Table 2:** Inhibitory effects of compounds **1** and **3** against LPS-Induced NO production in murine microglia BV2 cells ^a^.

Compounds	Inhibition	Cell Viability
**1**	38.6% in 80 μM	86.44%
**3**	40.4% in 80 μM	83.90%

^a^ Data is presented as the mean of three experiments ± SD.

## Supplementary Materials

The following ^1^H-NMR, ^13^C-NMR, 2D-NMR, ESI-MS, and HR-ESI-MS spectra are available as supporting data. Supplementary materials are available online.

## References

[1] Chinese Pharmacopoeia Commission (2015). Chinese Pharmacopoeia of the People’s Republic of China.

[2] Ni N., Liu X.Q. (2006). Advances in studies on plants of *Acanthopanax* Miq. in Araliaceae. Chin. Tradit. Herb. Drugs.

[3] Zhang B.X., Li N., Zhang Z.P., Liu H.B., Zhou R.R., Zhong B.Y., Zou M.X., Dai X.H., Xiao M.F., Liu X.Q. (2011). Protective effect of *Acanthopanax gracilistylus*-extracted Acankoreanogenin A on mice with fulminant hepatitis. Int. Immunopharmacol..

[4] Yook C.S., Liu X.Q., Chang S.Y., Park S.Y., Nohara T. (2002). Lupane-triterpene glycosides from the leaves of *Acanthopanax gracilistylus*. Chem. Pharm. Bull..

[5] Liu X.Q., Chang S.Y., Park S.Y., Nohara T., Yook C.S. (2002). A new lupane-triterpene glycoside from the leaves of *Acanthopanax gracilistylus*. Arch. Pharm. Res..

[6] Liu X.Q., Chang S.Y., Yook C.S. (2006). Lupane-triterpenoids from the leaves of *Acanthopanax gracilistylus*. J. Lanzhou Univ. Nat. Sci..

[7] Zou Q.P., Liu X.Q., Lee H.K., Oh O.J. (2011). Lupane-triterpenoids from the methanol extracts of leaves of *Acanthopanax gracilistylus* W. W. Smith. J. Lanzhou Univ. Nat. Sci..

[8] Tang X.Y., Ma Y.C., Li P.J. (1995). Separation and identification of the anti-inflammatory diterpene from the root cortices of *Acanthopanax gracilistylus* W. W. Smith. Chin. J. Chin. Mater. Med..

[9] Wu Z.Y., Zhang Y.B., Zhu K.K., Luo C., Zhang J.X., Cheng C.R., Feng R.H., Yang W.Z., Zeng F., Wang Y. (2014). Anti-inflammatory diterpenoids from the root bark of *Acanthopanax gracilistylus*. J. Nat. Prod..

[10] Liu X.Q., Zhang C.Y., Yin W.J., Liu Z.X., Yook C.S. (2001). Analysis of volatile oil components of *Acanthopanax gracilistylus*. Chin. Tradit. Herb. Drugs.

[11] Liu X.Q., Park S.Y., Yook C.S. (2002). Studies on the constituents of the stem barks of *Acanthopanax gracilistylus* W. W. Smith. Nat. Prod. Sci..

[12] Liu X.Q., Yook C.S., Chang S.Y. (2004). Chemical constituents of *Acanthopanax gracilistylus*. Chin. Tradit. Herb. Drugs.

[13] Zhang J.Y., Pu S.B., Qian S.H., Liu D. (2011). New cerebrosides from *Acanthopanax gracilistylus*. Chin. J. Nat. Med..

[14] Liu X.Q., Chang S.Y., Ro S.H., Yook C.S. (2002). Constituents of *Acanthopanax gracilistylus* W. W. Smith. Nat. Med..

[15] An S.Y., Qian S.H., Jiang J.Q., Wang K.C. (2009). Chemical constituents in leaves of *Acanthopanax gracilistylus*. Chin. Tradit. Herb. Drugs.

[16] Xian L.N., Qian S.H., Li Z.L. (2010). Studies on the chemical constituents from the stems of *Acanthopanax gracilistylus*. J. Chin. Med. Mater..

[17] Zhang J.Y., Pu S.B., Qian S.H., Liu D., Wang K.C. (2011). Studies on the chemical constituents in fruits of *Acanthopanax gracilistylus*. J. Chin. Med. Mater..

[18] Song X.H., Xu G.J., Jin R.L. (1987). Studies on chemical constituents of the root bark of *Acanthopanax gracilistylus*. J. Chin. Pharm. Univ..

[19] Dai L., Liu X.Q., Xie X., Liu H.Y. (2014). Characterization of stereostructure by X-ray and technology of extracting in combination hydrolysis in situ of acankoreanogenin from leaves of *Acanthopanax gracilistylus* W. W. Smith. J. Cent. South Univ..

[20] Shan B.E., Zeki K., Sugiura T., Yoshida Y., Yamashita U. (2000). Chinese medicinal herb, *Acanthopanax gracilistylus*, extract induces cell cycle arrest of human tumor cells in vitro. Cancer Sci..

[21] Shan B.E., Si C.Y., Zhang J.Z. (2004). The Isolation of Anti-tumor Component of *Acanthopanax gracilistylus*. Teratog. Carcinog. Mutagen..

[22] Li X.J., Dai L., Li Z., Zhang X.D., Liu X.Q., Zou Q.P., Xie X. (2015). Anti-inflammatory activities of lupane-triterpenoids in vitro and their phytochemical fingerprinting from leaves of *Acanthopanax gracilistylus*. Nat. Prod. Sci..

[23] Zou Q.P., Liu X.Q., Huang J.J., Yook C.S., Whang W.K., Lee H.K., Kwon O.K. (2017). Inhibitory effects of lupane-type triterpenoid saponins from the leaves of *Acanthopanax gracilistylus* on lipopolysaccharide-induced TNF-α, IL-1β and high-mobility group box 1 release in macrophages. Mol. Med. Rep..

[24] Liu X.Q., Zou Q.P., Huang J.J., Yook C.S., Whang W.K., Lee H.K., Kwon O.K. (2017). Inhibitory effects of 3α-hydroxy-lup-20(29)-en-23, 28-dioic acid on lipopolysaccharide-induced TNF-α, IL-1β, and the high mobility group box 1 release in macrophages. Biosci. Biotechnol. Biochem..

[25] Yook C.S., Kim I.H., Hahn D.R., Nohara T., Chang S.Y. (1998). A lupane-triterpene glycoside from leaves of two *Acanthopanax*. Phytochemistry.

[26] Chang S.Y., Yook C.S., Nohara T. (1998). Two new lupane-triterpene glycosides from leaves of *Acanthopanax koreanum*. Chem. Pharm. Bull..

[27] Nhiem N.X., Tung N.H., Van Kiem P., Van Minh C., Ding Y., Hyun J.H., Kang H.K., Kim Y.H. (2009). Lupane triterpene glycosides from leave of *Acanthopanax koreanum* and their cytotoxic activity. Chem. Pharm. Bull..

[28] Nhiem N.X., Van Kiem P., Van Minh C., Tai B.H., Yen P.H., Tung N.H., Tung N.H., Hyun J.H., Kang H.K., Kim Y.H. (2010). Lupane-type triterpene glycosides from the leaves of *Acanthopanax koreanum* and their in vitro cytotoxicity. Planta Med..

[29] Jahan N., Ahmed W., Malik A. (1995). A lupene-type triterpene from *Mimusops elengi*. Phytochemistry.

[30] Lischewski M., Ty P.D., Schmidt J., Preiss A., Phiet H.V., Adam G. (1984). Natural products from Vietnamese plants. Part 8. 3α,11α-Dihydroxylup-20(29)-ene-23,28-dioic acid from *Schefflera octophylla*. Phytochemistry.

[31] Chang S.Y., Yook C.S., Nohara T. (1999). Lupane-triterpene glycosides from leaves of *Acanthopanax koreanum*. Phytochemistry.

[32] Titheradge M.A. (1998). The enzymatic measurement of nitrate and nitrite. Methods Mol. Biol..

