# Supplementary material for: Lupane Triterpenes from the Leaves of Acanthopanax gracilistylus
Source: Molecules. 2018 Jan 1;23(1):87. doi: 10.3390/molecules23010087 (PMC6017332; doi:10.3390/molecules23010087)
Supplement: Supplementary file 1 [file molecules-23-00087-s001.pdf]

Supporting Information

Lupane Triterpenes from Leave of *Acanthopanax gracilistylus*

Xiao-Jun Li [1,2,†], Qin-Peng Zou [3,†], Xiang Wang [1], Kwan-Woo Kim [2], Mao-Fang Lu [1], Sung-Kwon Ko [4], Chang-Soo Yook [5], Youn-Chul Kim [2,*], and Xiang-Qian Liu [1,*]

1 School of Pharmacy, Hunan University of Chinese Medicine, Changsha, Hunan 410208, China; 598237392@qq.com (X.-J.L.); 571340394@qq.com (X.W.); 79403002@qq.com (M.-F.L.); lxq0001cn@163.com (X.-Q.L.);

2 College of Pharmacy, Wonkwang University, Iksan 570-749, Korea; swamp1@naver.com (K.-W.K.); yckim@wku.ac.kr (Y.-C.K.);

3 Broad-Ocean Bio-Science and Technique Co., Ltd of Changsha, Hunan Changsha 410205, China; 511107871@qq.com (Q.-P.Z.)

4 Department of Oriental Medical Food & Nutrition, Semyung University, Jecheon 27136, Korea; skko@semyung.ac.kr (S.-K.K.)

5 School of Pharmacy, KyungHee University, Seoul 130-701, Korea; yookcs@khu.ac.kr (C.-S.Y.)

* Correspondence: lxq0001cn@163.com (X.-Q.L.); Tel.: +86-731-88458240 (X.-Q.L.); yckim@wku.ac.kr (Y.-C.K.); Tel.: +82-63-850-6823 (Y.-C.K.);

† X.-J.L. and Q.-P.Z. contribute equally to this work.

Contents of Supporting Information

NO.	Contents	Pages
1	**Figure S1.** Photo of *Acanthopanax gracilistylus* W. W. Smith	1
2	**Figure S2.** ^{1}H NMR spectrum of acangraciligenin S in acetone-d_6	2
3	**Figure S3.** Expand ^{1}H NMR spectrum of acangraciligenin S in acetone-d_6	2
4	**Figure S4.** Expand ^{1}H NMR spectrum of acangraciligenin S in acetone-d_6	3
5	**Figure S5.** ^{13}C NMR spectrum of acangraciligenin S in acetone-d_6	3
6	**Figure S6.** Expand ^{13}C NMR spectrum of acangraciligenin S in acetone-d_6	4
7	**Figure S7.** Expand ^{13}C NMR spectrum of acangraciligenin S in acetone-d_6	4
8	**Figure S8.** Expand ^{13}C NMR spectrum of acangraciligenin S in acetone-d_6	5
9	**Figure S9.** DEPT spectrum of acangraciligenin S in acetone-d_6	5
10	**Figure S10.** Expand DEPT spectrum of acangraciligenin S in acetone-d_6	6
11	**Figure S11.** HSQC spectrum of acangraciligenin S in acetone-d_6	6
12	**Figure S12.** Expand HSQC spectrum of acangraciligenin S in acetone-d_6	7
13	**Figure S13.** Expand HSQC spectrum of acangraciligenin S in acetone-d_6	7
14	**Figure S14.** HMBC spectrum of acangraciligenin S in acetone-d_6	8
15	**Figure S15.** Expand HMBC spectrum of acangraciligenin S in acetone-d_6	8
16	**Figure S16.** Expand HMBC spectrum of acangraciligenin S in acetone-d_6	9
17	**Figure S17.** ^{1}H-^{1}H COSY spectrum of acangraciligenin S in acetone-d_6	9
18	**Figure S18.** Expand ^{1}H-^{1}H COSY spectrum of acangraciligenin S in acetone-d_6	10
19	**Figure S19.** Expand ^{1}H-^{1}H COSY spectrum of acangraciligenin S in acetone-d_6	10
20	**Figure S20.** NOESY spectrum of acangraciligenin S in acetone-d_6	11
21	**Figure S21.** Expand NOESY spectrum of acangraciligenin S in acetone-d_6	11
22	**Figure S22.** Expand NOESY spectrum of acangraciligenin S in acetone-d_6	12
23	**Figure S23.** ESIMS spectrum of acangraciligenin S	13
24	**Figure S24.** HRESIMS spectrum of acangraciligenin S	13
25	**Figure S25.** ^{1}H NMR spectrum of acangraciliside S in methanol-d_4	14
26	**Figure S26.** Expand ^{1}H NMR spectrum of acangraciliside S in methanol-d_4	14
27	**Figure S27.** Expand ^{1}H NMR spectrum of acangraciliside S in methanol-d_4	15
28	**Figure S28.** Expand ^{1}H NMR spectrum of acangraciliside S in methanol-d_4	15
29	**Figure S29.** ^{13}C NMR spectrum of acangraciliside S in methanol-d_4	16
30	**Figure S30.** Expand ^{13}C NMR spectrum of acangraciliside S in methanol-d_4	16
31	**Figure S31.** Expand ^{13}C NMR spectrum of acangraciliside S in methanol-d_4	17
32	**Figure S32.** HSQC spectrum of acangraciliside S in methanol-d_4	17
33	**Figure S33.** Expand HSQC spectrum of acangraciliside S in methanol-d_4	18
34	**Figure S34.** Expand HSQC spectrum of acangraciliside S in methanol-d_4	18
35	**Figure S35.** HMBC spectrum of acangraciliside S in methanol-d_4	19
36	**Figure S36.** Expand HMBC spectrum of acangraciliside S in methanol-d_4	19
37	**Figure S37.** Expand HMBC spectrum of acangraciliside S in methanol-d_4	20
38	**Figure S38.** Expand HMBC spectrum of acangraciliside S in methanol-d_4	20
39	**Figure S39.** ^{1}H-^{1}H COSY spectrum of acangraciliside S in methanol-d_4	21
40	**Figure S40.** Expand ^{1}H-^{1}H COSY spectrum of acangraciliside S in methanol-d_4	21
41	**Figure S41.** Expand ^{1}H-^{1}H COSY spectrum of acangraciliside S in methanol-d_4	22
42	**Figure S42.** Expand ^{1}H-^{1}H COSY spectrum of acangraciliside S in methanol-d_4	22

**43**	**Figure S43.** NOESY spectrum of acangraciliside S in methanol-d_4	**23**
**44**	**Figure S44.** Expand NOESY spectrum of acangraciliside S in methanol-d_4	**23**
**45**	**Figure S45.** Expand NOESY spectrum of acangraciliside S in methanol-d_4	**24**
**46**	**Figure S46.** ESIMS spectrum of acangraciliside S	**25**
**47**	**Figure S47.** HRESIMS spectrum of acangraciliside S	**25**
**48**	**Figure S48.** ^{1}H NMR spectrum of **2a** in acetone-d_6	**26**
**49**	**Figure S49.** Expand ^{1}H NMR spectrum of **2a** in acetone-d_6	**26**
**50**	**Figure S50.** Expand ^{1}H NMR spectrum of **2a** in acetone-d_6	**27**
**51**	**Figure S51.** ^{13}C NMR spectrum of **2a** in acetone-d_6	**27**
**52**	**Figure S52.** Expand ^{13}C NMR spectrum of **2a** in acetone-d_6	**28**
**53**	**Figure S53.** Expand ^{13}C NMR spectrum of **2a** in acetone-d_6	**28**
**54**	**Figure S54.** Expand ^{13}C NMR spectrum of **2a** in acetone-d_6	**29**
**55**	**Figure S55.** DEPT spectrum of **2a** in acetone-d_6	**29**
**56**	**Figure S56.** Expand DEPT spectrum of **2a** in acetone-d_6	**30**
**57**	**Figure S57.** ^{1}H NMR spectrum of 3α,11α-dihydroxy-lup-20(29)-en-23,28-dioic acid in acetone-d_6	**30**
**58**	**Figure S58.** Expand ^{1}H NMR spectrum of 3α,11α-dihydroxy-lup-20(29)-en-23,28-dioic acid in acetone-d_6	**31**
**59**	**Figure S59.** Expand ^{1}H NMR spectrum of 3α,11α-dihydroxy-lup-20(29)-en-23,28-dioic acid in acetone-d_6	**31**
**60**	**Figure S60.** ^{13}C NMR spectrum of 3α,11α-dihydroxy-lup-20(29)-en-23,28-dioic acid in acetone-d_6	**32**
**61**	**Figure S61.** Expand ^{13}C NMR spectrum of 3α,11α-dihydroxy-lup-20(29)-en-23,28-dioic acid in acetone-d_6	**32**
**62**	**Figure S62.** Expand ^{13}C NMR spectrum of 3α,11α-dihydroxy-lup-20(29)-en-23,28-dioic acid in acetone-d_6	**33**
**63**	**Figure S63.** Expand ^{13}C NMR spectrum of 3α,11α-dihydroxy-lup-20(29)-en-23,28-dioic acid in acetone-d_6	**33**
**64**	**Figure S64.** DEPT spectrum of 3α,11α-dihydroxy-lup-20(29)-en-23,28-dioic acid in acetone-d_6	**34**
**65**	**Figure S65.** Expand DEPT spectrum of 3α,11α-dihydroxy-lup-20(29)-en-23,28-dioic acid in acetone-d_6	**34**
**66**	**Figure S66.** Expand DEPT spectrum of 3α,11α-dihydroxy-lup-20(29)-en-23,28-dioic acid in acetone-d_6	**35**
**67**	**Figure S67.** HSQC spectrum of 3α,11α-dihydroxy-lup-20(29)-en-23,28-dioic acid in acetone-d_6	**35**
**68**	**Figure S68.** Expand HSQC spectrum of 3α,11α-dihydroxy-lup-20(29)-en-23,28-dioic acid in acetone-d_6	**36**
**69**	**Figure S69.** Expand HSQC spectrum of 3α,11α-dihydroxy-lup-20(29)-en-23,28-dioic acid in acetone-d_6	**36**
**70**	**Figure S70.** HMBC spectrum of 3α,11α-dihydroxy-lup-20(29)-en-23,28-dioic acid in acetone-d_6	**37**
**71**	**Figure S71.** Expand HMBC spectrum of 3α,11α-dihydroxy-lup-20(29)-en-23,28-dioic acid in acetone-d_6	**37**
**72**	**Figure S72.** Expand HMBC spectrum of 3α,11α-dihydroxy-lup-20(29)-en-23,28-dioic acid in acetone-d_6	**38**
**73**	**Figure S73.** Expand HMBC spectrum of 3α,11α-dihydroxy-lup-20(29)-en-23,28-dioic acid in acetone-d_6	**38**
**74**	**Figure S74.**^{1}H-^{1}H COSY spectrum of 3α,11α-dihydroxy-lup-20(29)-en-23,28-dioic acid in acetone-d_6	**39**

**75**	**Figure S75.** Expand ^{1}H-^{1}H COSY spectrum of 3*α*,11*α*-dihydroxy-lup-20(29)-en-23,28-dioic acid in acetone-d_6	**39**
**76**	**Figure S76.** Expand ^{1}H-^{1}H COSY spectrum of 3*α*,11*α*-dihydroxy-lup-20(29)-en-23,28-dioic acid in acetone-d_6	**40**
**77**	**Figure S77.** NOESY spectrum of 3*α*,11*α*-dihydroxy-lup-20(29)-en-23,28-dioic acid in acetone-d_6	**40**
**78**	**Figure S78.** Expand NOESY spectrum of 3*α*,11*α*-dihydroxy-lup-20(29)-en-23,28-dioic acid in acetone-d_6	**41**
**79**	**Figure S79.** ^{1}H NMR spectrum of acankoreoside C in methanol-d_4	**41**
**80**	**Figure S80.** Expand ^{1}H NMR spectrum of acankoreoside C in methanol-d_4	**42**
**81**	**Figure S81.** Expand ^{1}H NMR spectrum of acankoreoside C in methanol-d_4	**42**
**82**	**Figure S82.** Expand ^{1}H NMR spectrum of acankoreoside C in methanol-d_4	**43**
**83**	**Figure S83.** ^{13}C NMR spectrum of acankoreoside C in methanol-d_4	**43**
**84**	**Figure S84.** Expand ^{13}C NMR spectrum of acankoreoside C in methanol-d_4	**44**
**85**	**Figure S85.** Expand ^{13}C NMR spectrum of acankoreoside C in methanol-d_4	**44**
**86**	**Table S1.** NMR spectral data of compound **1** in acetone-d_6	**45**
**87**	**Table S2.** NMR spectral data of compound **2** in methanol-d_4	**45**
**88**	**Table S3.** NMR spectral data of compounds **3-4**	**46**

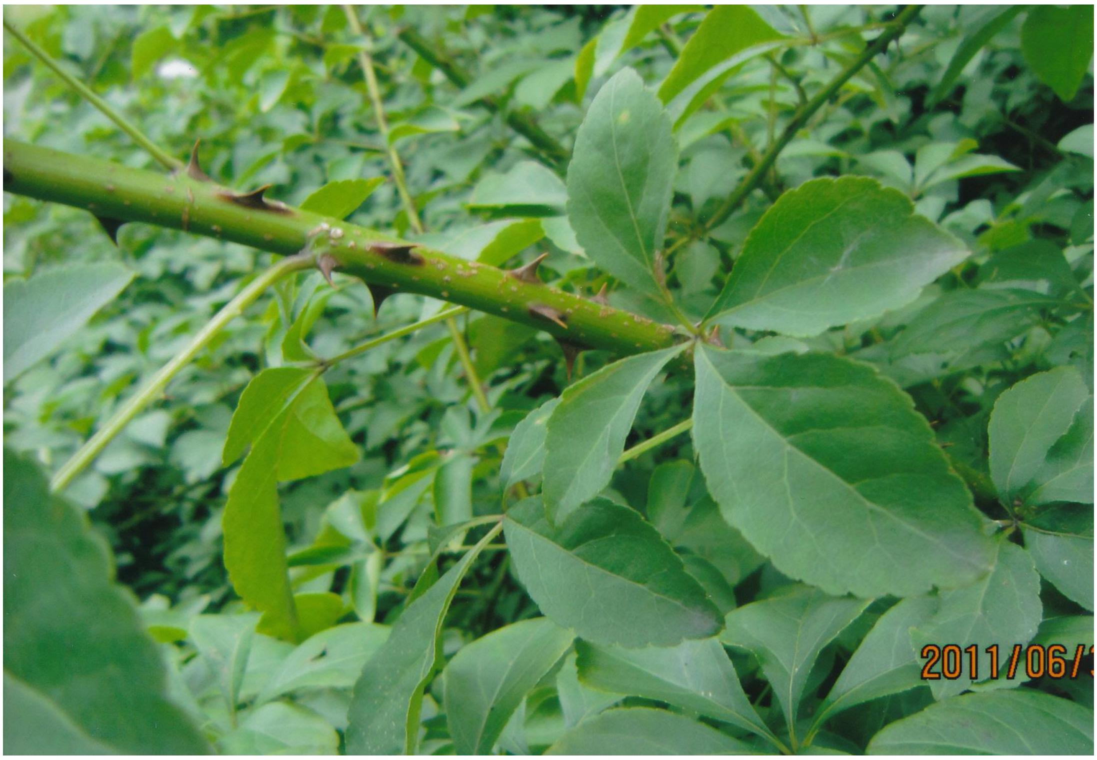

Figure S1. Photo of *Acanthopanax gracilistylus* W. W. Smith (Changsha City, Hunan Prov., by Liu Xiang-qian)

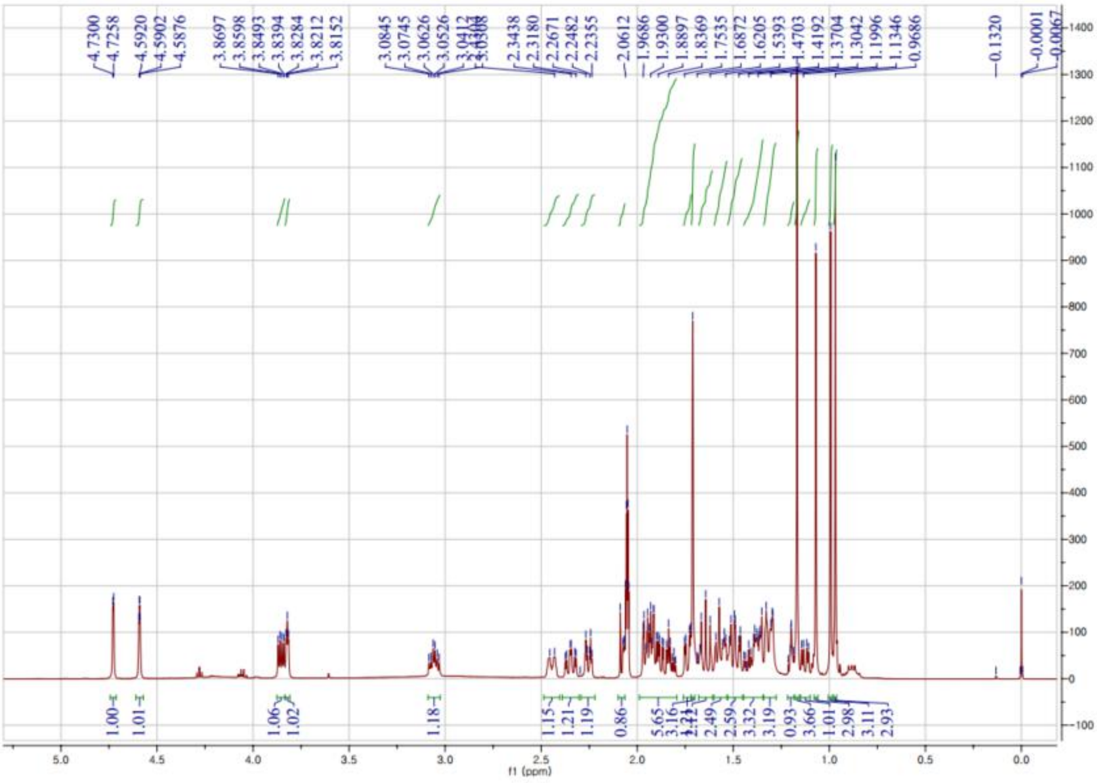

Figure S2. ^{1}H NMR spectrum of acangraciligenin S in acetone-d_6

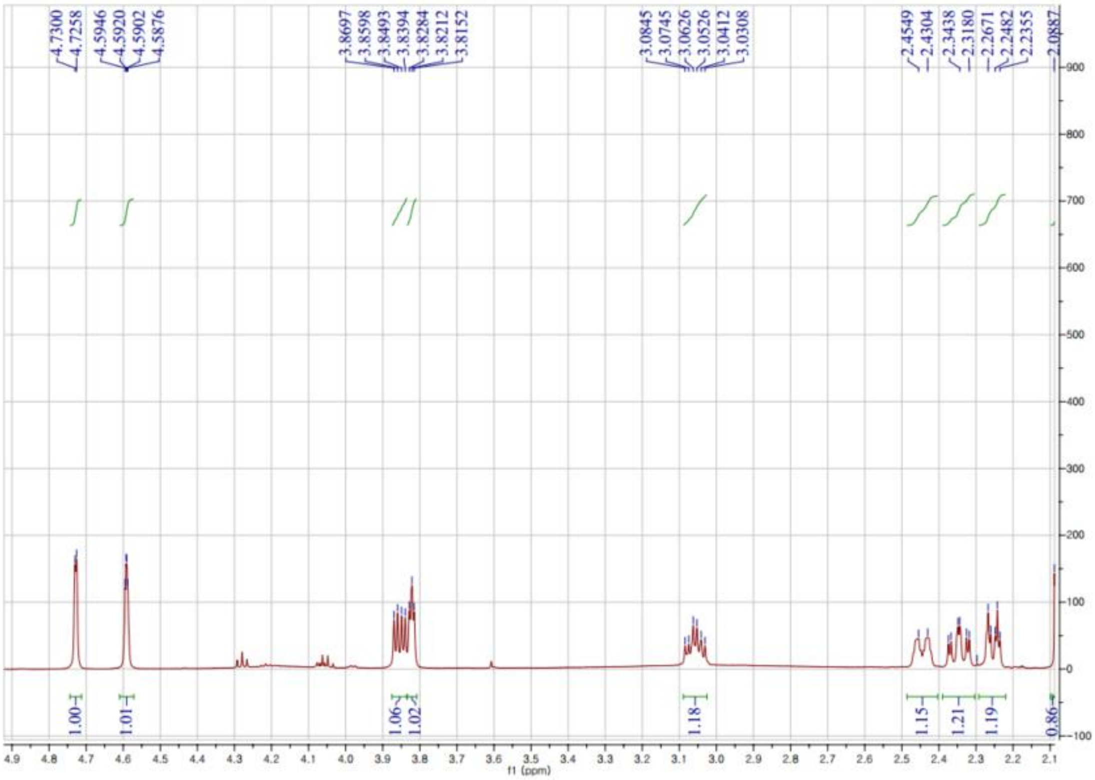

Figure S3. Expand ^{1}H NMR spectrum of acangraciligenin S in acetone-d_6

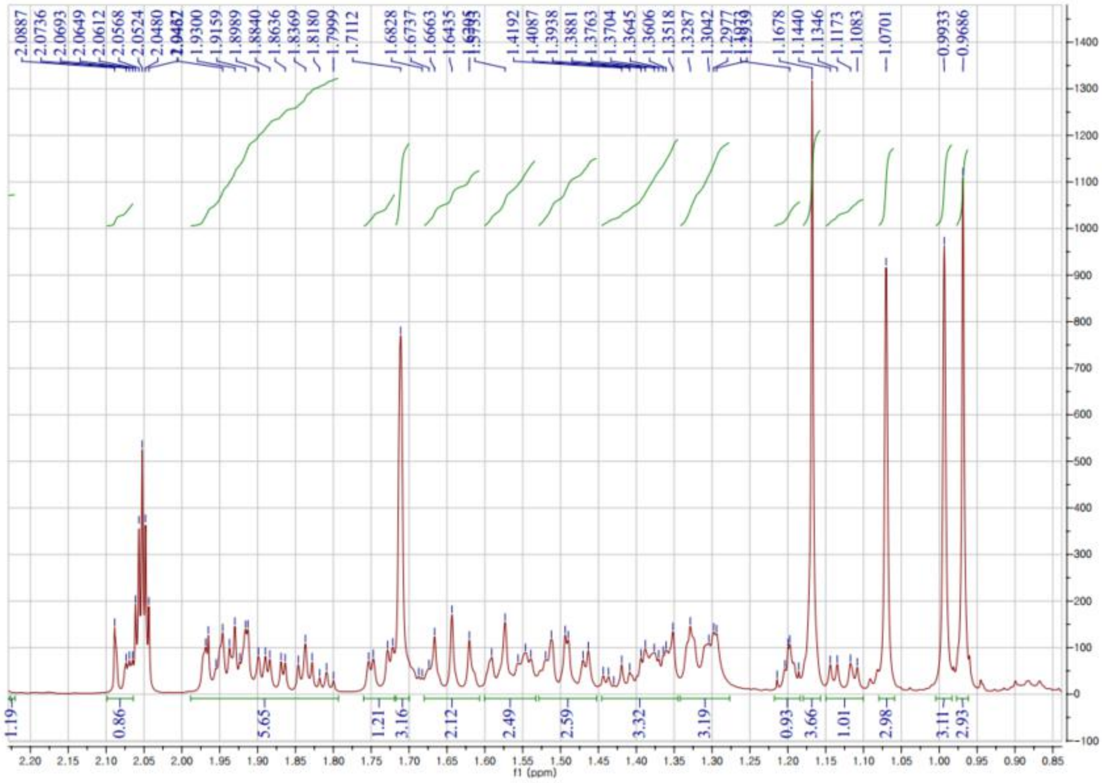

Figure S4. Expand ^{1}H NMR spectrum of acangraciligenin S in acetone-d_6

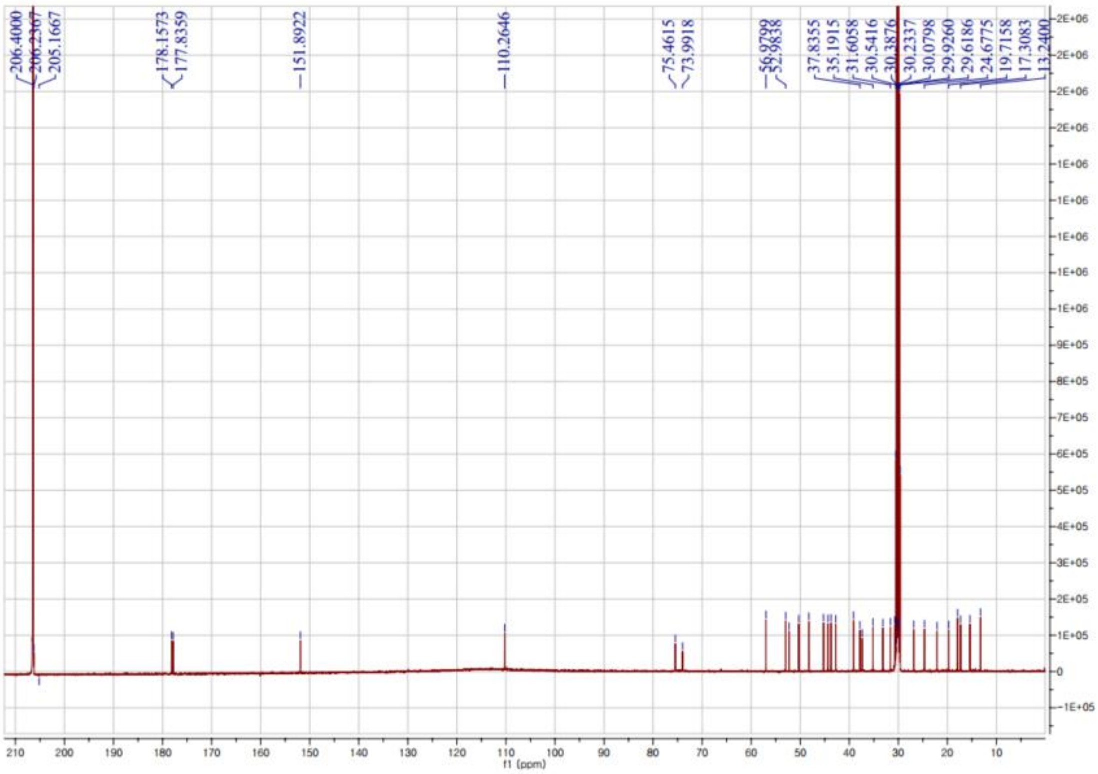

Figure S5. ^{13}C NMR spectrum of acangraciligenin S in acetone-d_6

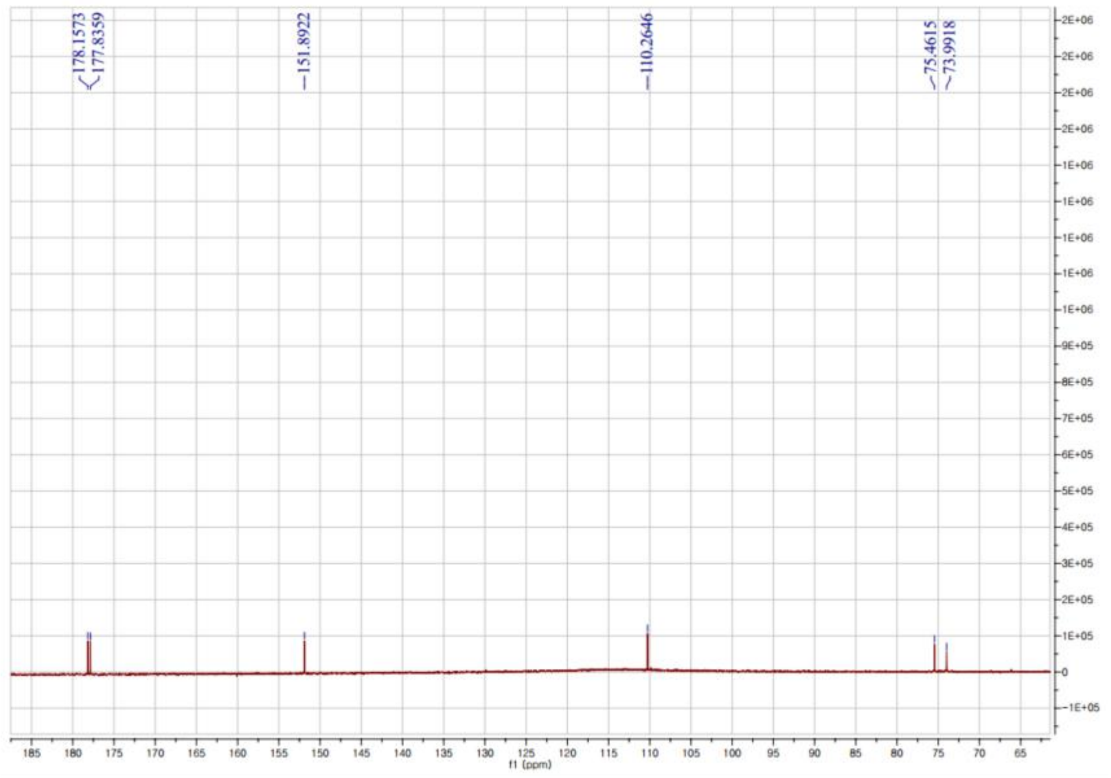

Figure S6. Expand ^{13}C NMR spectrum of acangraciligenin S in acetone-d_6

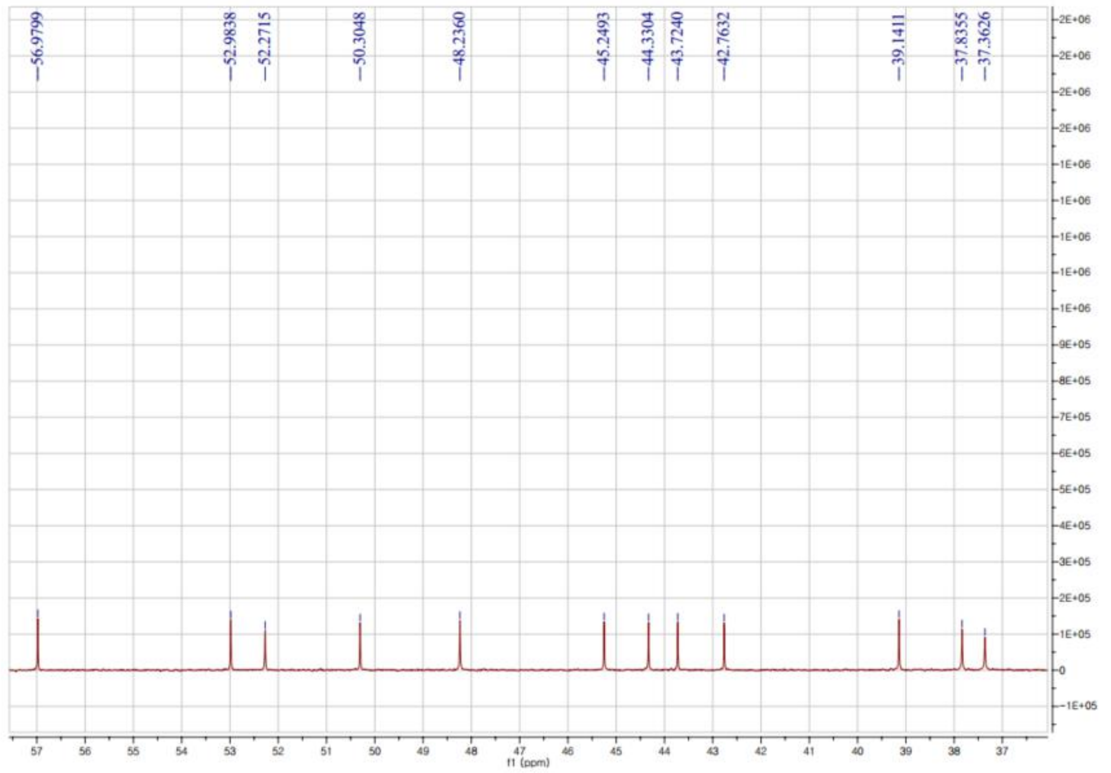

Figure S7. Expand ^{13}C NMR spectrum of acangraciligenin S in acetone-d_6

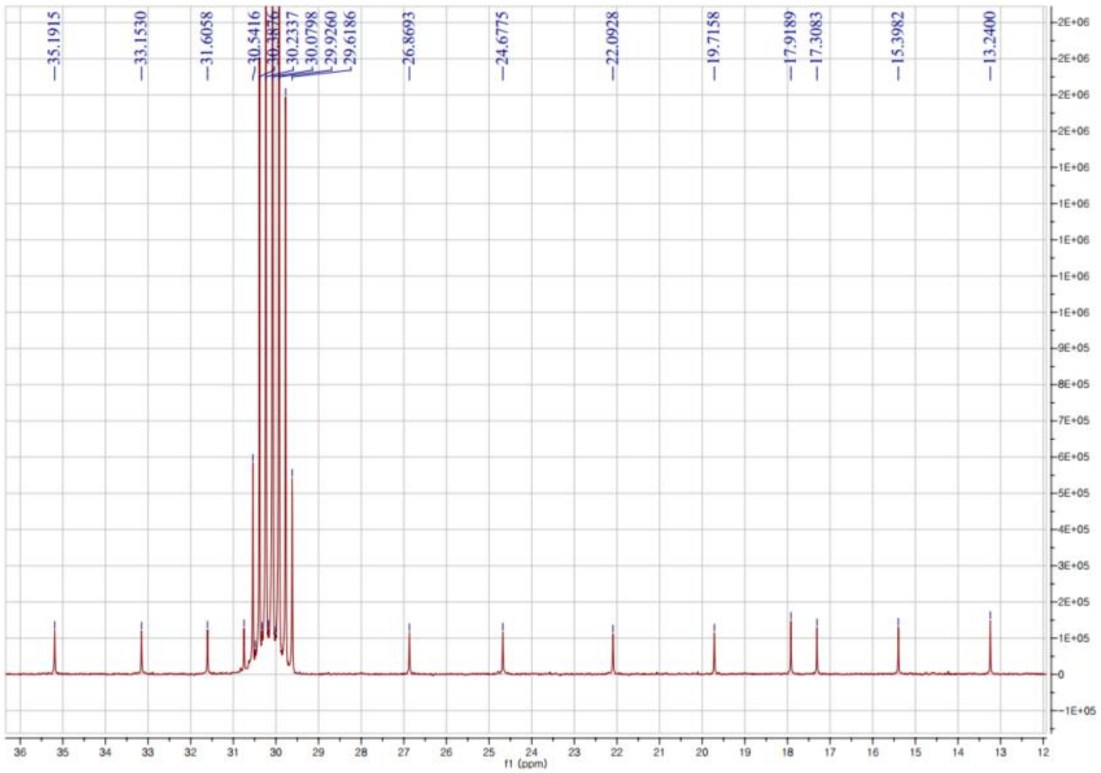

Figure S8. Expand ^{13}C NMR spectrum of acangraciligenin S in acetone-d_6

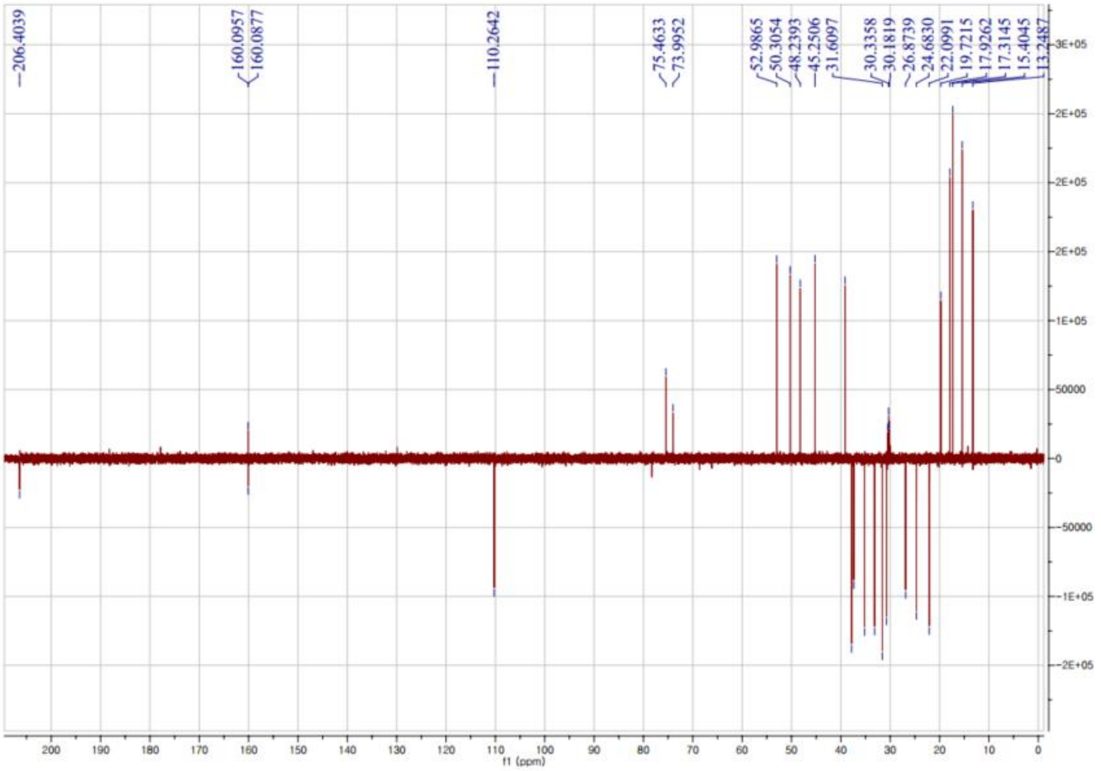

Figure S9. DEPT spectrum of acangraciligenin S in acetone-d_6

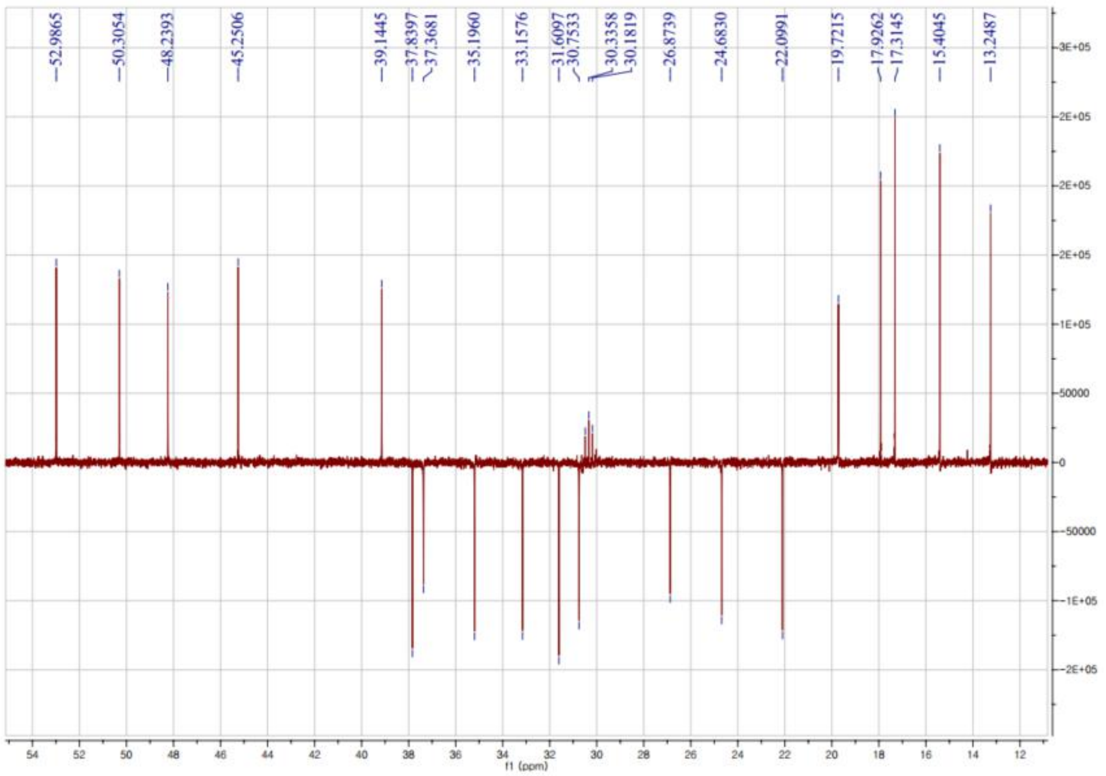

Figure S10. Expand DEPT spectrum of acangraciligenin S in acetone-d_6

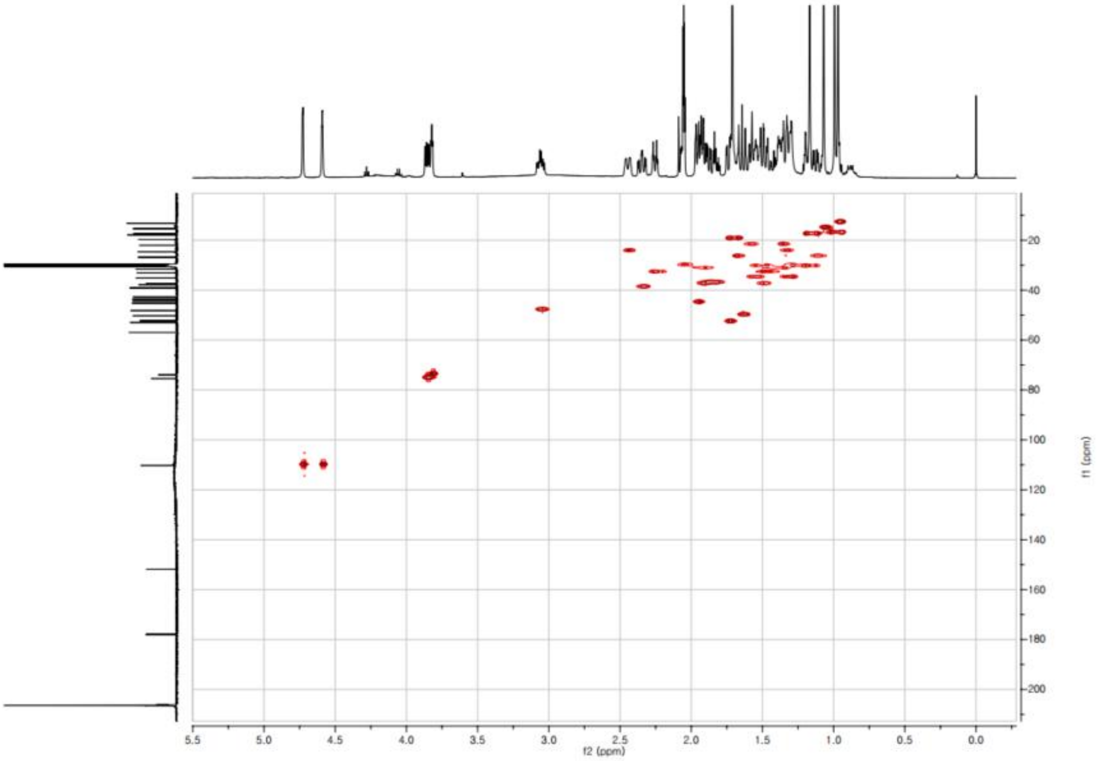

Figure S11. HSQC spectrum of acangraciligenin S in acetone-d_6

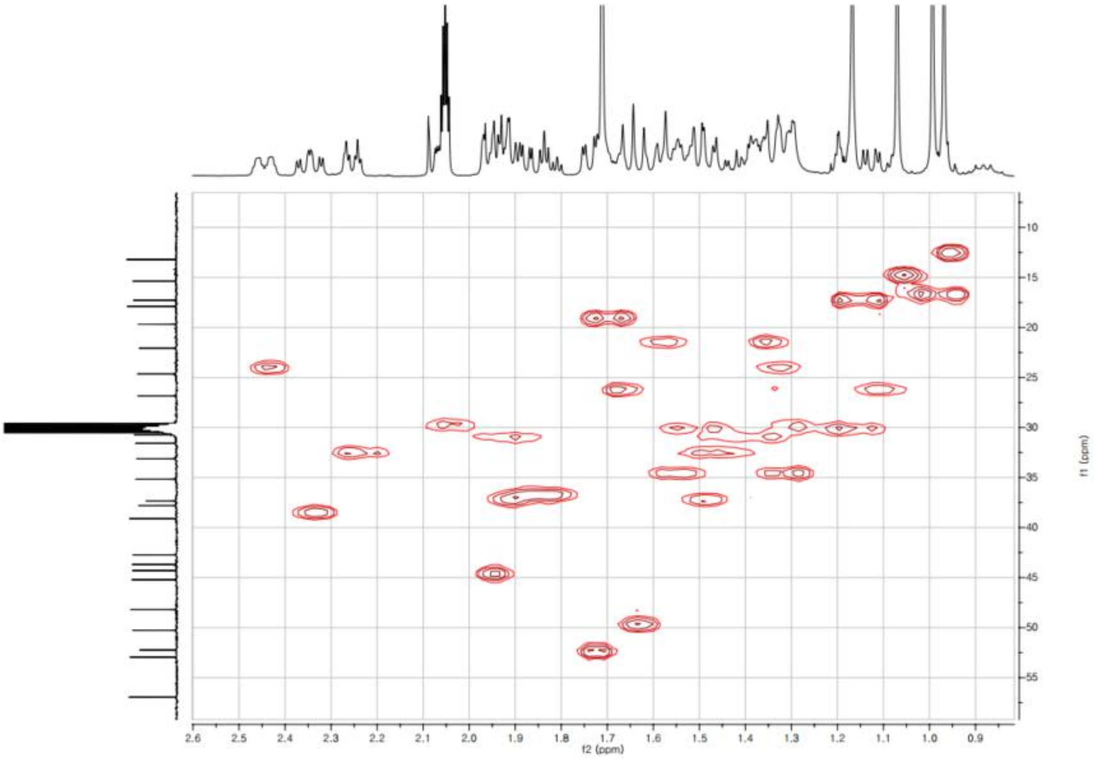

Figure S12. Expand HSQC spectrum of acangraciligenin S in acetone-d_6

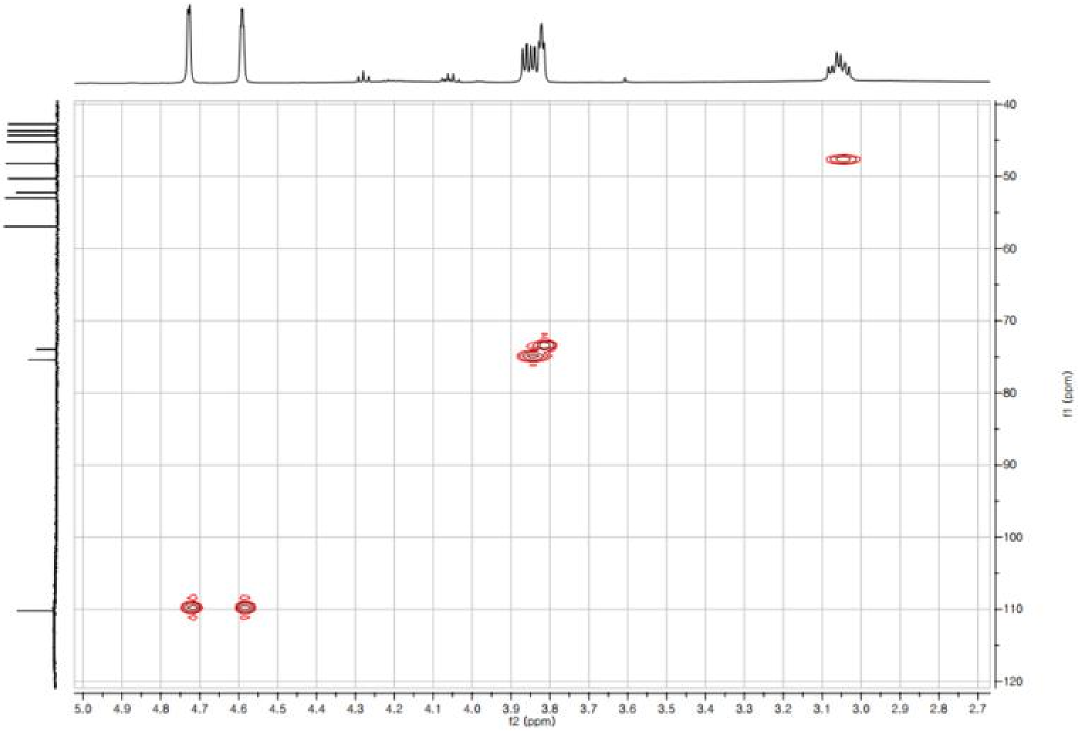

Figure S13. Expand HSQC spectrum of acangraciligenin S in acetone-d_6

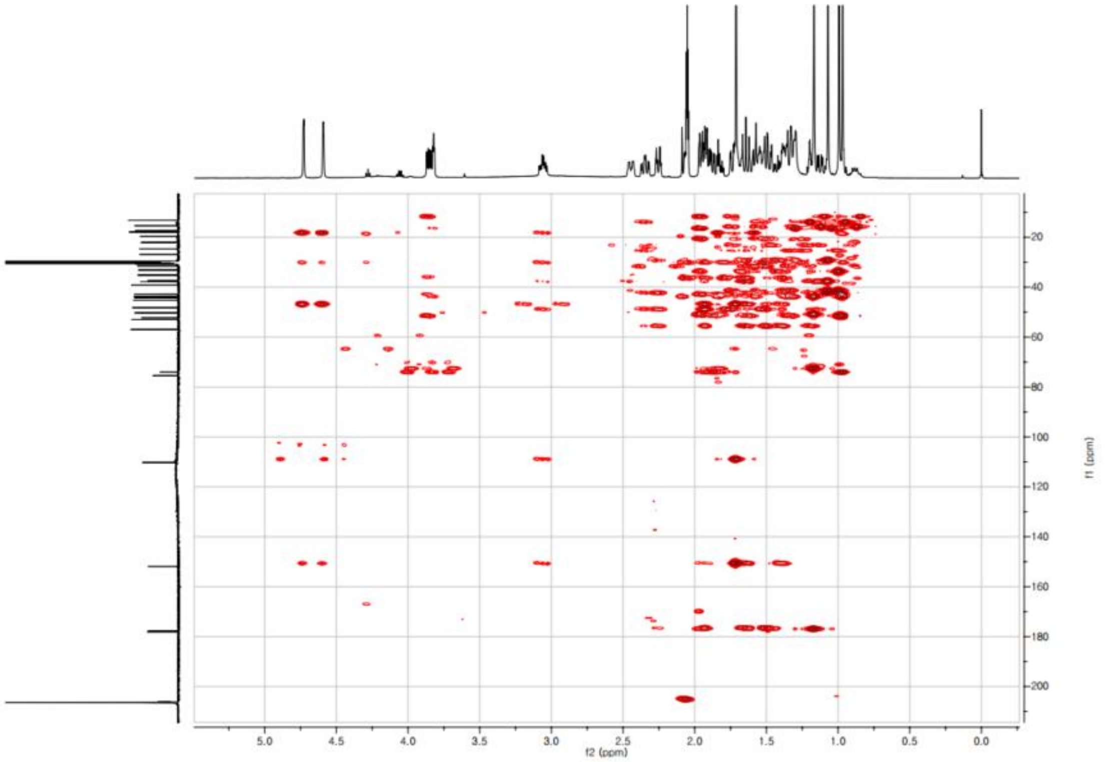

Figure S14. HMBC spectrum of acangraciligenin S in acetone-d_6

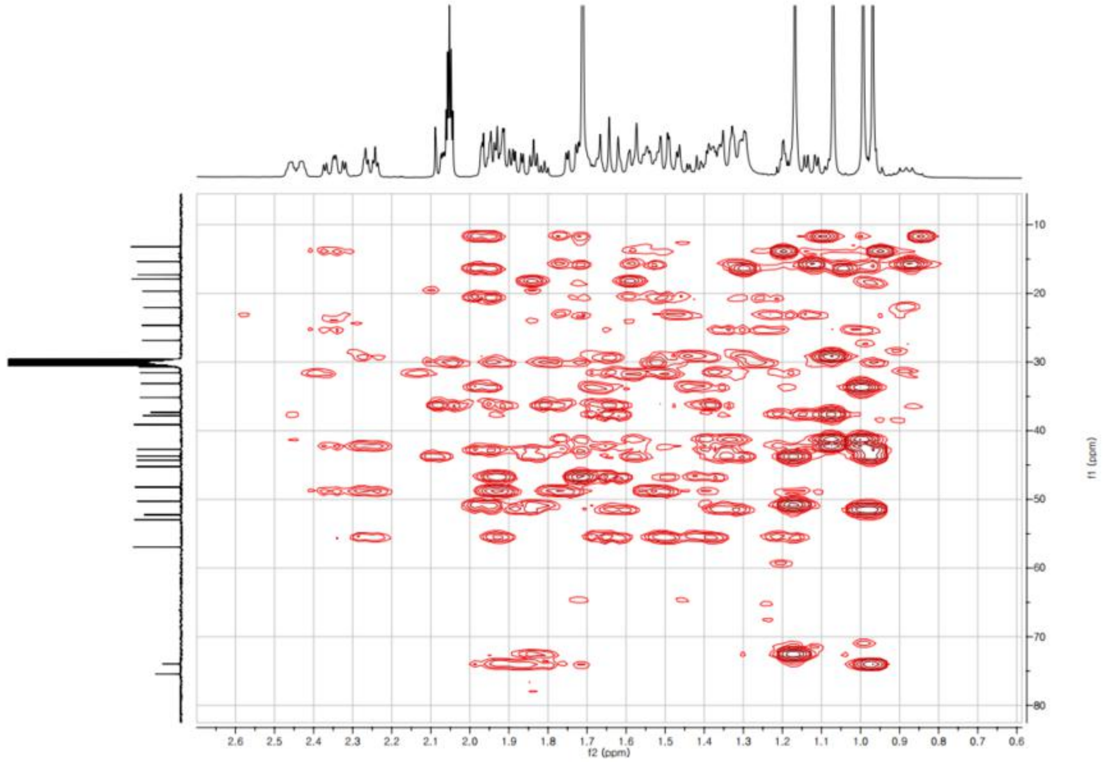

Figure S15. Expand HMBC spectrum of acangraciligenin S in acetone-d_6

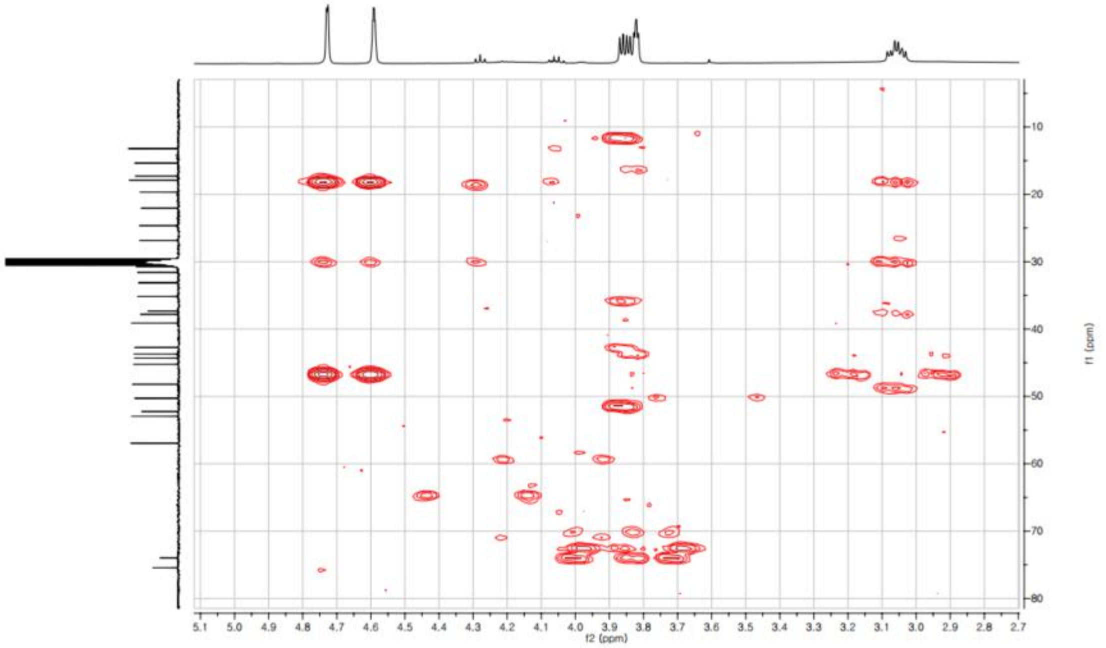

Figure S16. Expand HMBC spectrum of acangraciligenin S in acetone-d_6

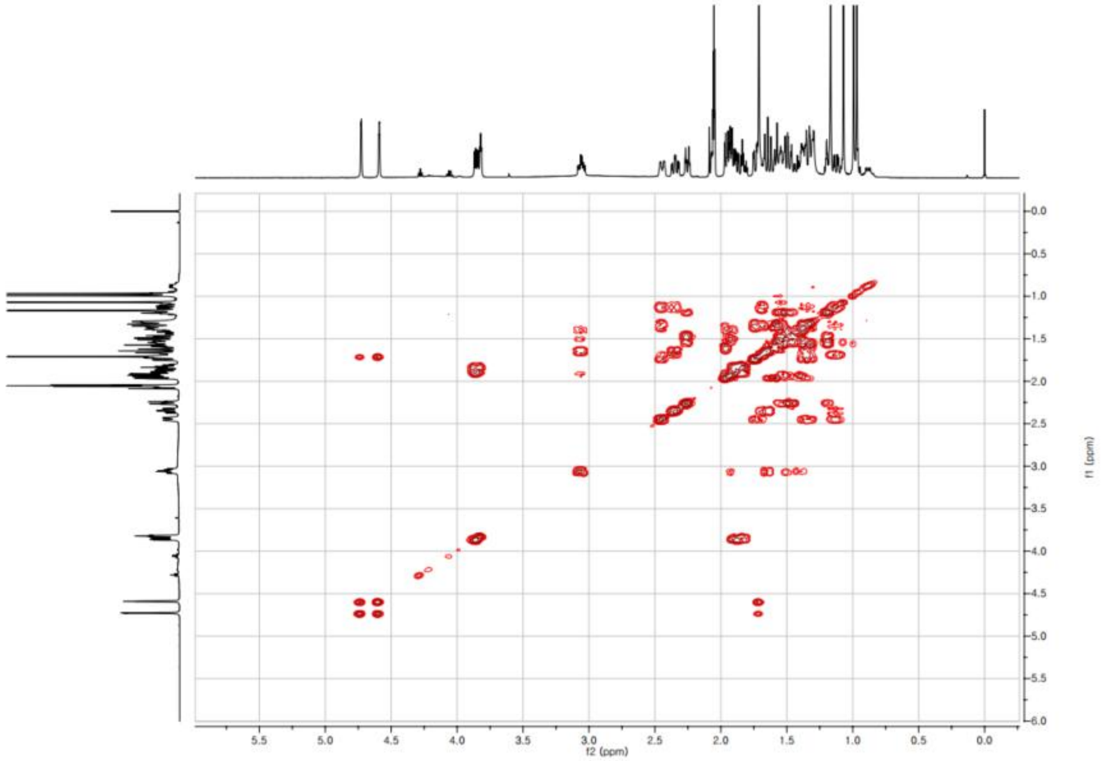

Figure S17. ^{1}H-^{1}H COSY spectrum of acangraciligenin S in acetone-d_6

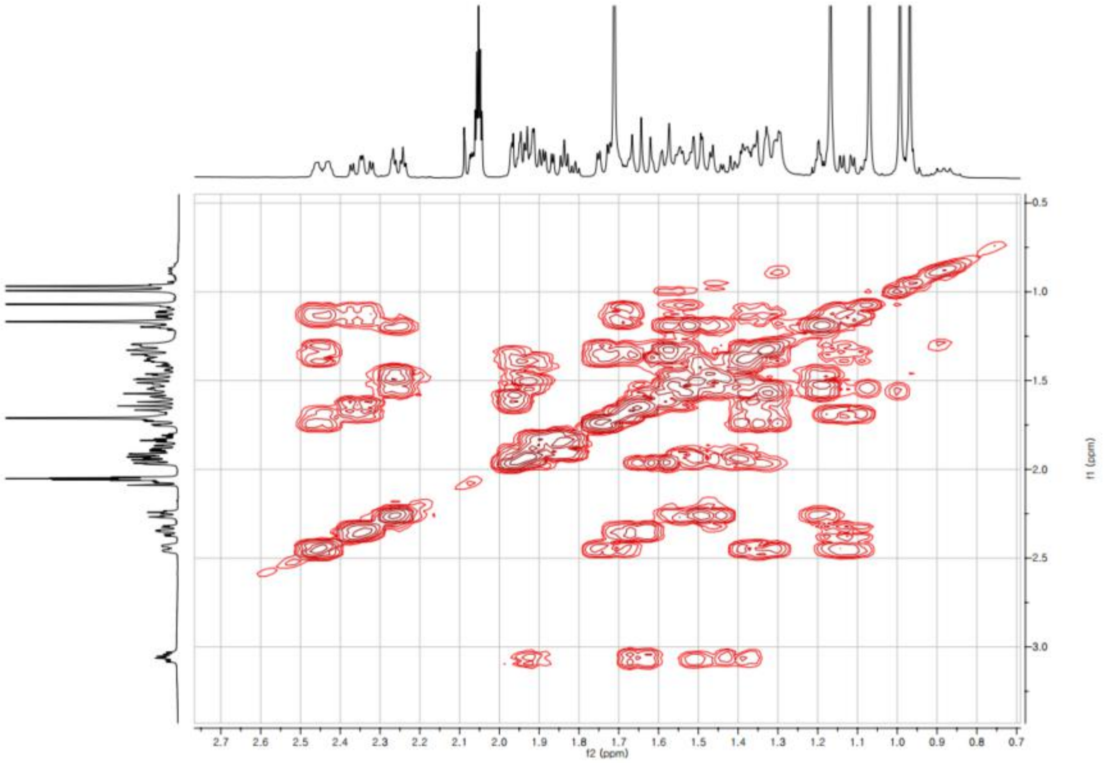

Figure S18. Expand ^{1}H-^{1}H COSY spectrum of acangraciligenin S in acetone-d_6

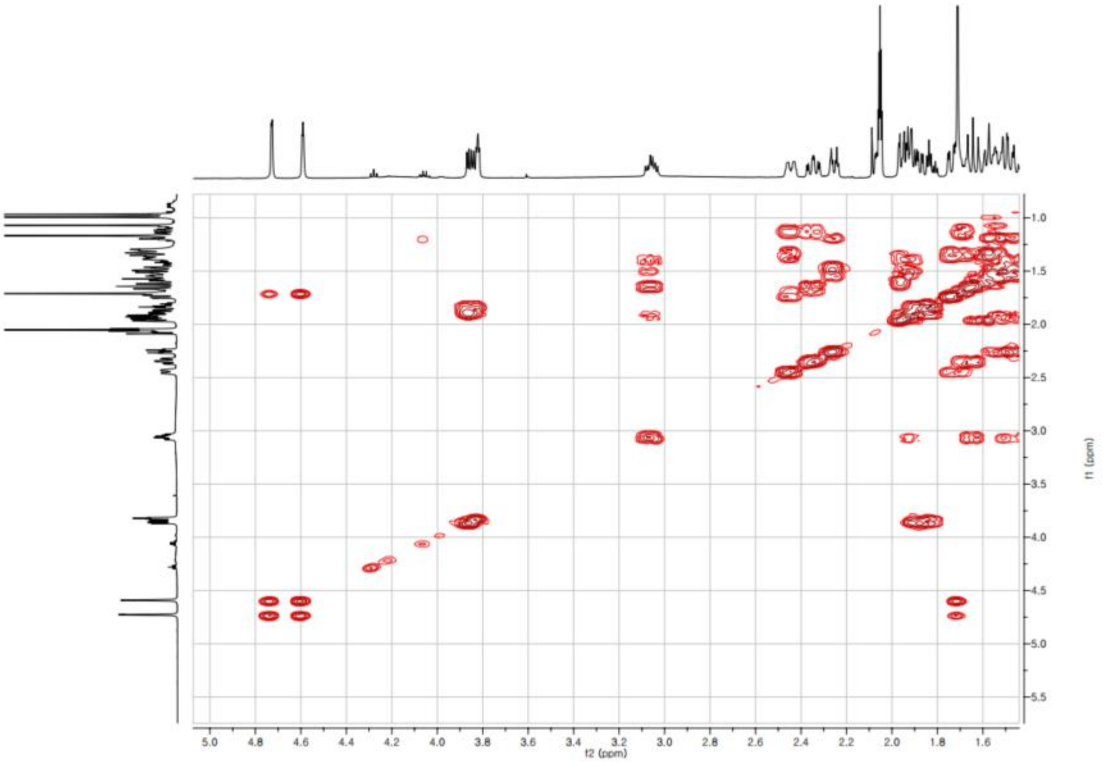

Figure S19. Expand ^{1}H-^{1}H COSY spectrum of acangraciligenin S in acetone-d_6

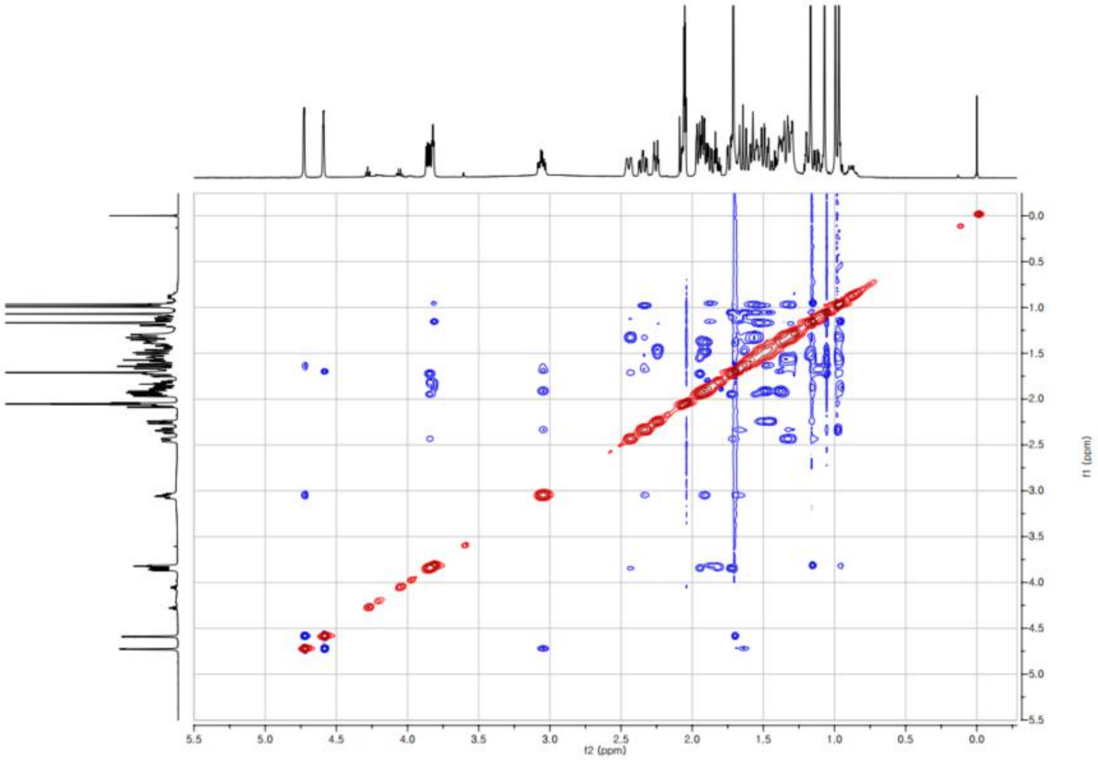

Figure S20. NOESY spectrum of acangraciligenin S in acetone-d_6

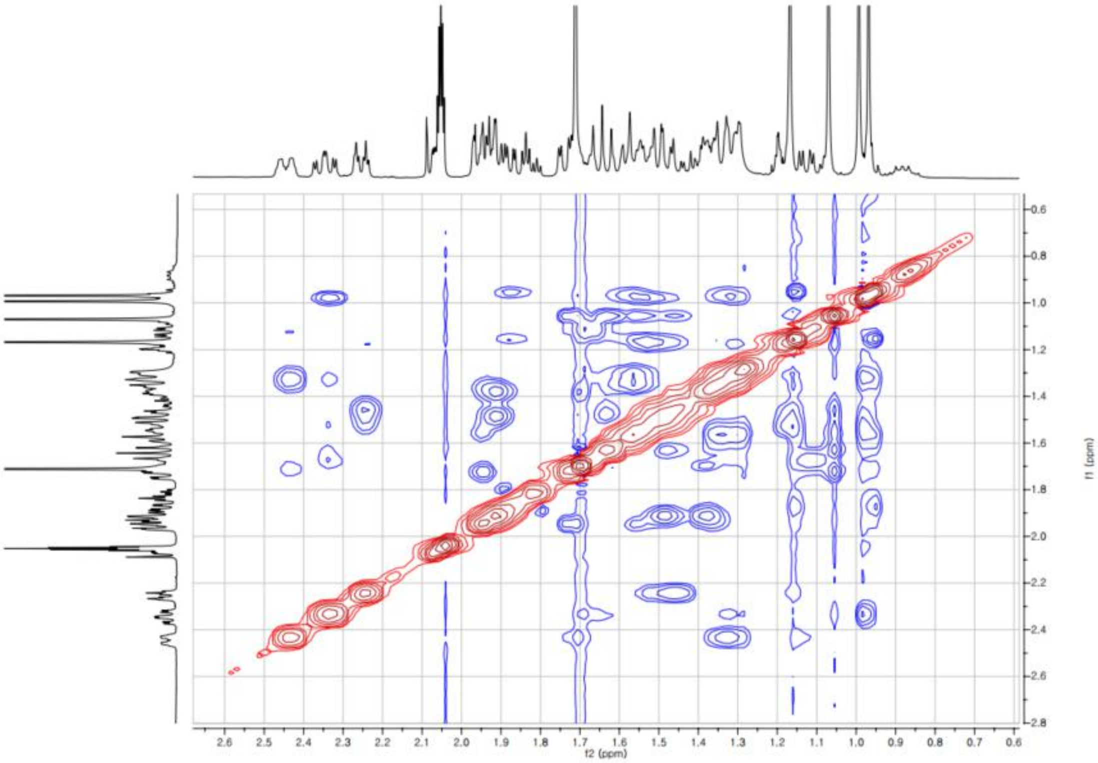

Figure S21. Expand NOESY spectrum of acangraciligenin S in acetone-d_6

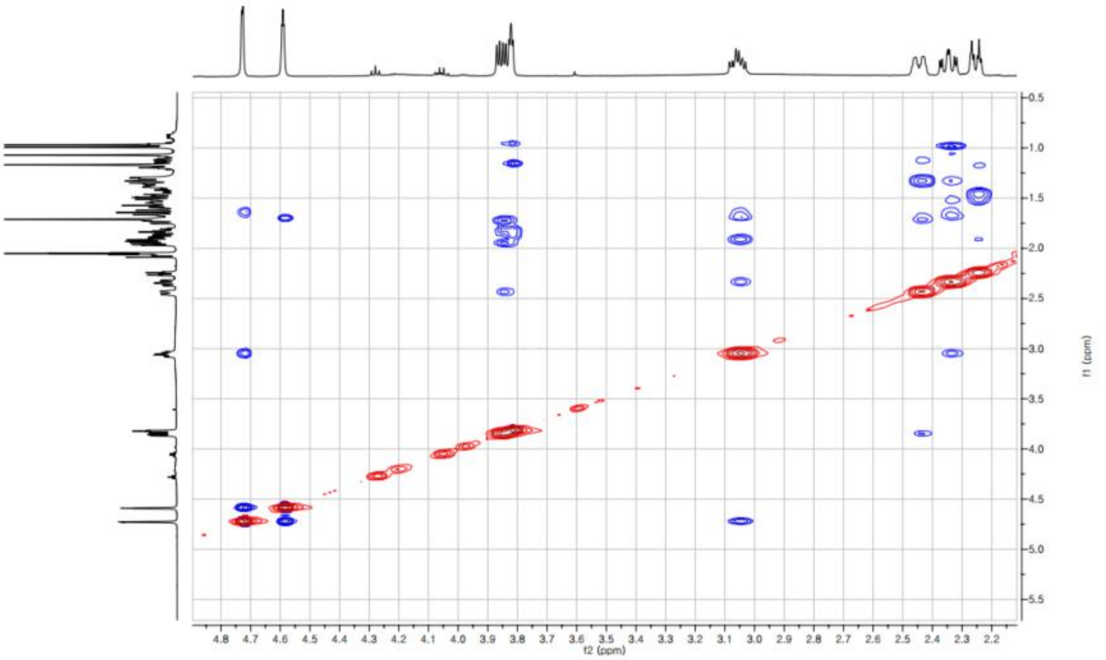

Figure S22. Expand NOESY spectrum of acangraciligenin S in acetone-d_6

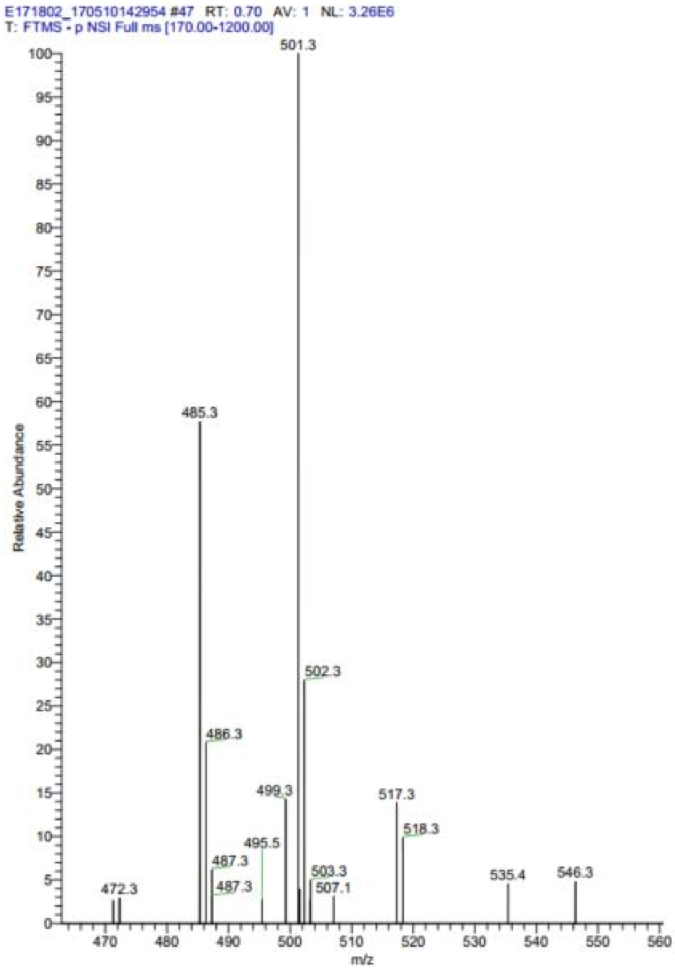

Figure S23. ESIMS spectrum of acangraciligenin S

National Center for Organic Mass Spectrometry in Shanghai
Shanghai Institute of Organic Chemistry
Chinese Academic of Sciences
High Resolution MS DATA REPORT

Instrument: Thermo Fisher Scientific LTQ FTICR

Card Serial Number : E171801

Sample Serial Number: EA-1

Operator : ZHUFJ Date: 2017/05/11

Operation Mode: DART Negative

Elemental composition search on mass 501.32

m/z= 496.32-506.32

m/z	Theo. Mass	Delta (ppm)	RDB equiv.	Composition
501.3221	501.3222	-0.20	8.5	$C_{30}H_{45}O_6$
	501.3181	7.82	4.5	$C_{25}H_{45}O_8N_2$

Figure S24. HRESIMS spectrum of acangraciligenin S

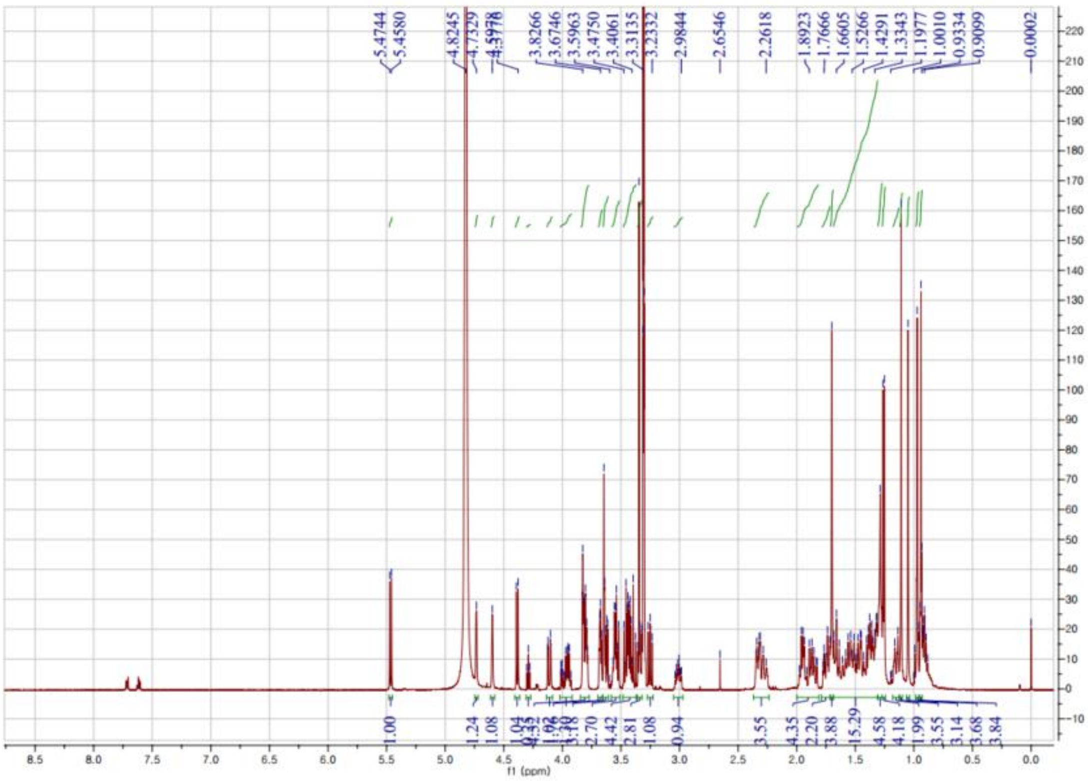

Figure S25. ^{1}H NMR spectrum of acangraciliside S in methanol-d_4

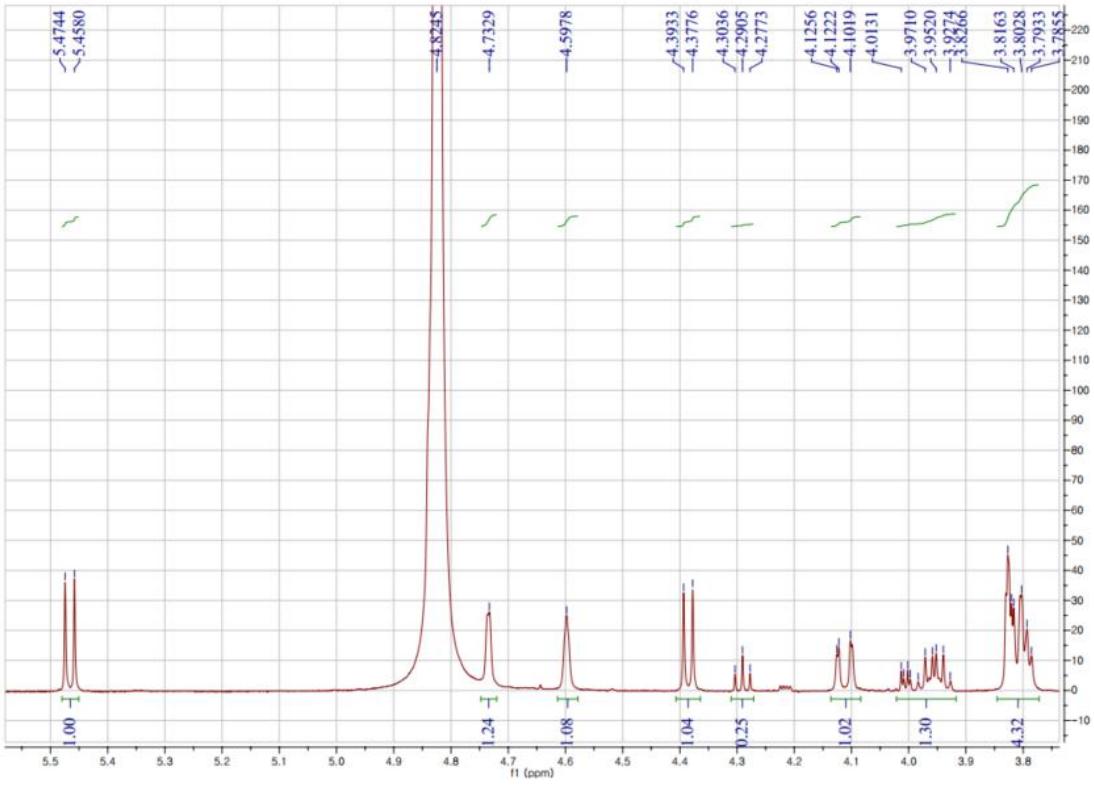

Figure S26. Expand ^{1}H NMR spectrum of acangraciliside S in methanol-d_4

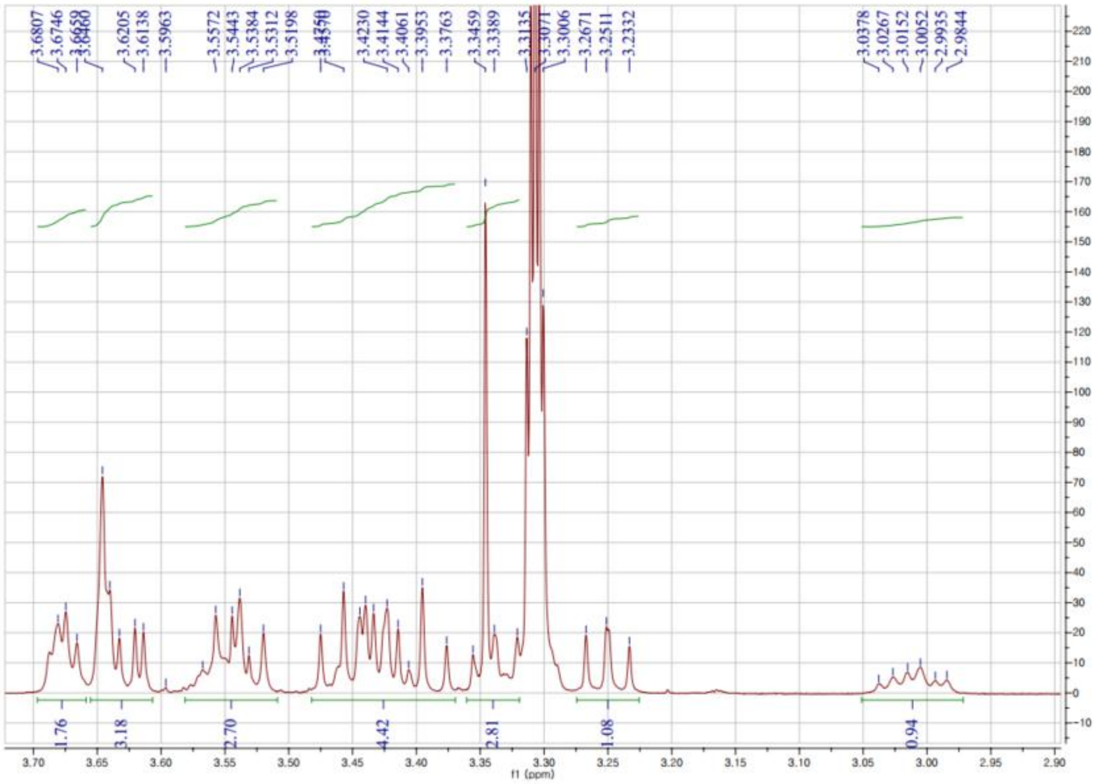

Figure S27. Expand ^{1}H NMR spectrum of acangraciliside S in methanol-d_4

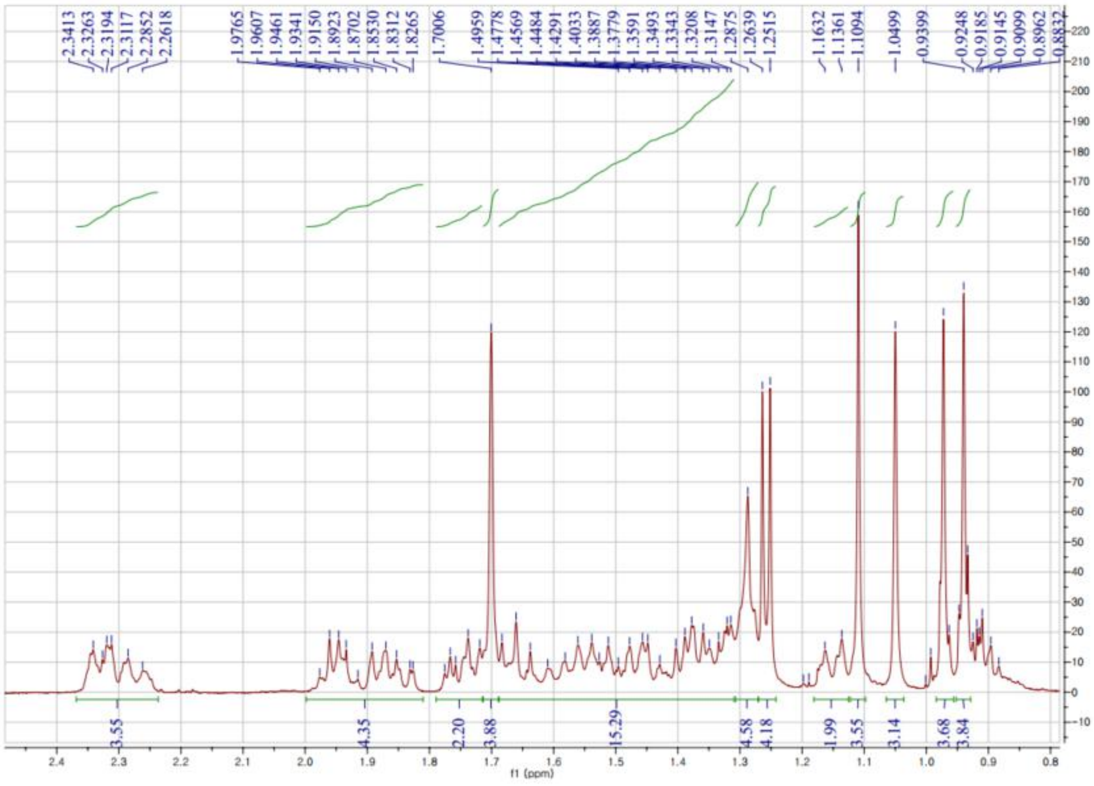

Figure S28. Expand ^{1}H NMR spectrum of acangraciliside S in methanol-d_4

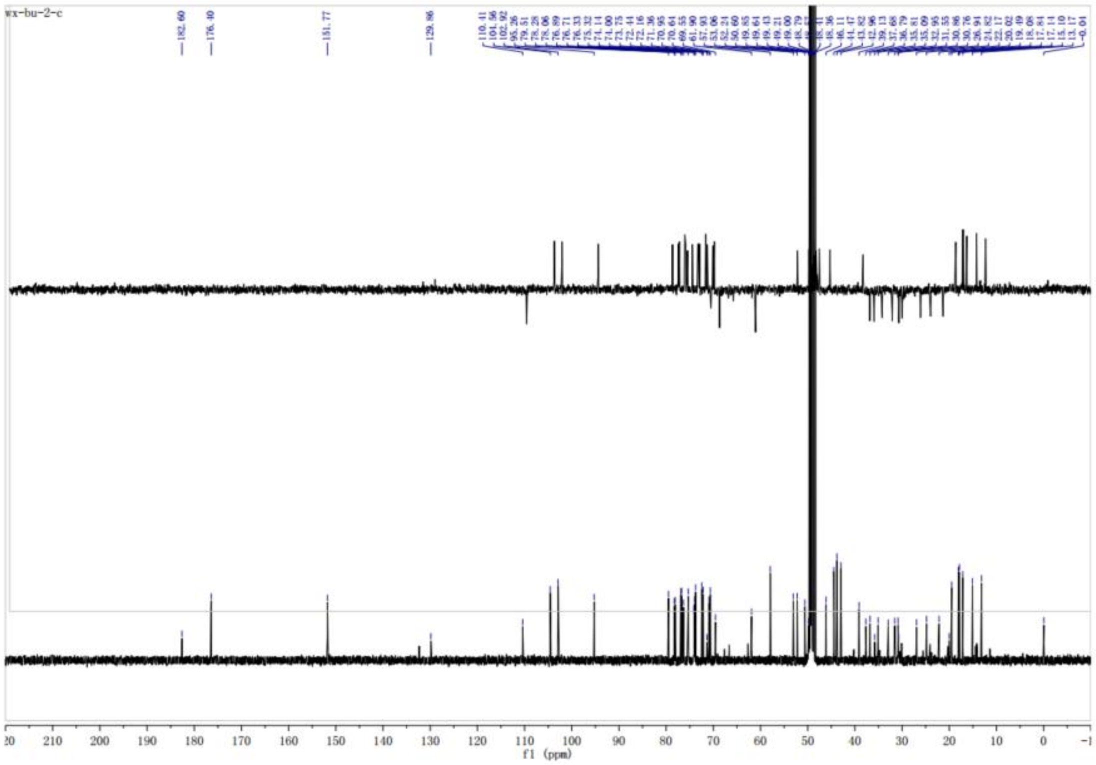

Figure S29. ^{13}C NMR spectrum of acangraciliside S in methanol-d_4

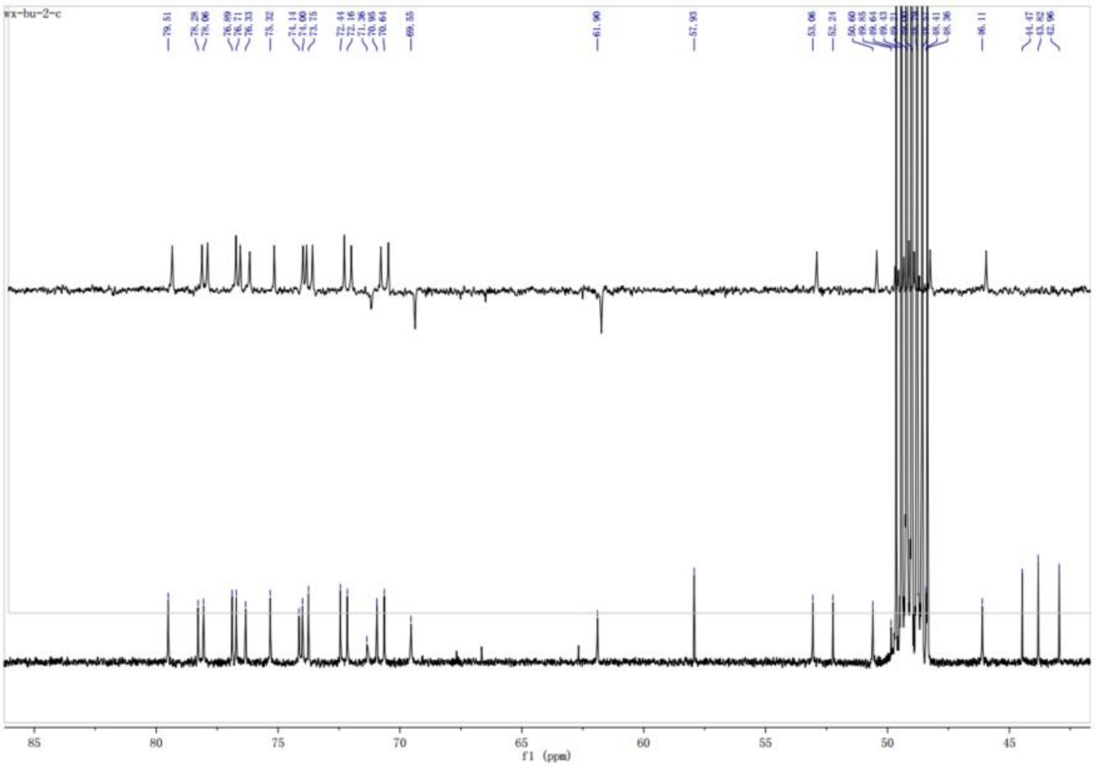

Figure S30. Expand ^{13}C NMR spectrum of acangraciliside S in methanol-d_4

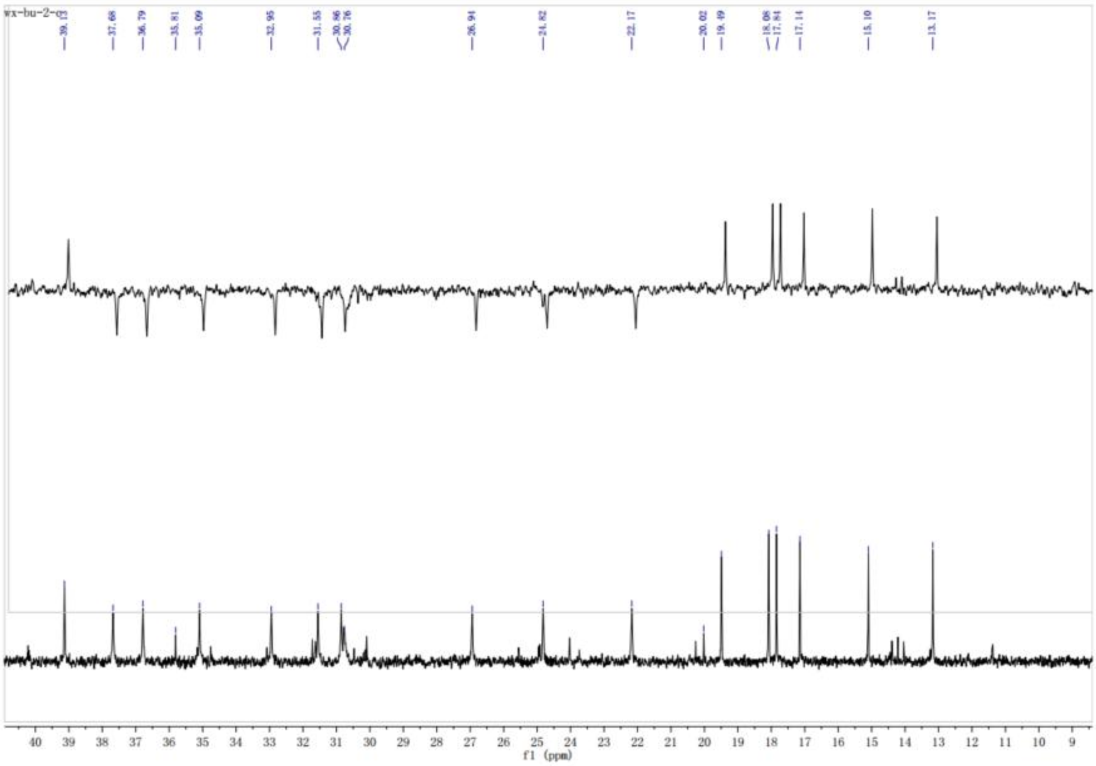

Figure S31. Expand ^{13}C NMR spectrum of acangraciliside S in methanol-d_4

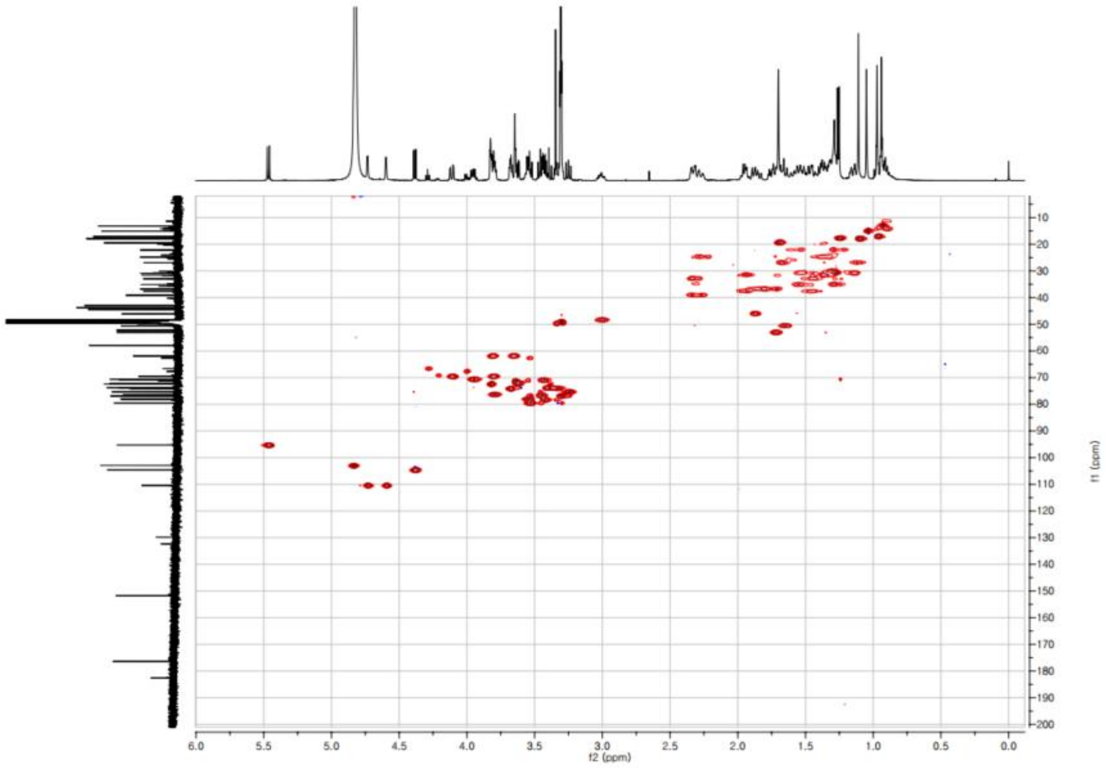

Figure S32. HSQC spectrum of acangraciliside S in methanol-d_4

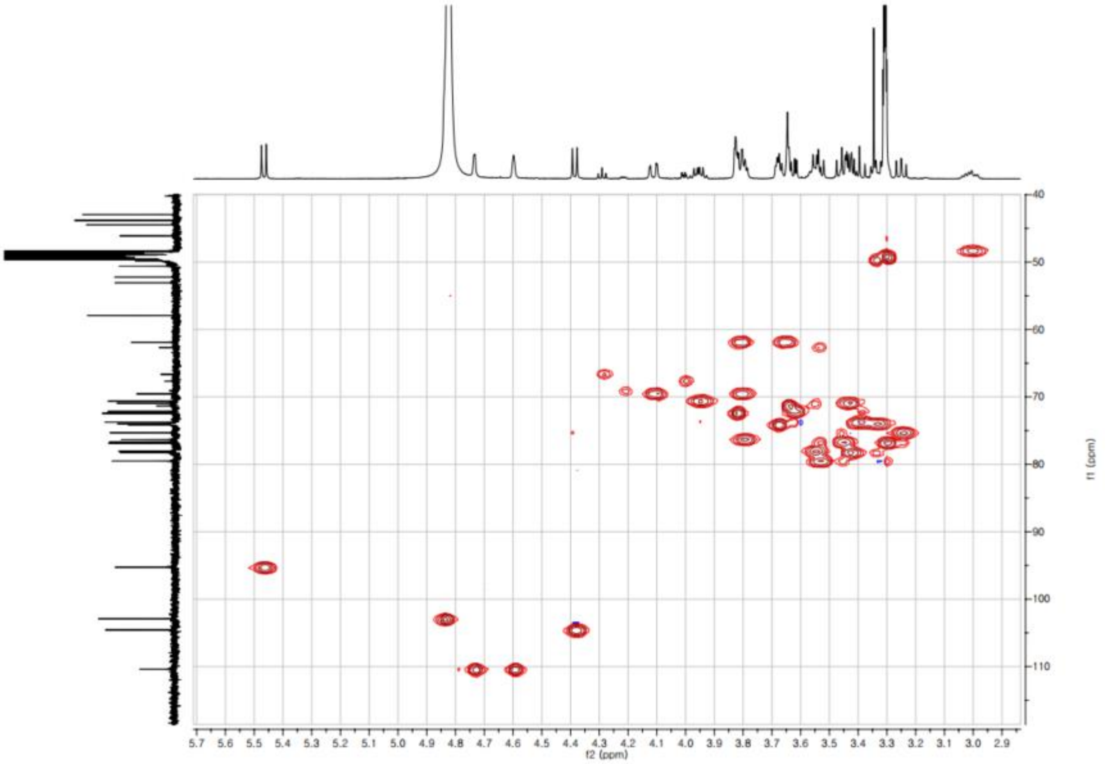

Figure S33. Expand HSQC spectrum of acangraciliside S in methanol-d_4

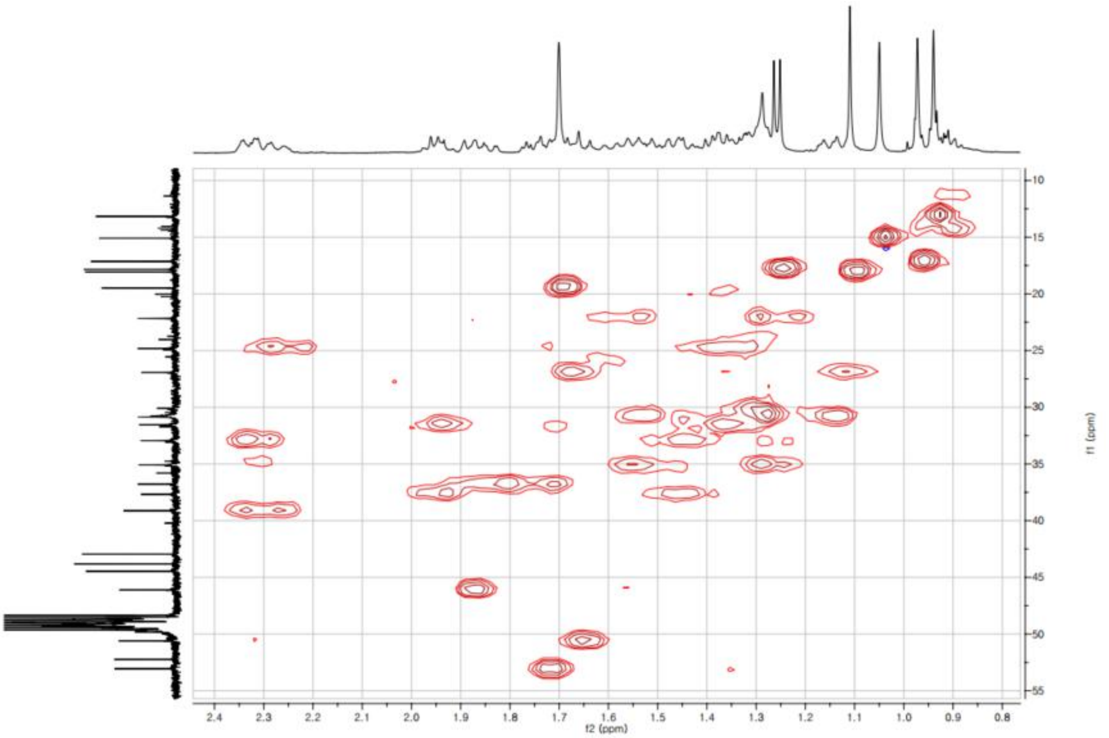

Figure S34. Expand HSQC spectrum of acangraciliside S in methanol-d_4

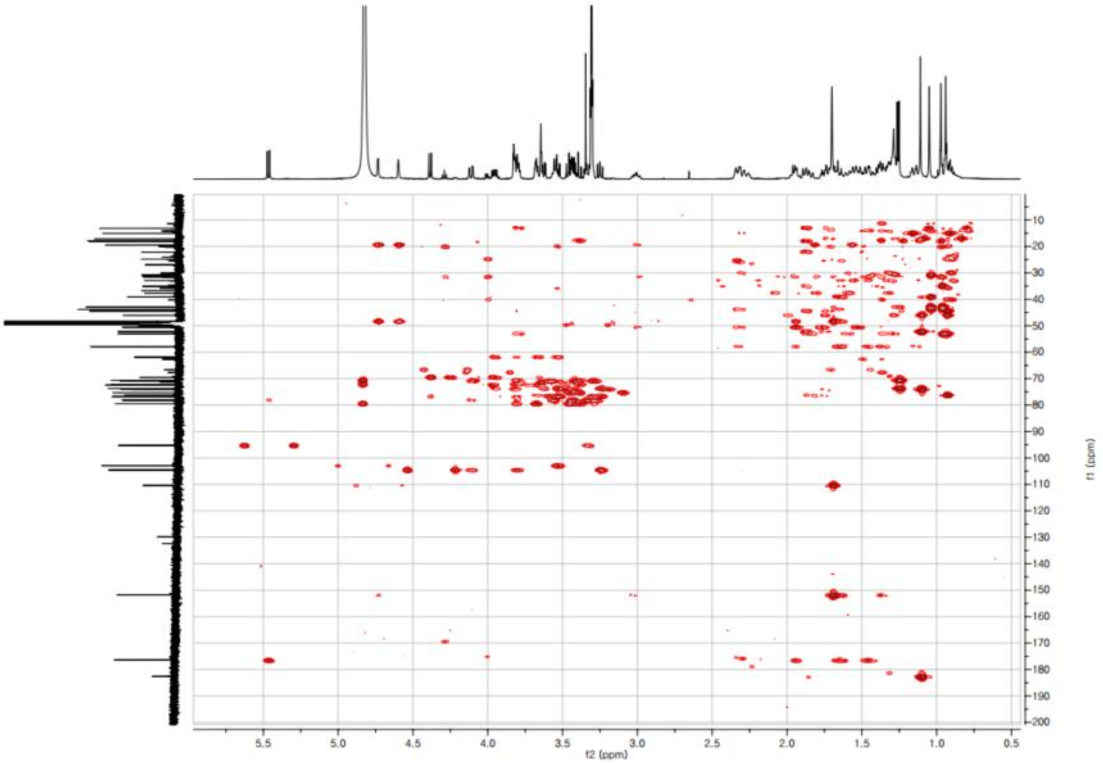

Figure S35. HMBC spectrum of acangraciliside S in methanol-d_4

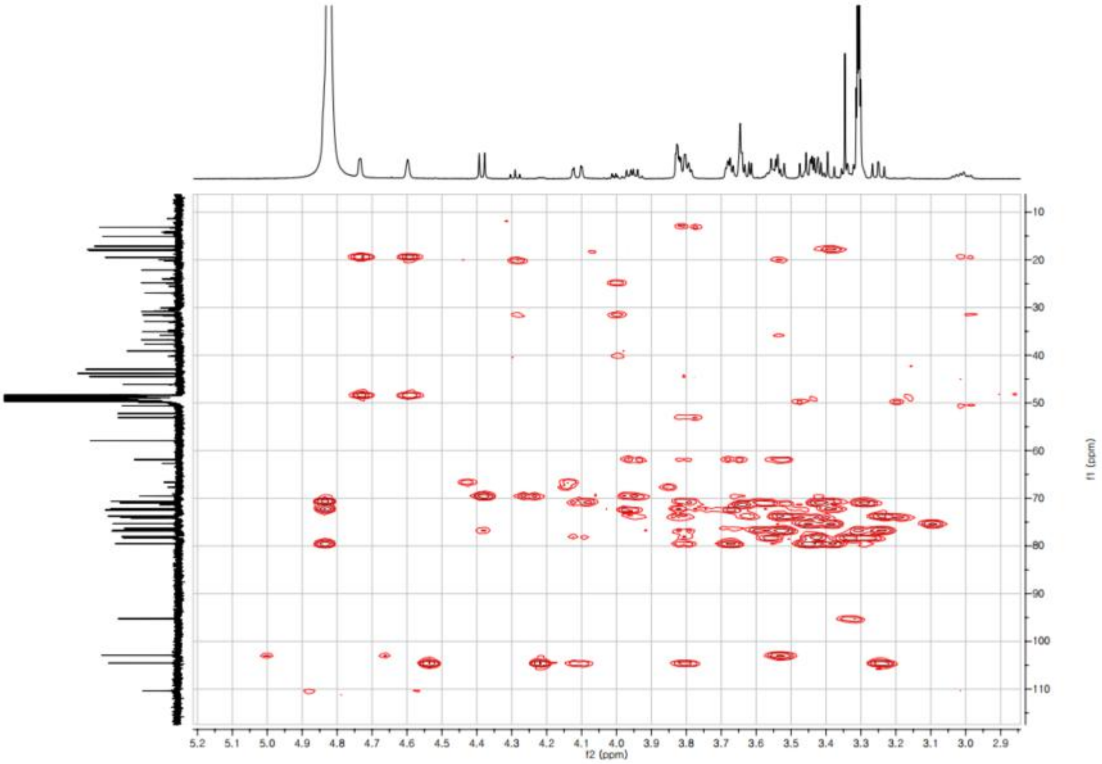

Figure S36. Expand HMBC spectrum of acangraciliside S in methanol-d_4

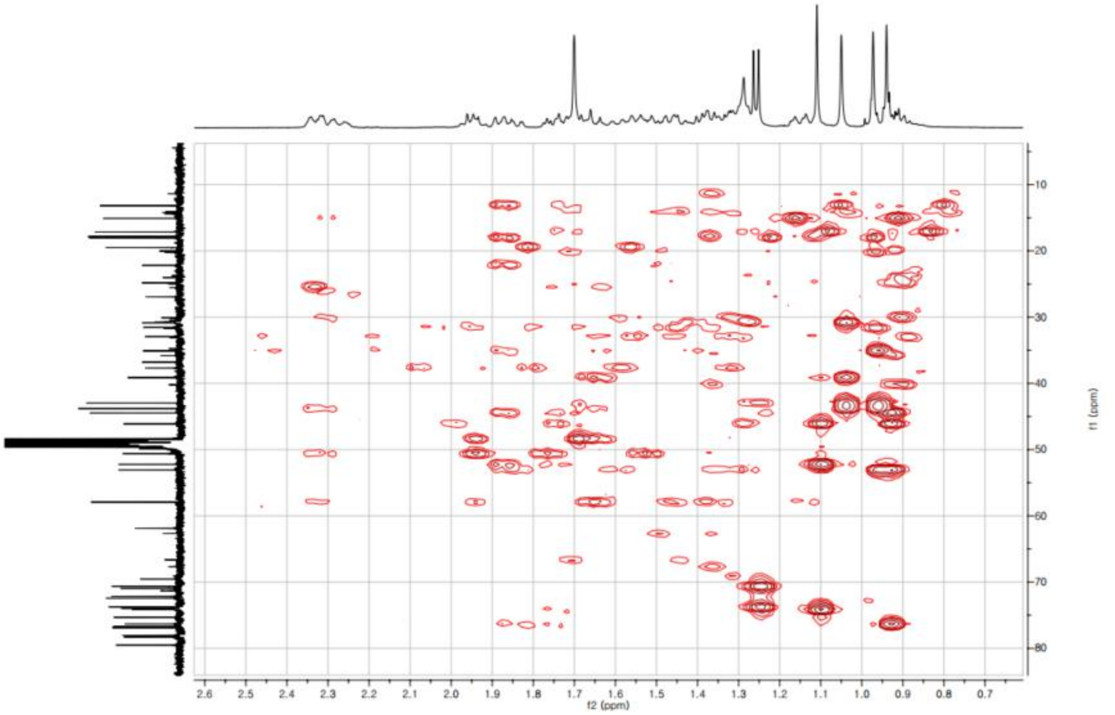

Figure S37. Expand HMBC spectrum of acangraciliside S in methanol-d_4

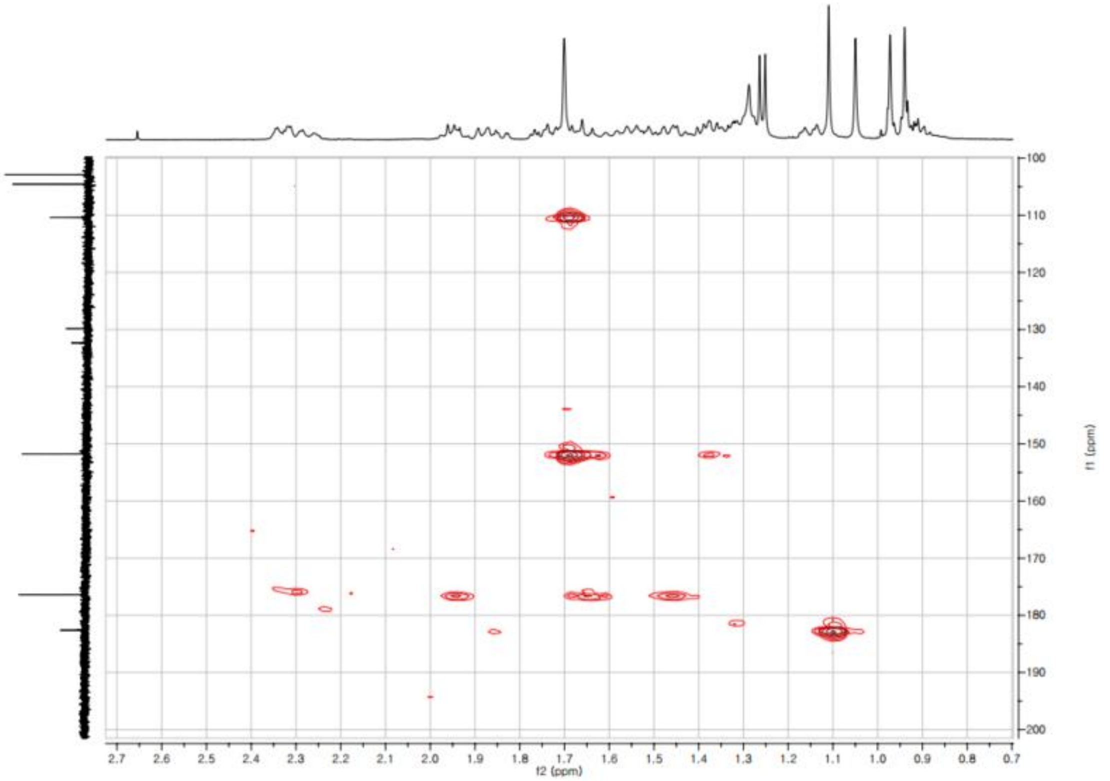

Figure S38. Expand HMBC spectrum of acangraciliside S in methanol-d_4

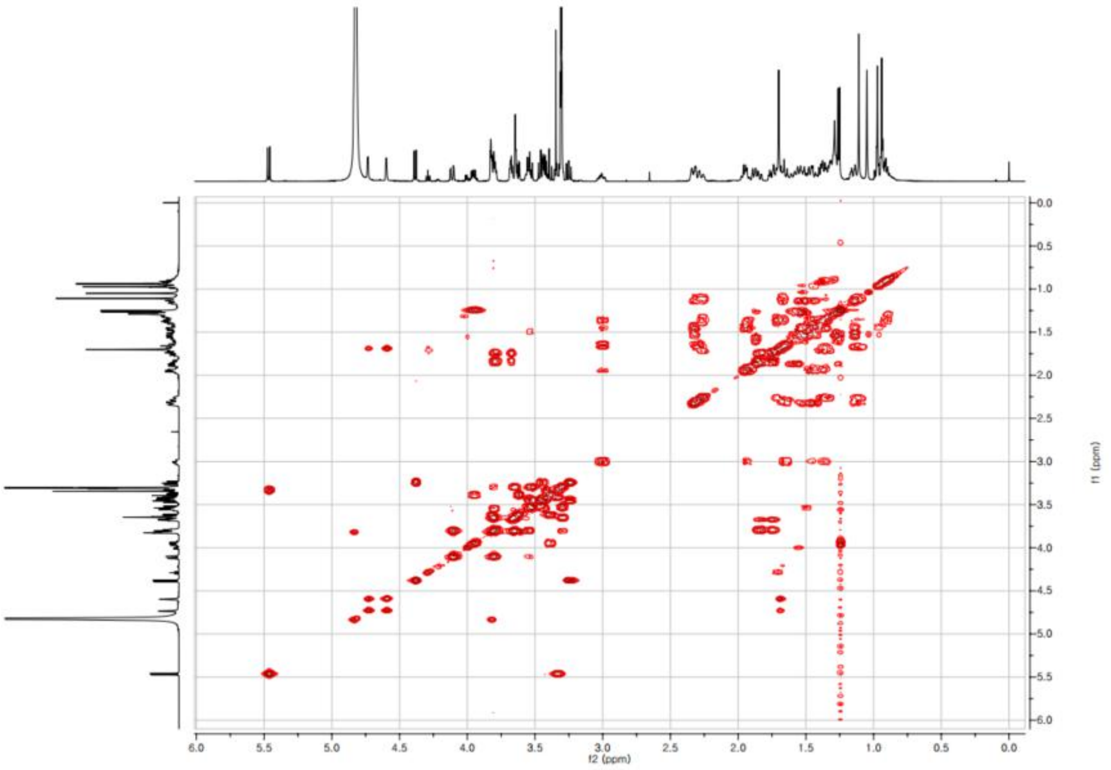

Figure S39. ^{1}H-^{1}H COSY spectrum of acangraciliside S in methanol-d_4

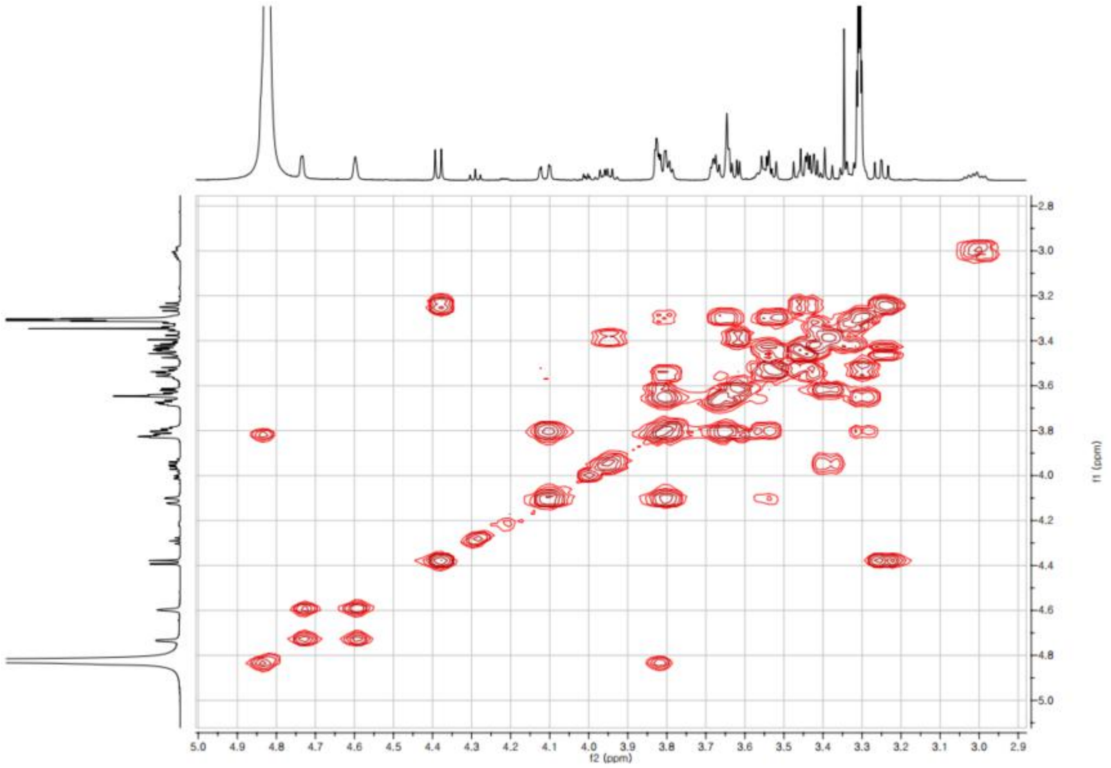

Figure S40. Expand ^{1}H-^{1}H COSY spectrum of acangraciliside S in methanol-d_4

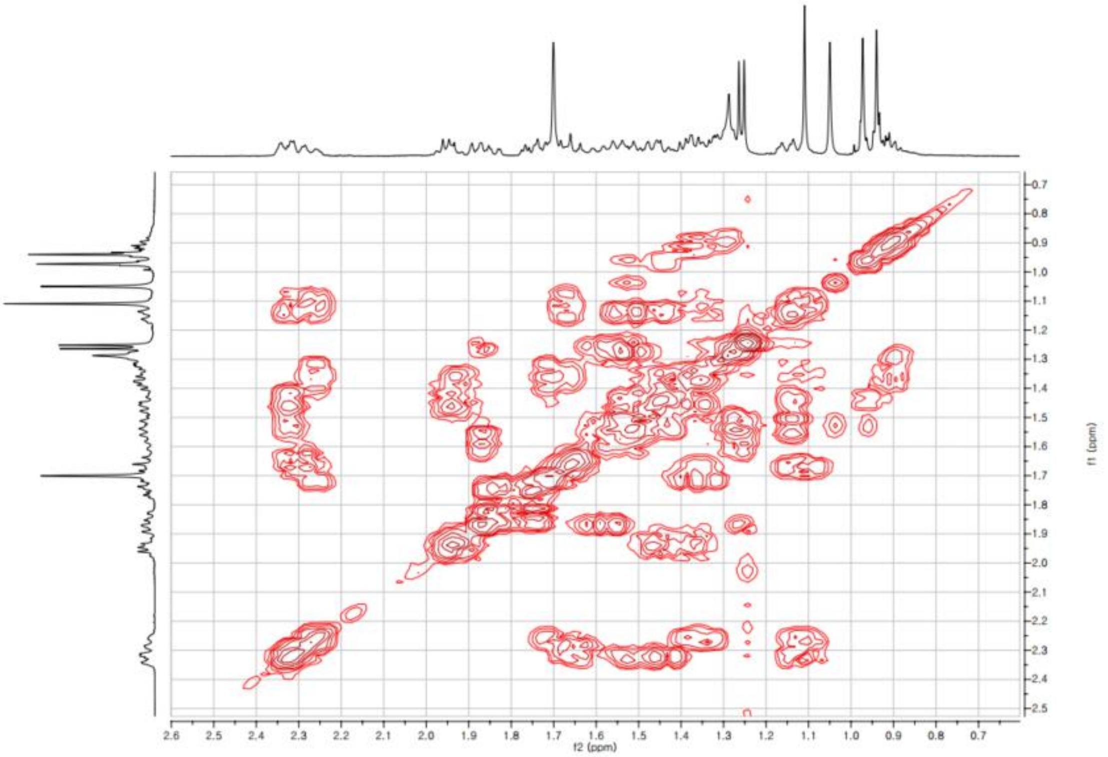

Figure S41. Expand ^1H-^1H COSY spectrum of acangracilisde S in methanol-d_4

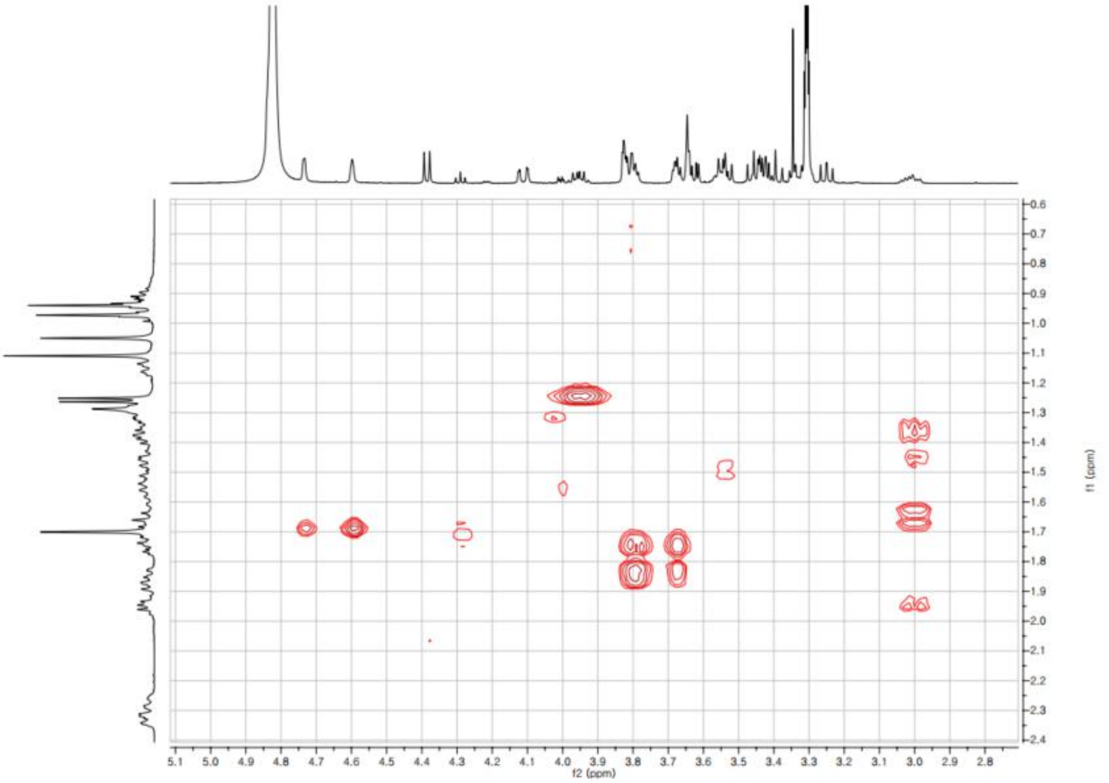

Figure S42. Expand ^1H-^1H COSY spectrum of acangraciliside S in methanol-d_4

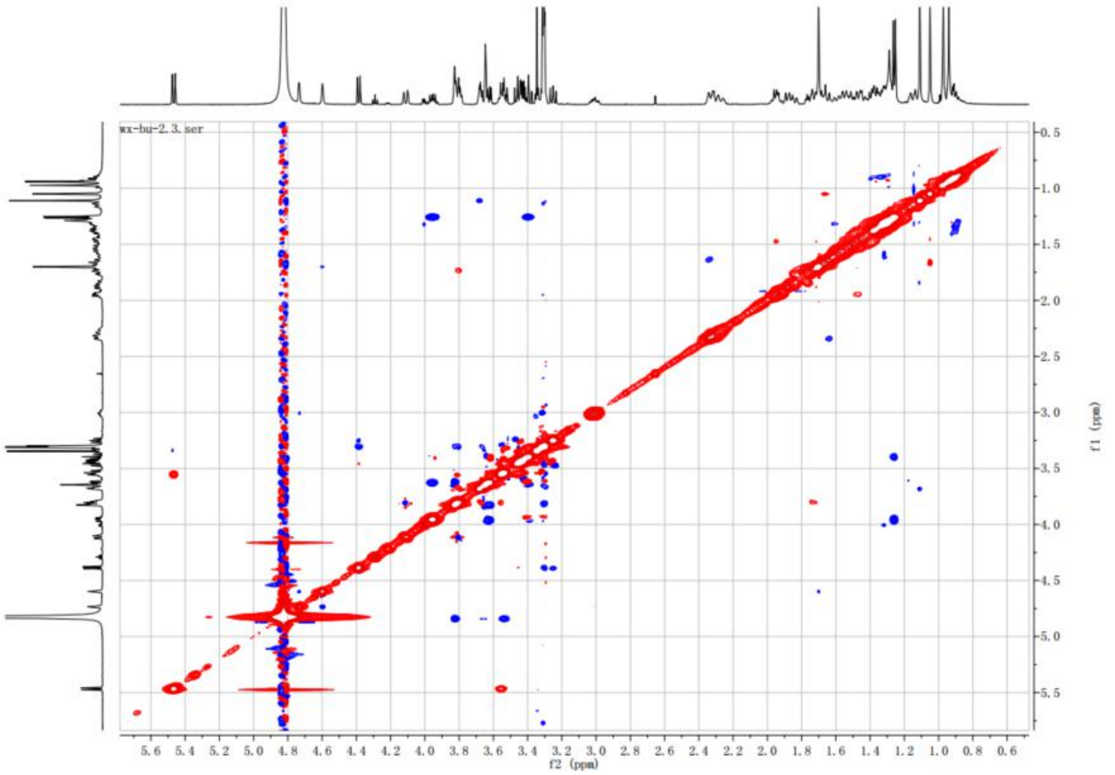

Figure S43. NOESY spectrum of acangraciliside S in methanol-d_4

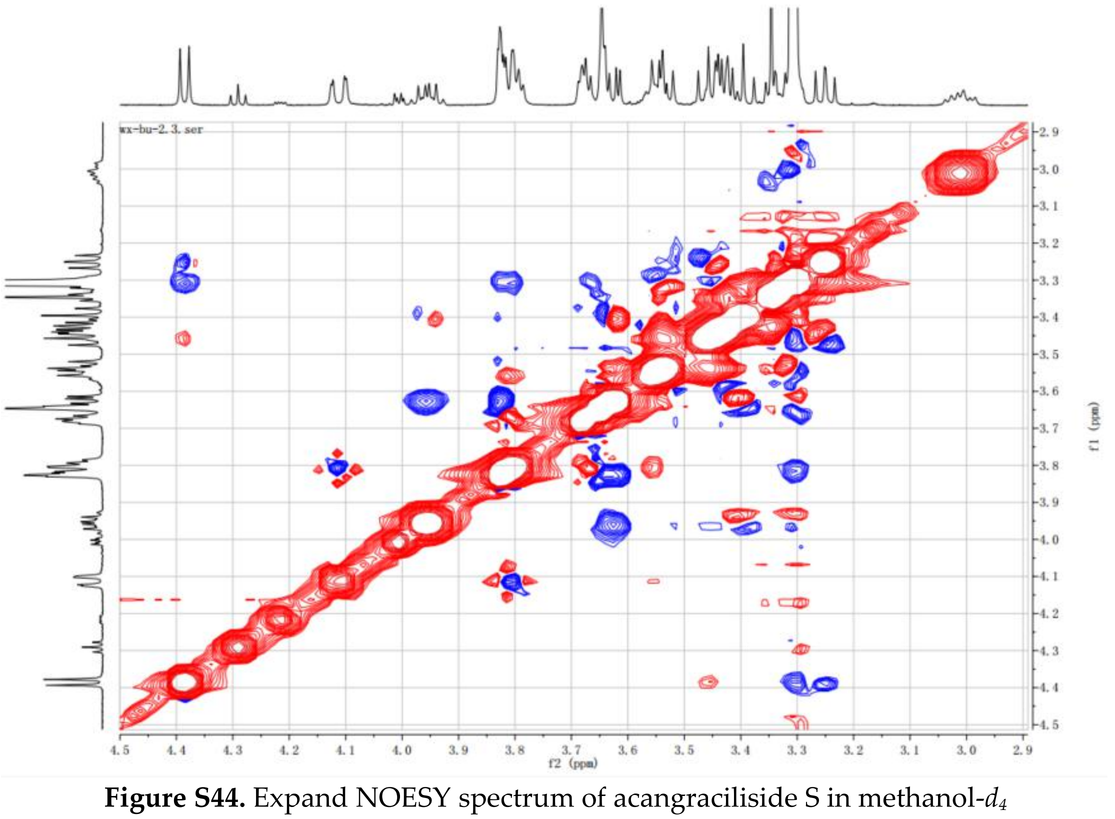

Figure S44. Expand NOESY spectrum of acangraciliside S in methanol-d_4

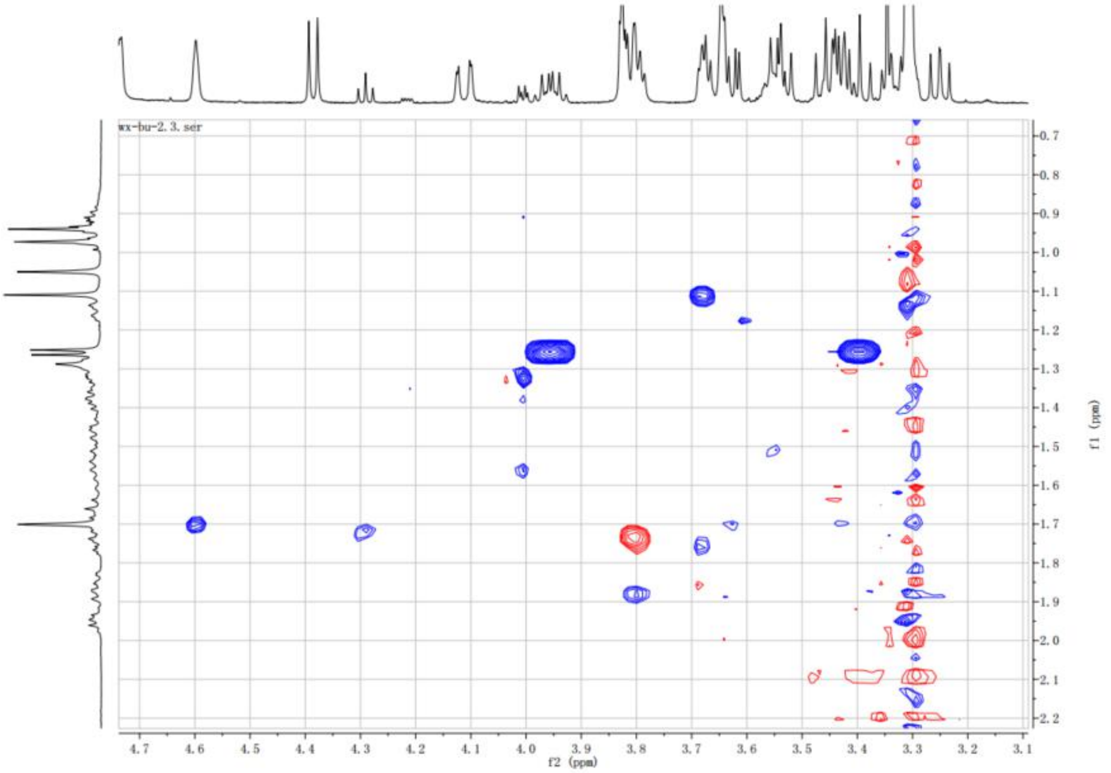

Figure S45. Expand NOESY spectrum of acangraciliside S in methanol-d_4

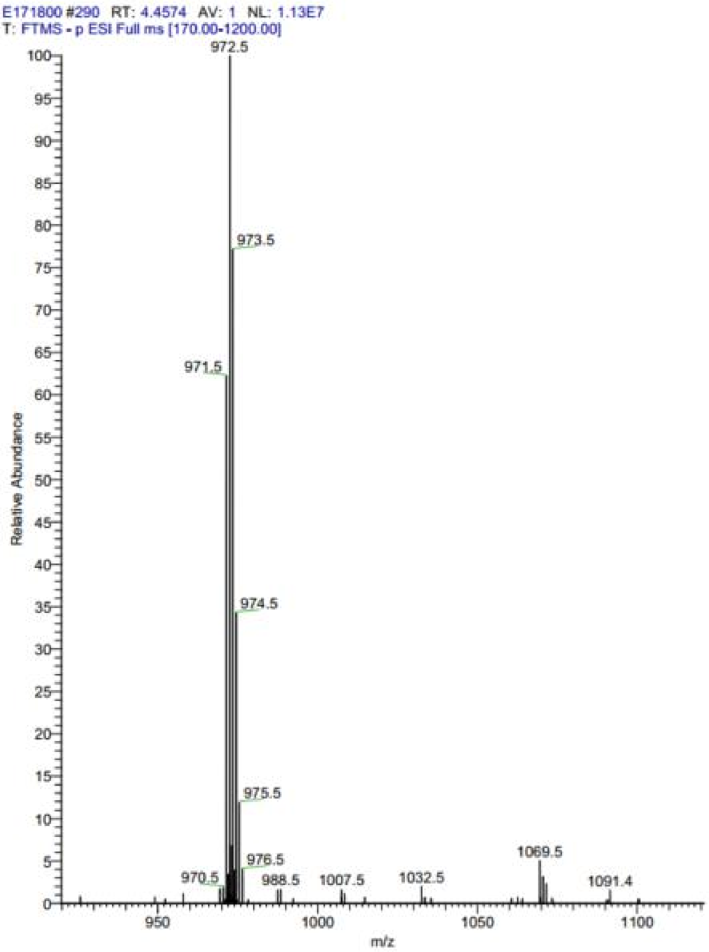

Figure S46. ESIMS spectrum of acangraciliside S

National Center for Organic Mass Spectrometry in Shanghai
Shanghai Institute of Organic Chemistry
Chinese Academic of Sciences
High Resolution MS DATA REPORT

Instrument: Thermo Fisher Scientific LTQ FTICR

Card Serial Number : E171800

Sample Serial Number: 1

Operator : ZHUFJ Date: 2017/05/09

Operation Mode: ESI Negative

Elemental composition search on mass 971.49

m/z= 966.49-976.49

m/z	Theo. Mass	Delta (ppm)	RDB equiv.	Composition
971.4865	971.4857	0.84	11.5	$C_{48}H_{75}O_{20}$
	971.4884	-1.92	16.0	$C_{51}H_{73}O_{17}N$
	971.4911	-4.68	20.5	$C_{54}H_{71}O_{14}N_2$

Figure S47. HRESIMS spectrum of acangraciliside S

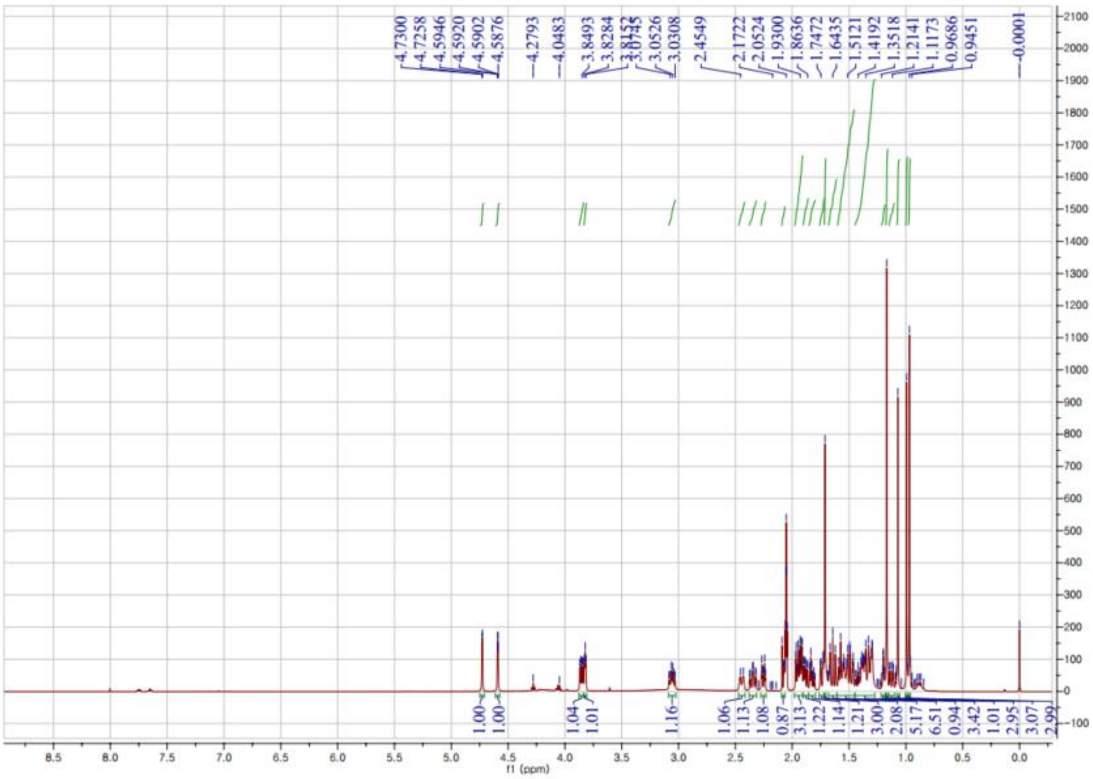

Figure S48. ^{1}H NMR spectrum of **2a** in acetone-d_6

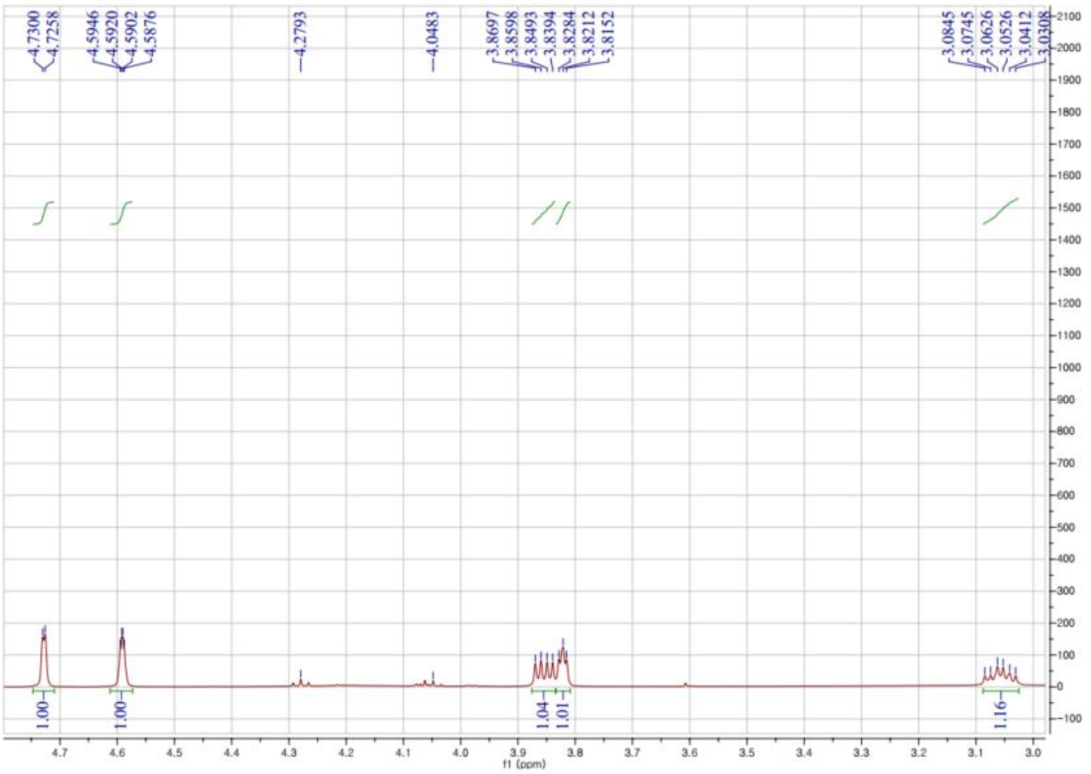

Figure S49. Expand ^{1}H NMR spectrum of **2a** in acetone-d_6

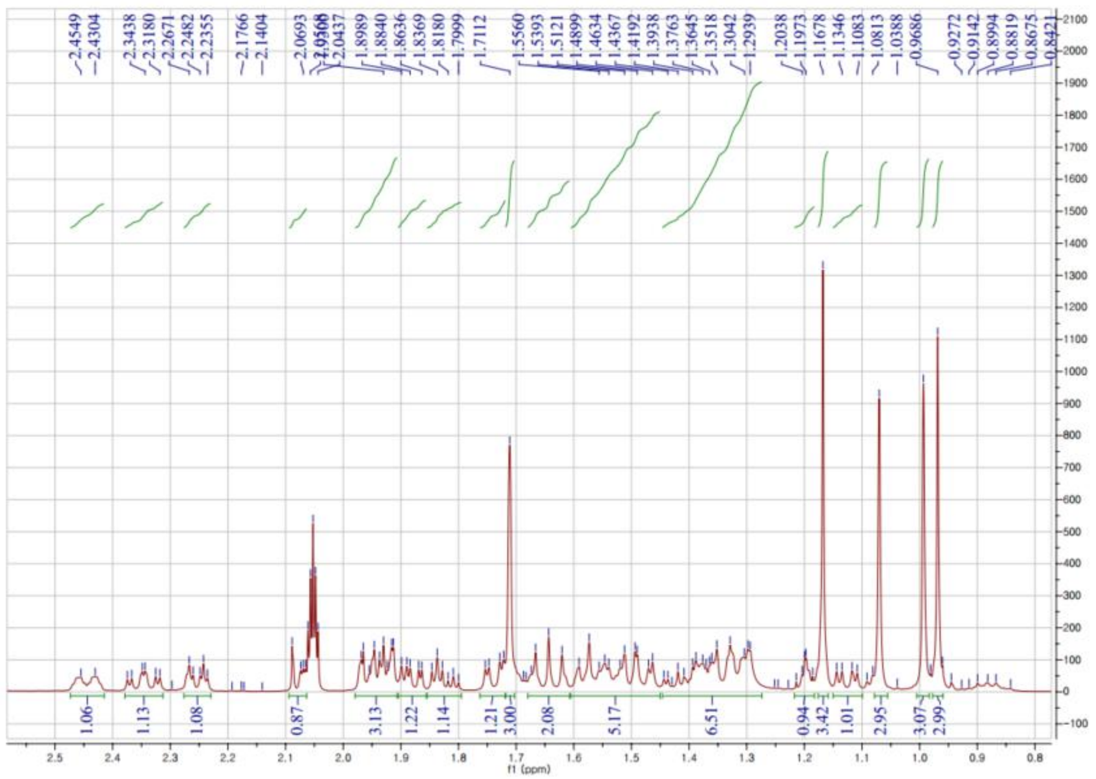

Figure S50. Expand ^{1}H NMR spectrum of **2a** in acetone-d_6

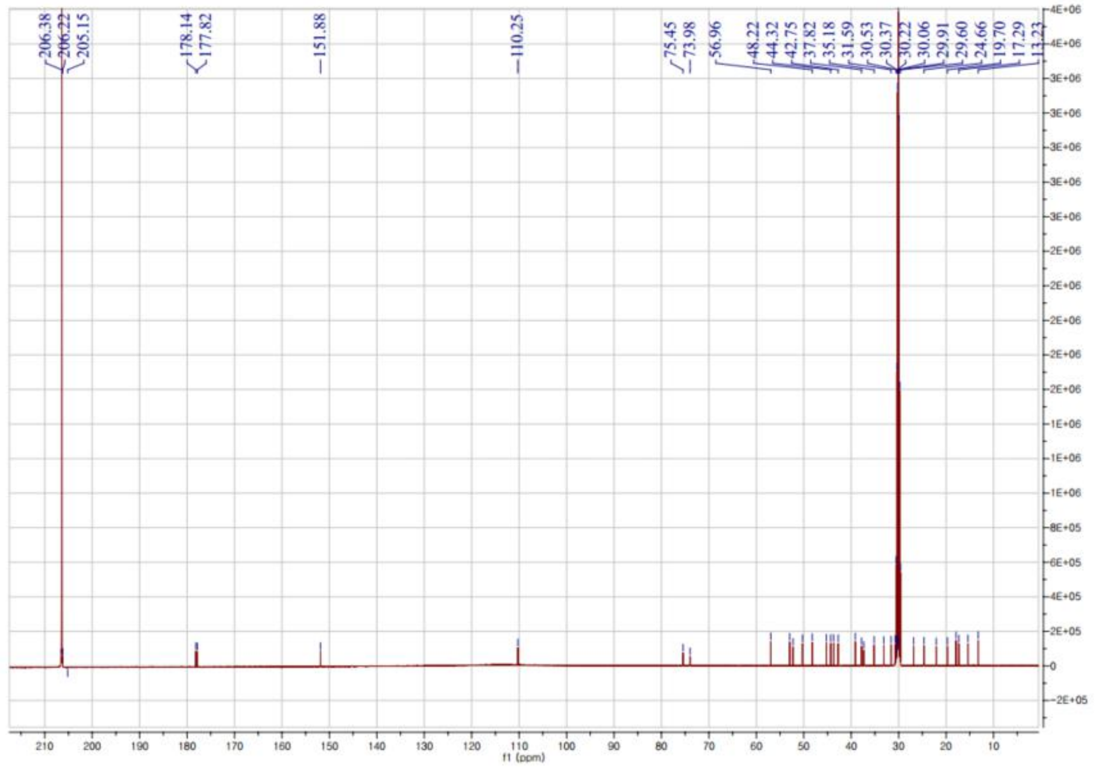

Figure S51. ^{13}C NMR spectrum of **2a** in acetone-d_6

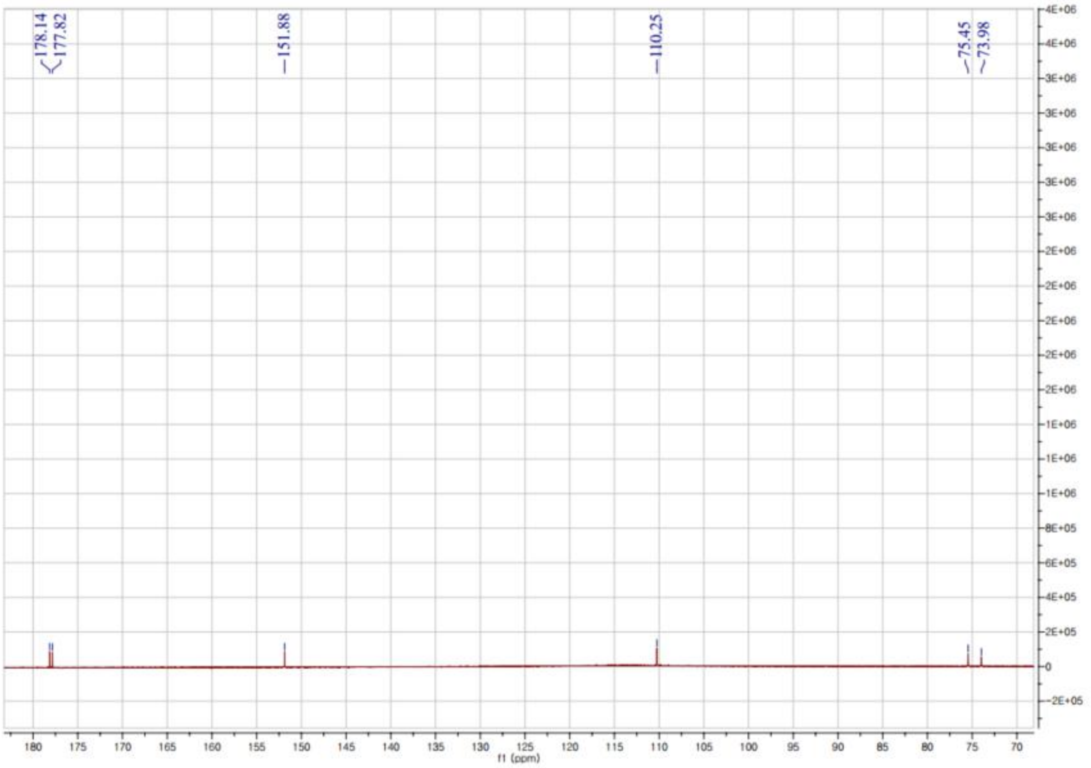

Figure S52. Expand ^{13}C NMR spectrum of **2a** in acetone-d_6

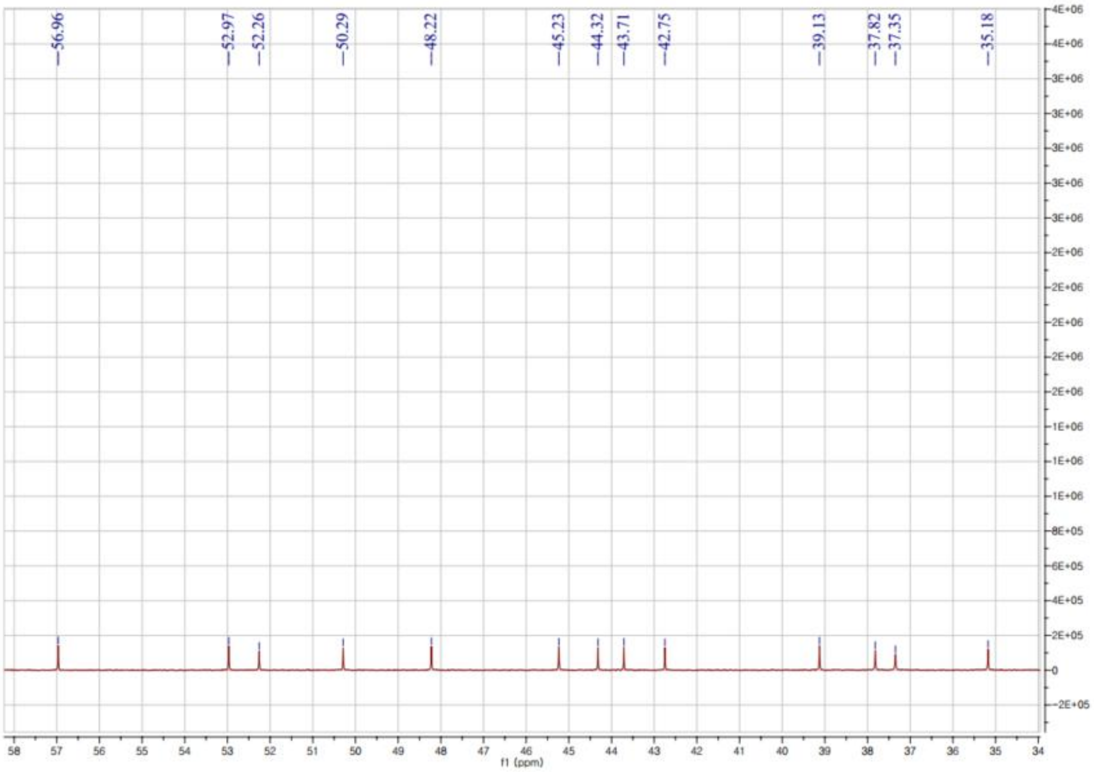

Figure S53. Expand ^{13}C NMR spectrum of **2a** in acetone-d_6

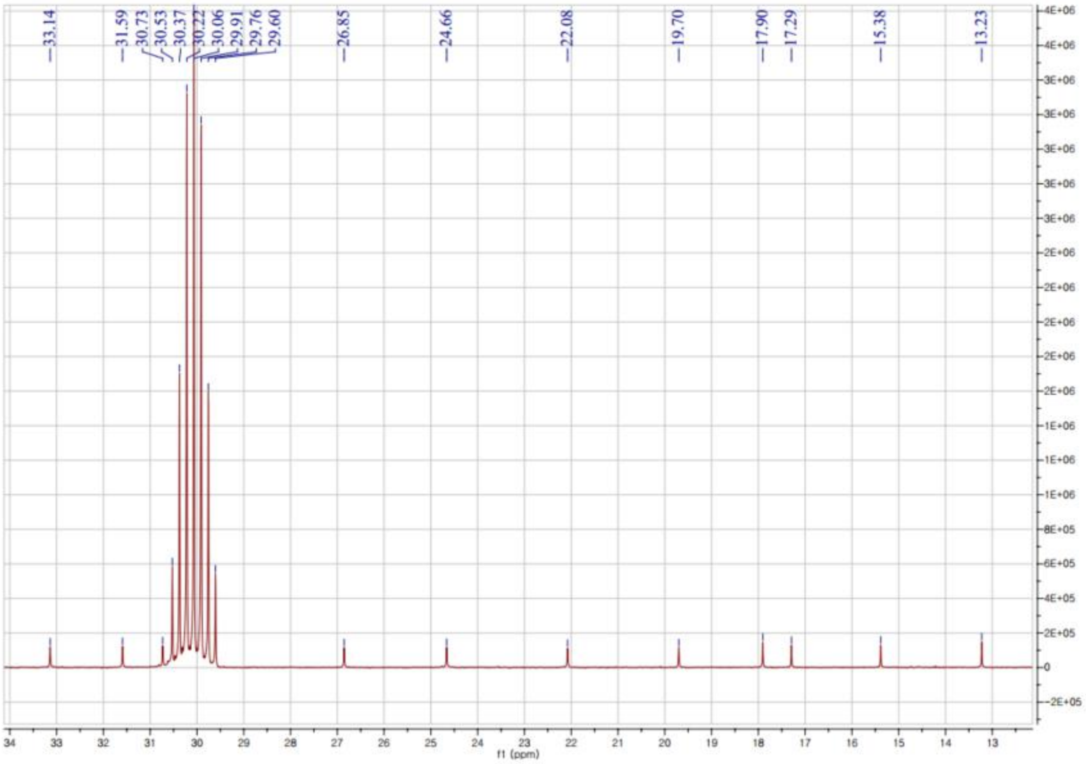

Figure S54. Expand ^{13}C NMR spectrum of **2a** in acetone-d_6

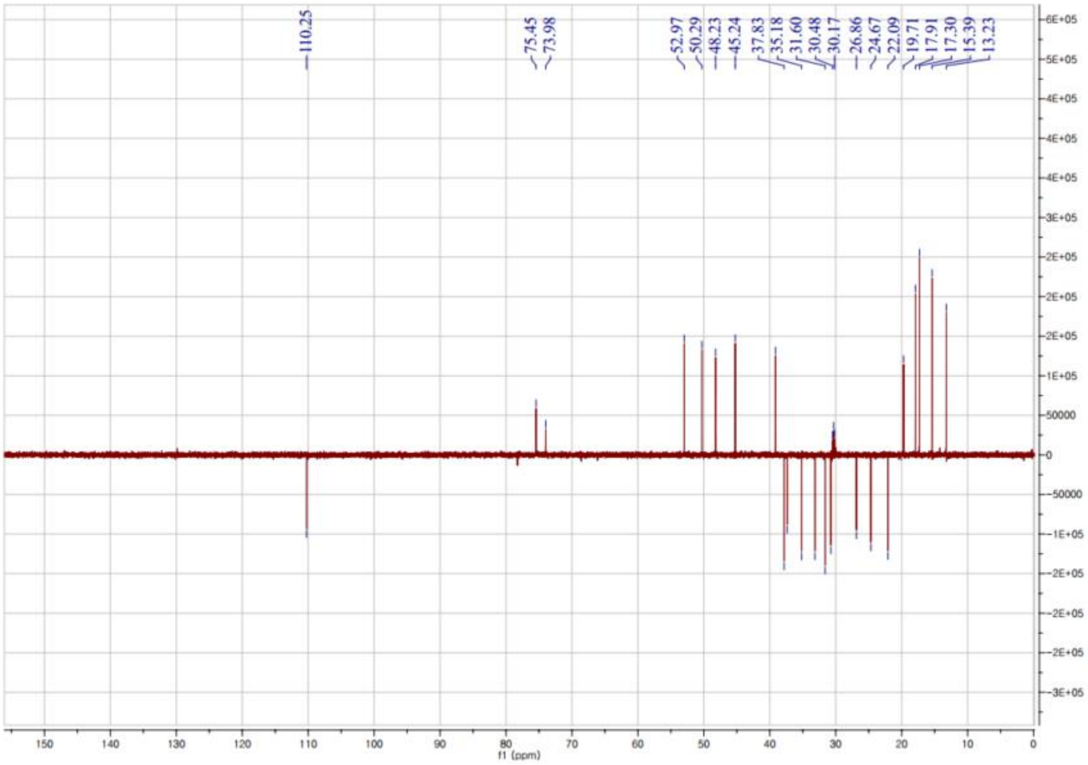

Figure S55. DEPT spectrum of **2a** in acetone-d_6

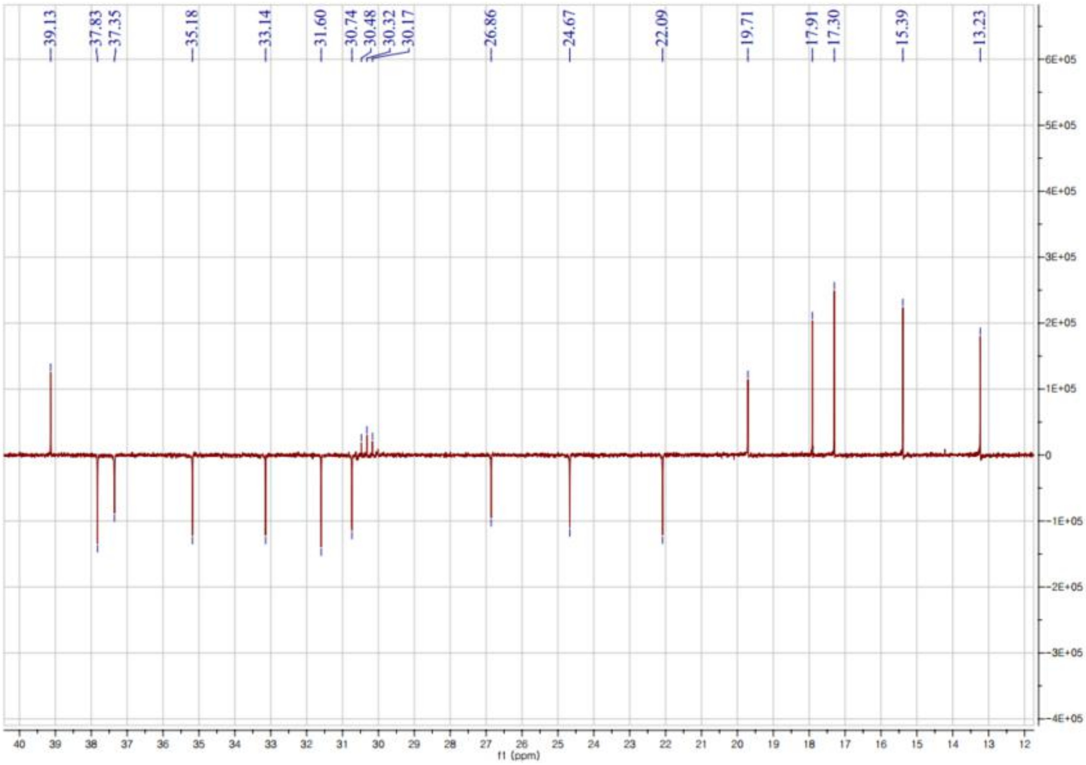

Figure S56. Expand DEPT spectrum of **2a** in acetone-d_6

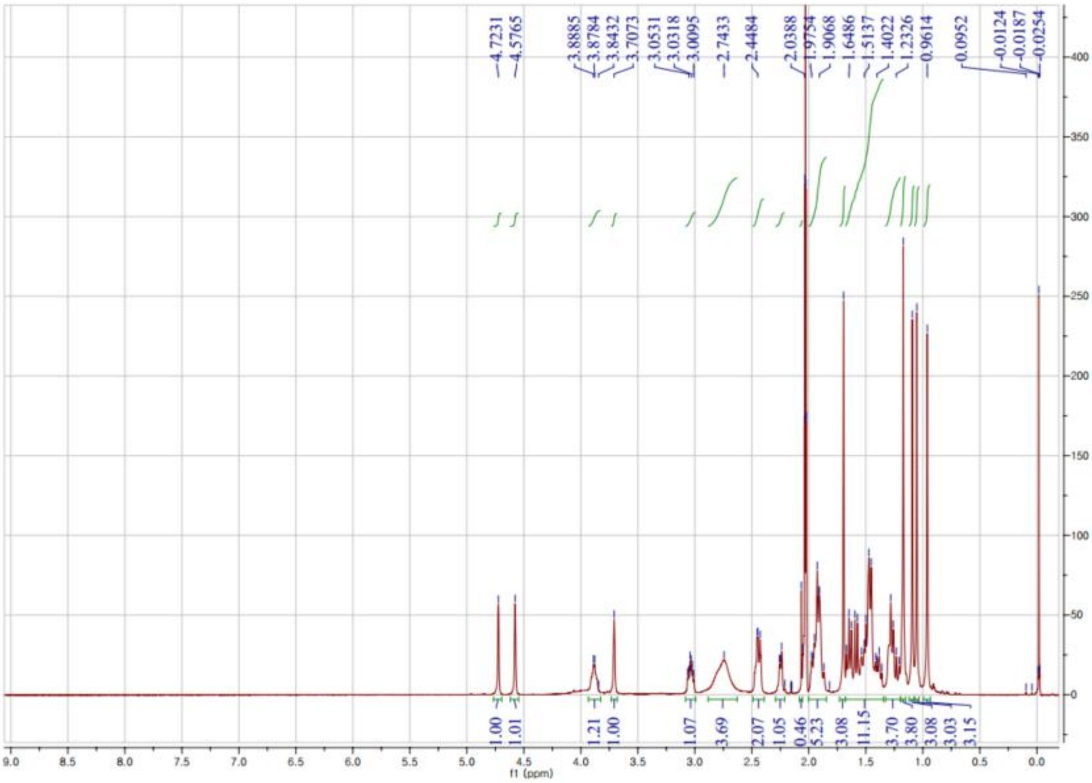

Figure S57. ^{1}H NMR spectrum of 3α,11α-dihydroxy-lup-20(29)-en-23,28-dioic acid in acetone-d_6

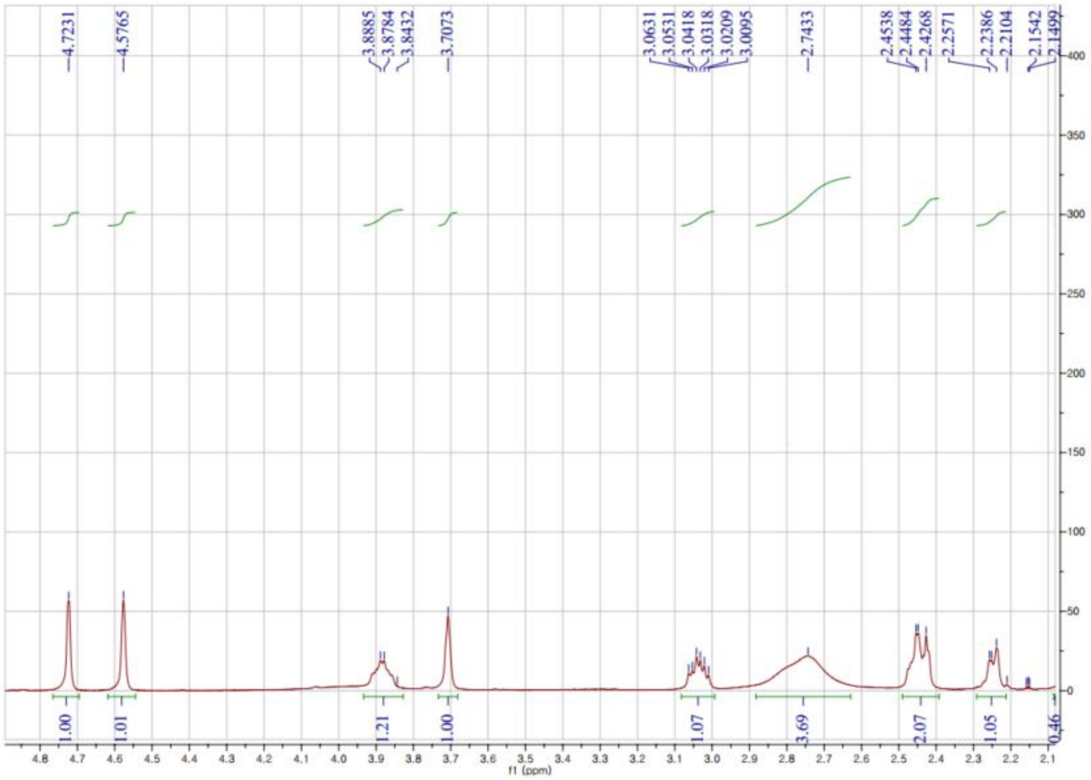

Figure S58. Expand ^{1}H NMR spectrum of 3α,11α-dihydroxy-lup-20(29)-en-23,28-dioic acid in acetone-d_6

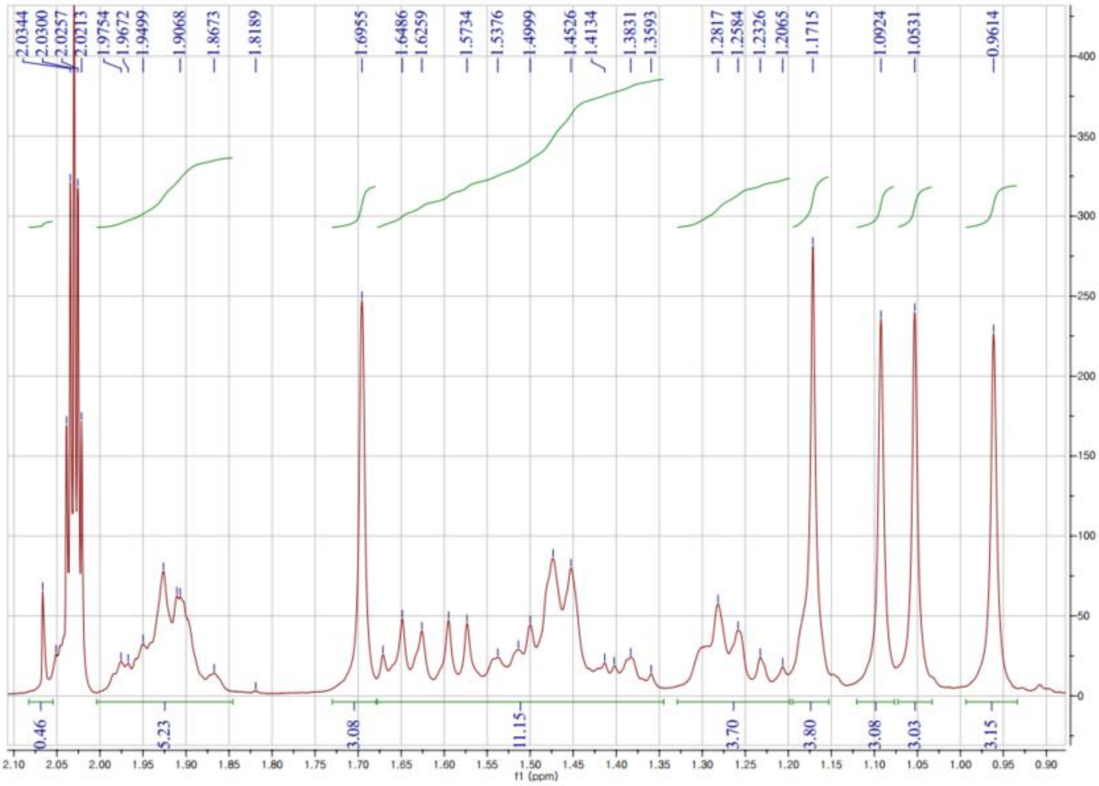

Figure S59. Expand ^{1}H NMR spectrum of 3α,11α-dihydroxy-lup-20(29)-en-23,28-dioic acid in acetone-d_6

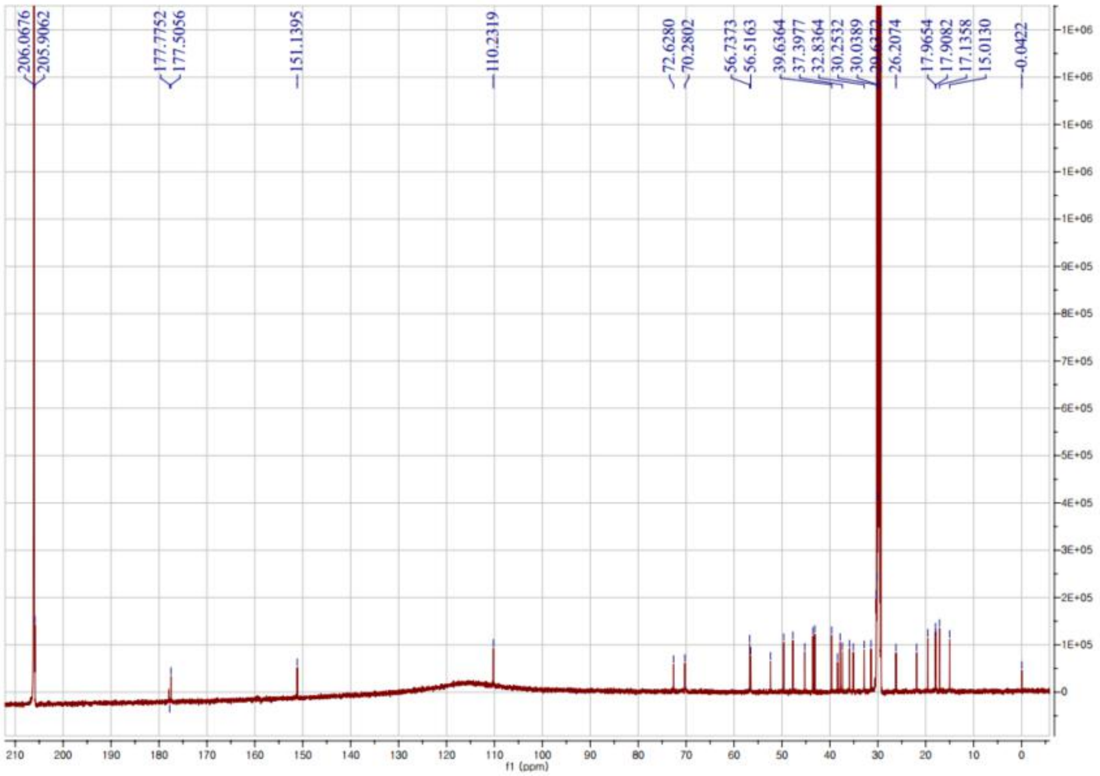

Figure S60. ^{13}C NMR spectrum of 3α,11α-dihydroxy-lup-20(29)-en-23,28-dioic acid in acetone-d_6

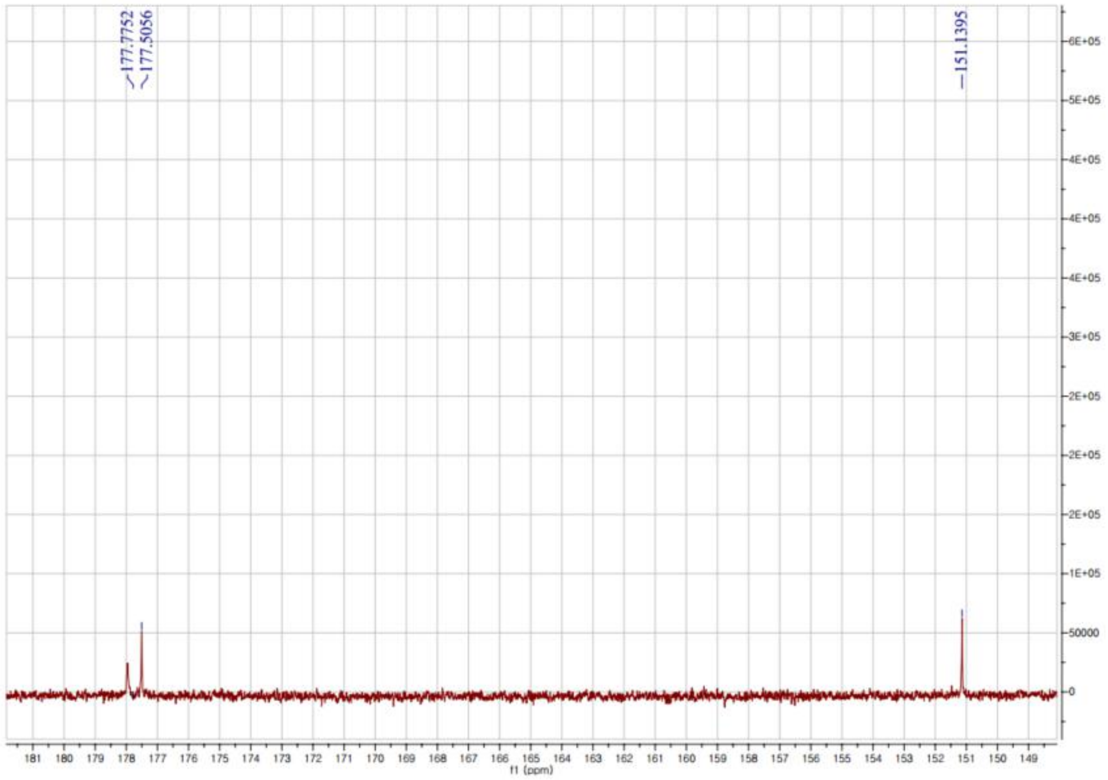

Figure S61. Expand ^{13}C NMR spectrum of 3α,11α-dihydroxy-lup-20(29)-en-23,28-dioic acid in acetone-d_6

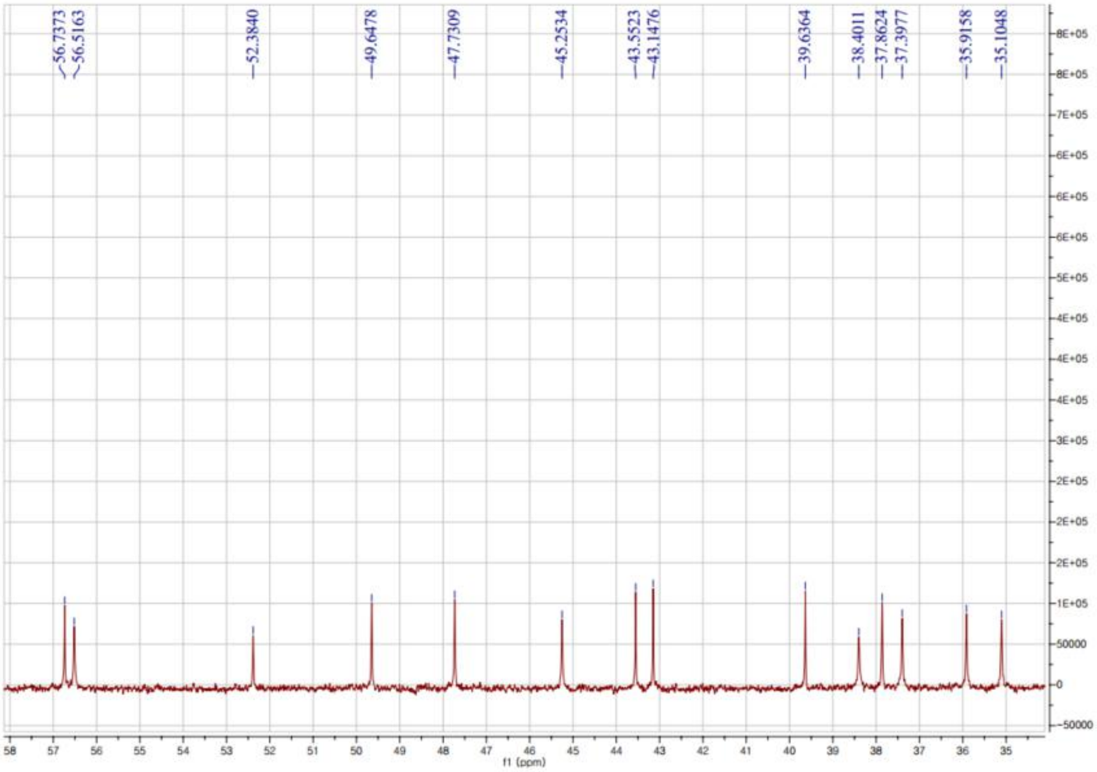

Figure S62. Expand ^{13}C NMR spectrum of 3α,11α-dihydroxy-lup-20(29)-en-23,28-dioic acid in acetone-d_6

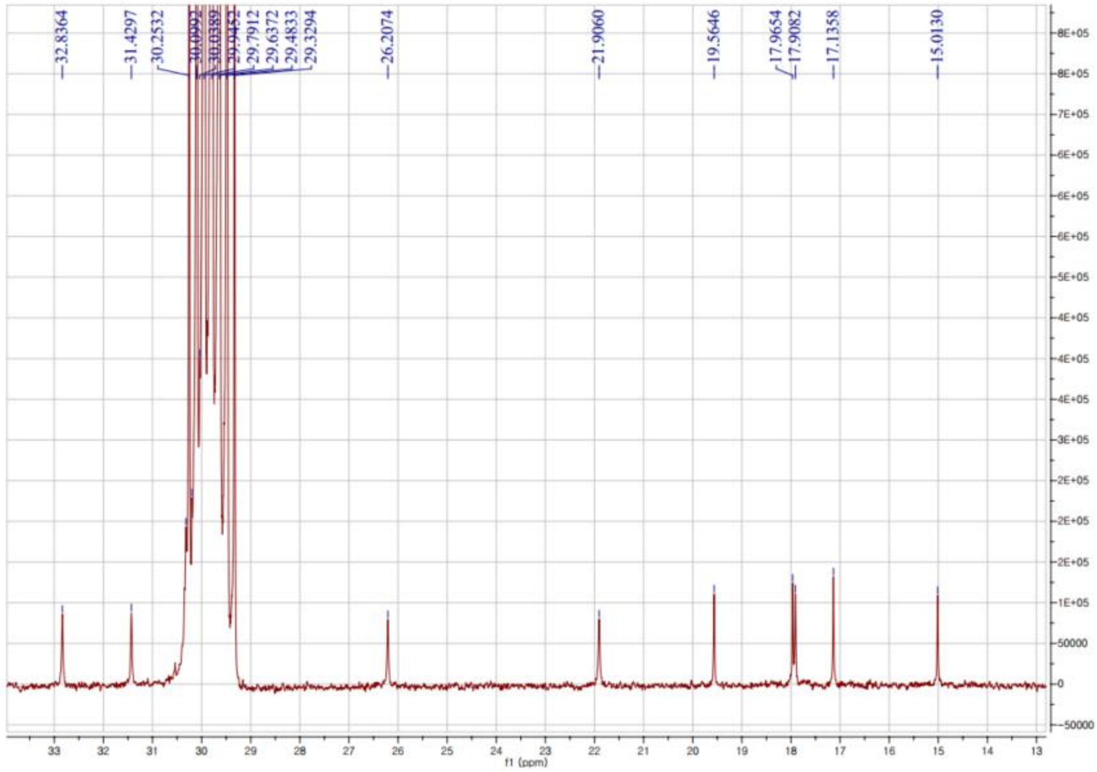

Figure S63. Expand ^{13}C NMR spectrum of 3α,11α-dihydroxy-lup-20(29)-en-23,28-dioic acid in acetone-d_6

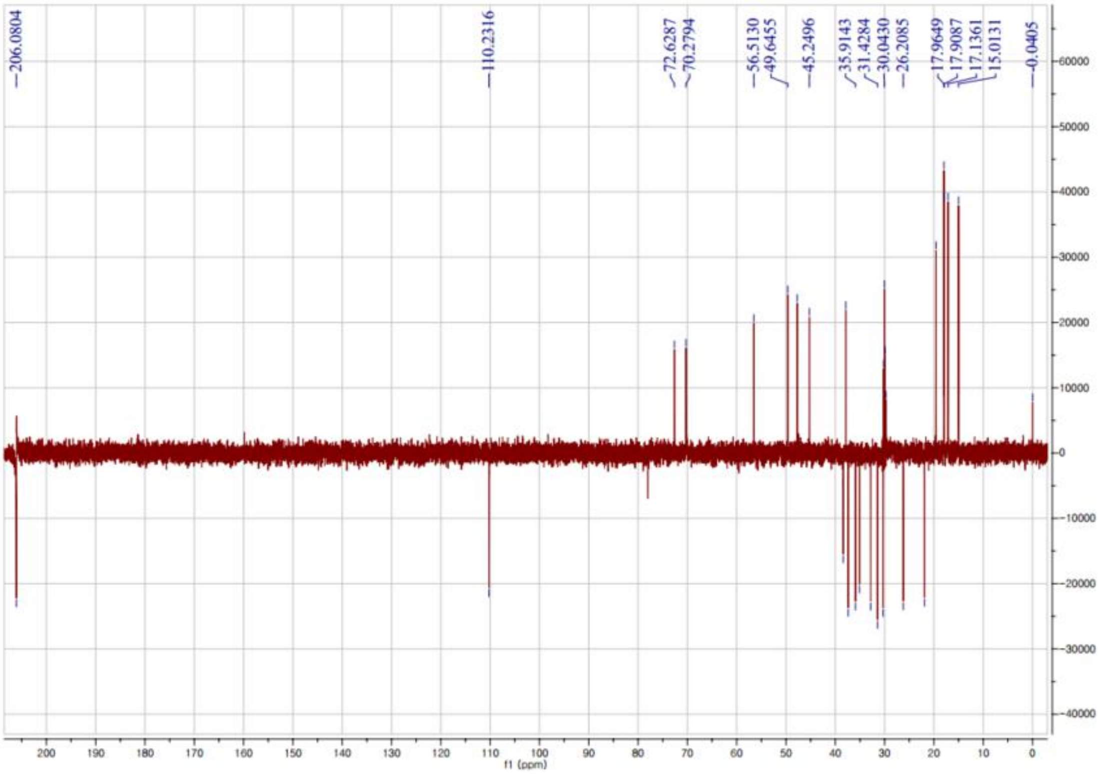

Figure S64. DEPT spectrum of 3α,11α-dihydroxy-lup-20(29)-en-23,28-dioic acid in acetone-d_6

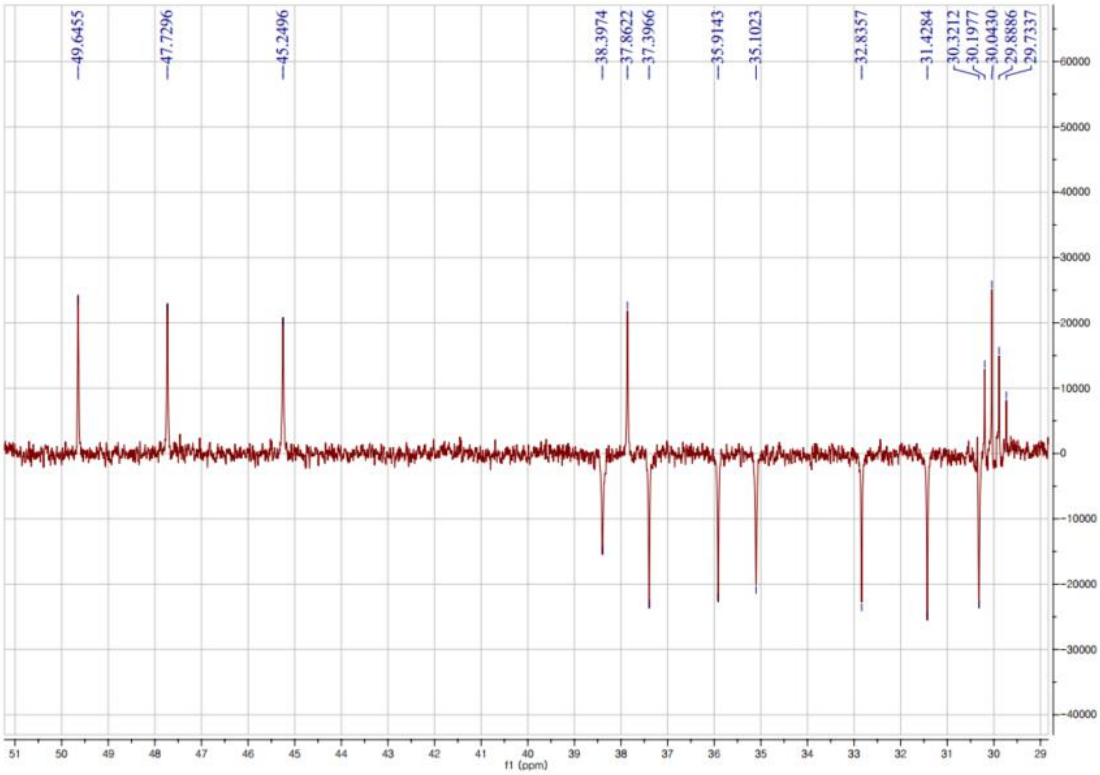

Figure S65. Expand DEPT spectrum of 3α,11α-dihydroxy-lup-20(29)-en-23,28-dioic acid in acetone-d_6

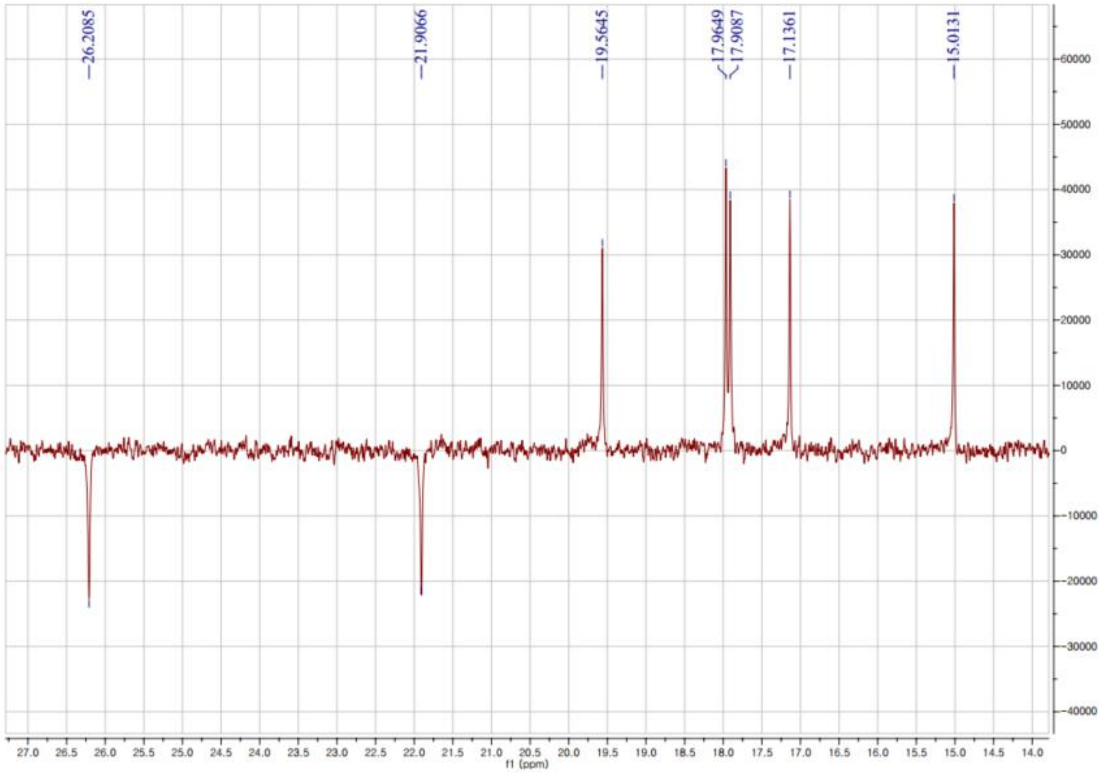

Figure S66. Expand DEPT spectrum of 3α,11α-dihydroxy-lup-20(29)-en-23,28-dioic acid in acetone-d_6

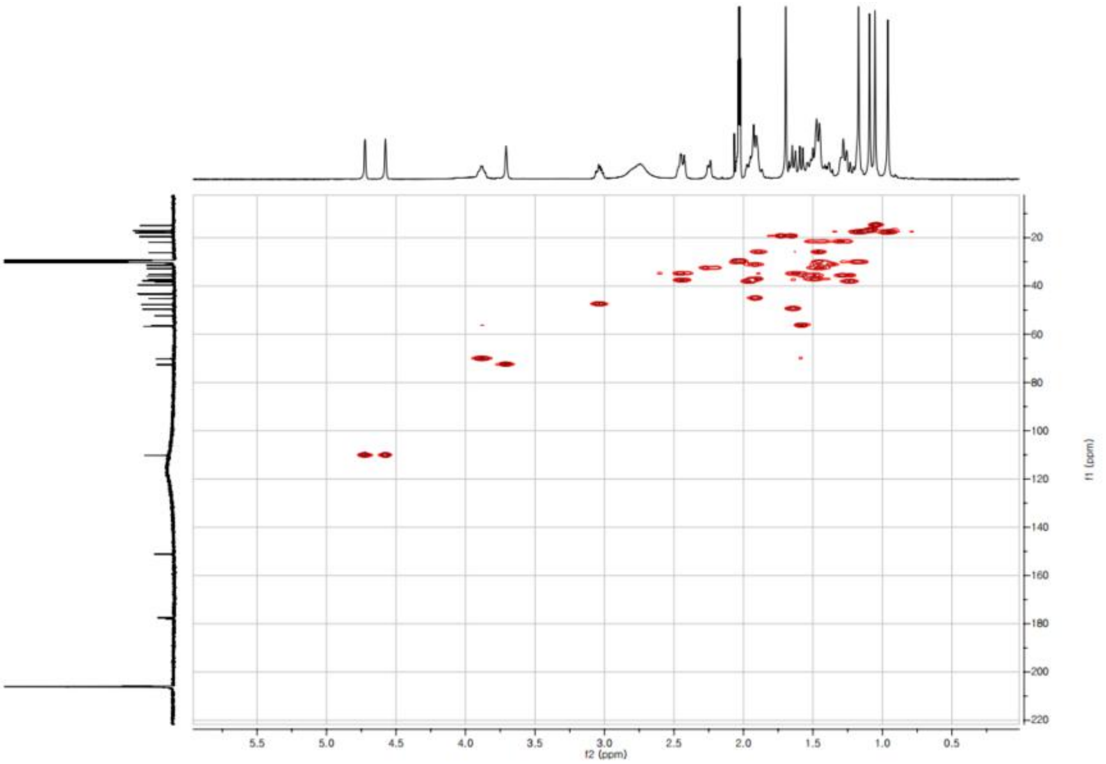

Figure S67. HSQC spectrum of 3α,11α-dihydroxy-lup-20(29)-en-23,28-dioic acid in acetone-d_6

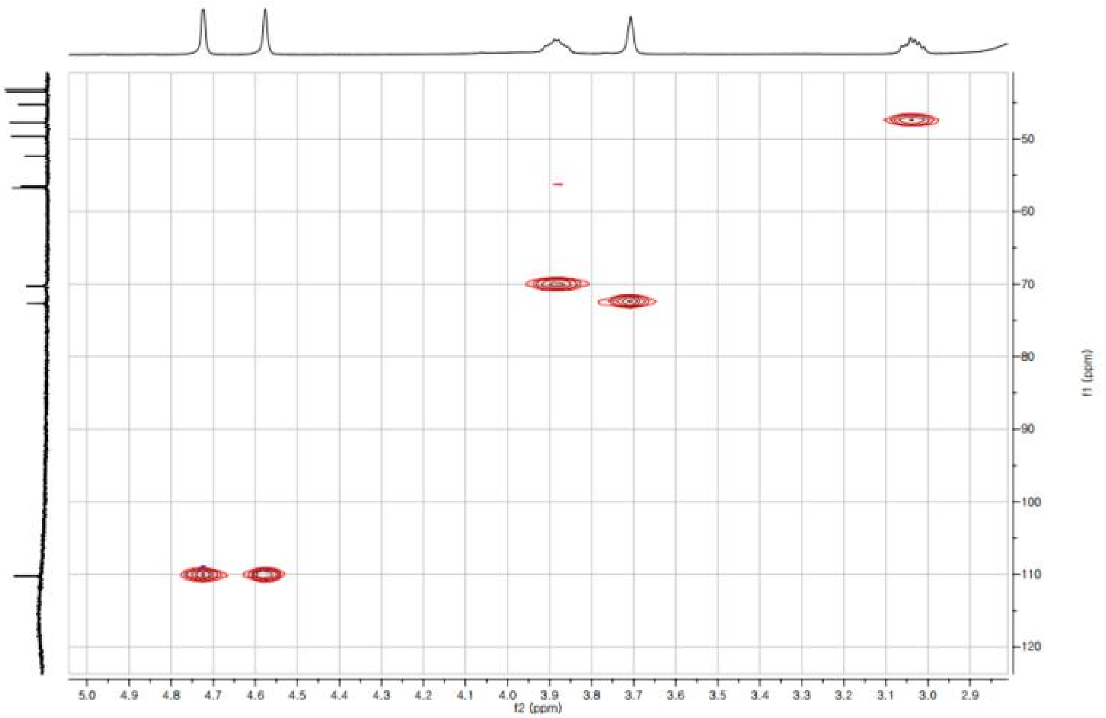

Figure S68. Expand HSQC spectrum of 3α,11α-dihydroxy-lup-20(29)-en-23,28-dioic acid in acetone-d_6

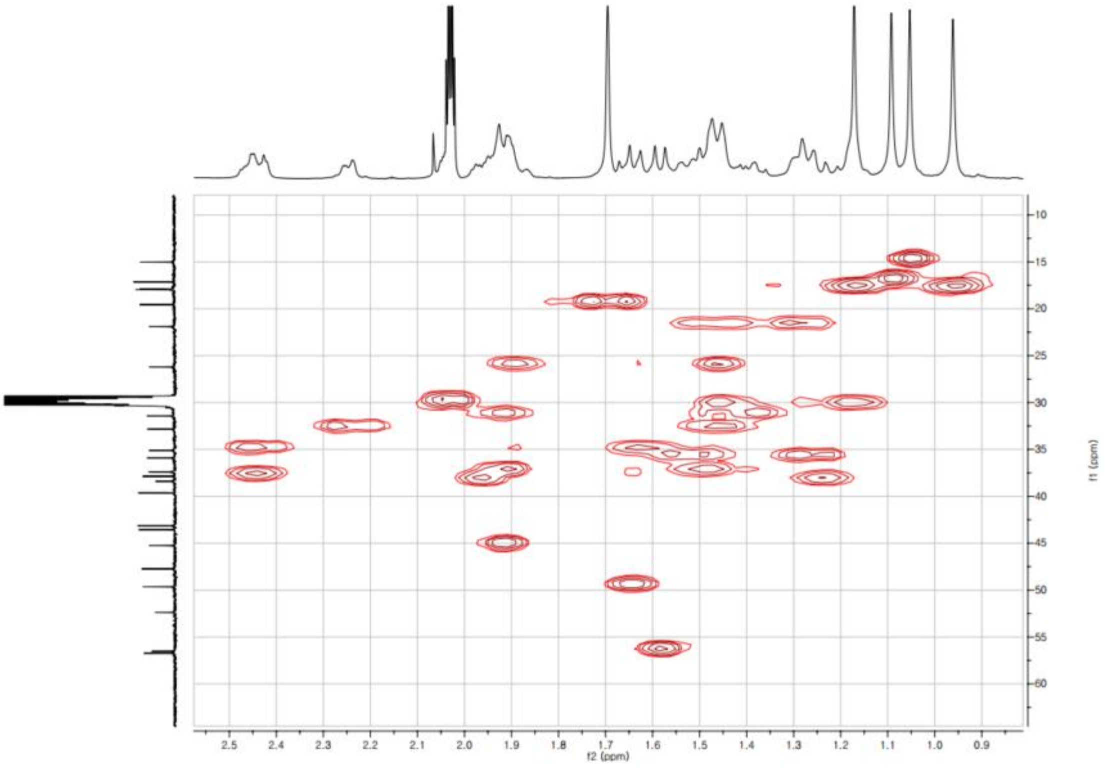

Figure S69. Expand HSQC spectrum of 3α,11α-dihydroxy-lup-20(29)-en-23,28-dioic acid in acetone-d_6

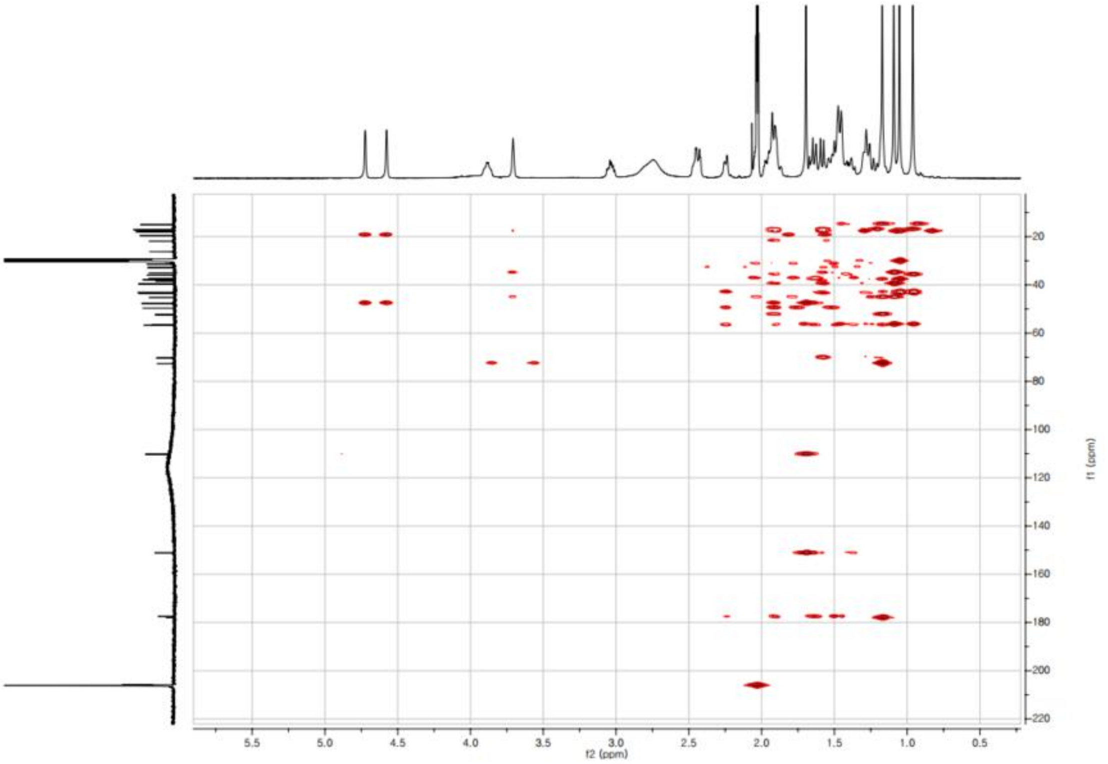

Figure S70. HMBC spectrum of 3α,11α-dihydroxy-lup-20(29)-en-23,28-dioic acid in acetone-d_6

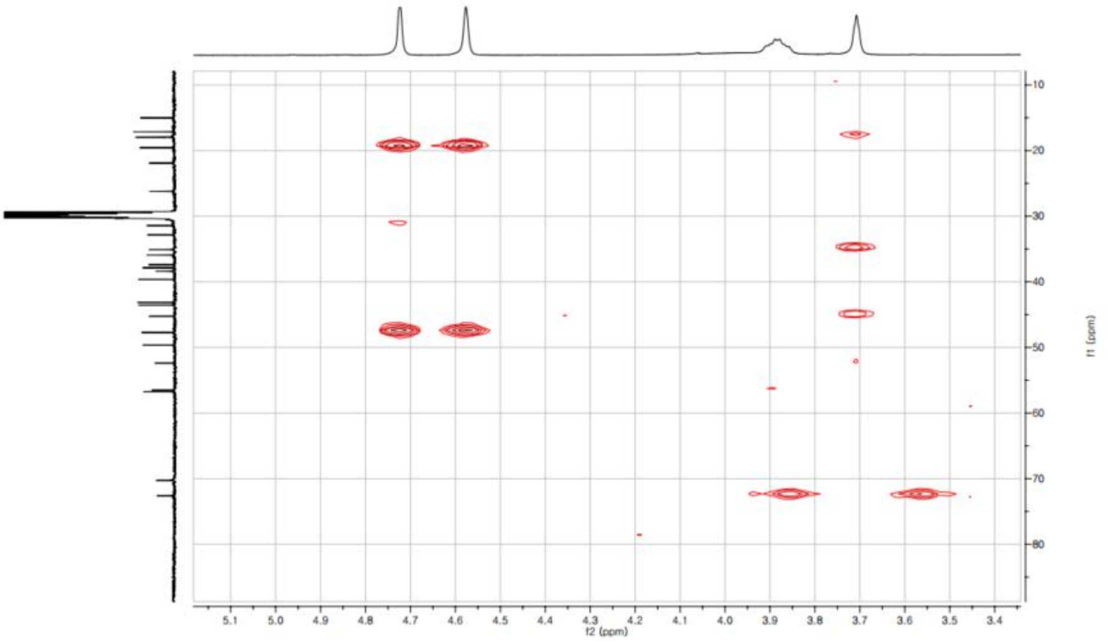

Figure S71. Expand HMBC spectrum of 3α,11α-dihydroxy-lup-20(29)-en-23,28-dioic acid in acetone-d_6

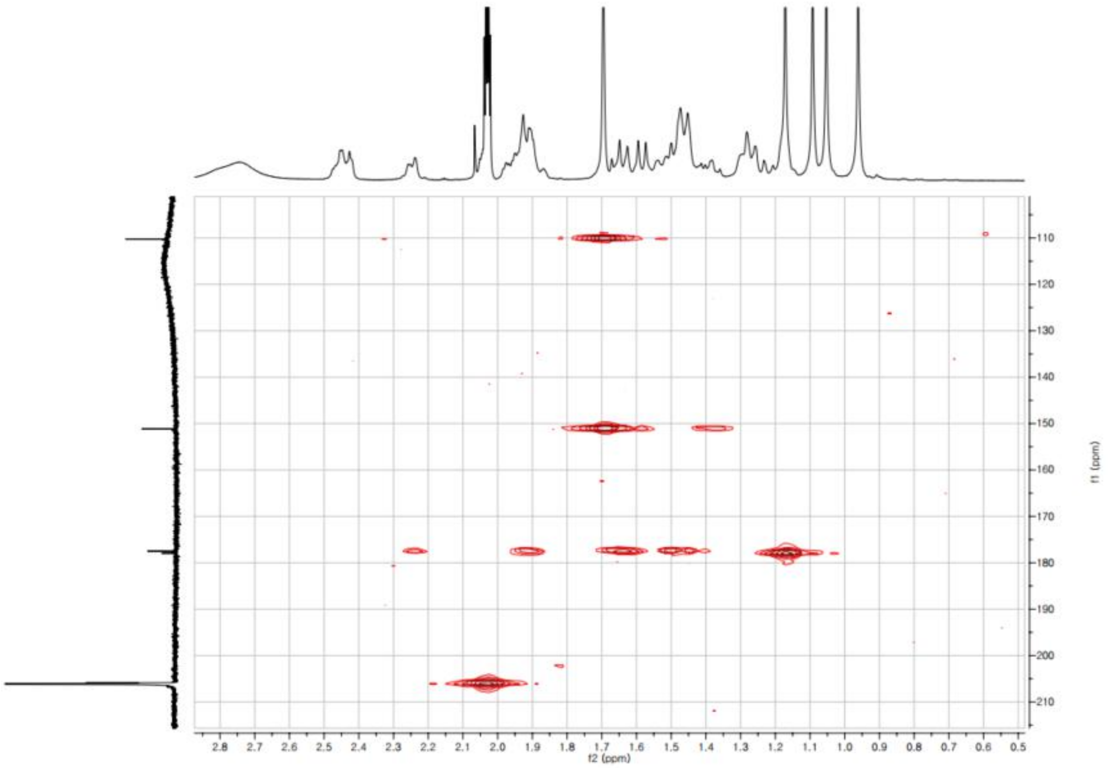

Figure S72. Expand HMBC spectrum of 3α,11α-dihydroxy-lup-20(29)-en-23,28-dioic acid in acetone-d_6

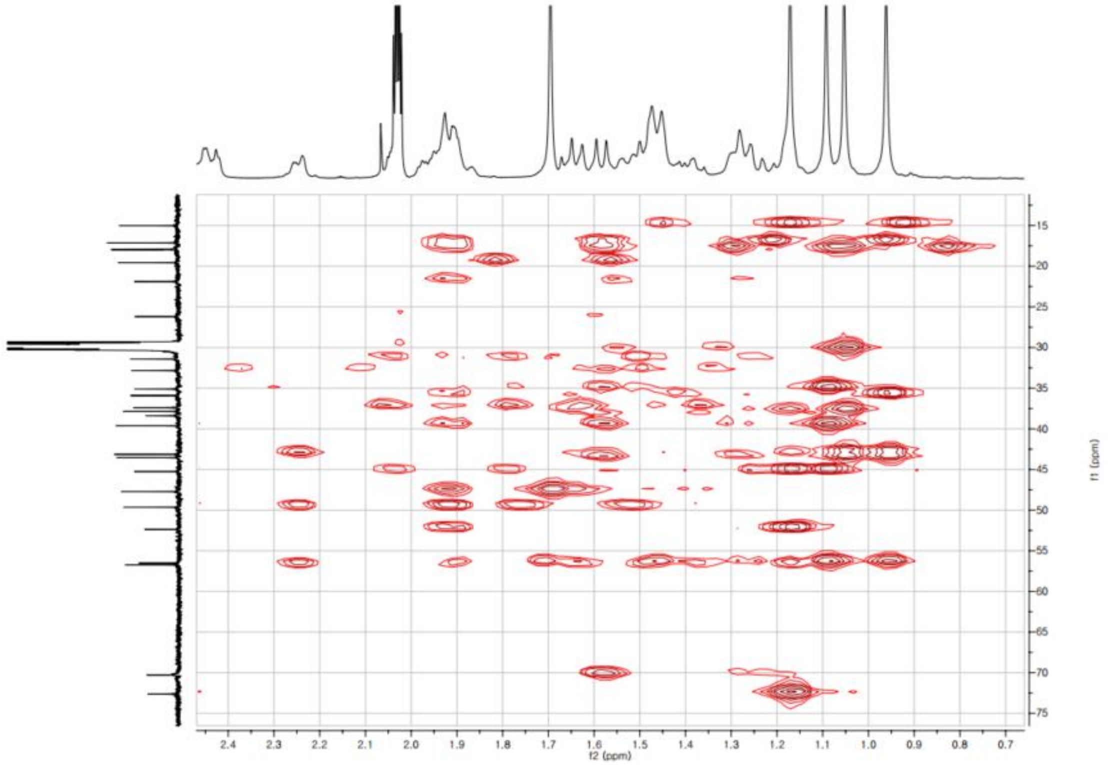

Figure S73. Expand HMBC spectrum of 3α,11α-dihydroxy-lup-20(29)-en-23,28-dioic acid in acetone-d_6

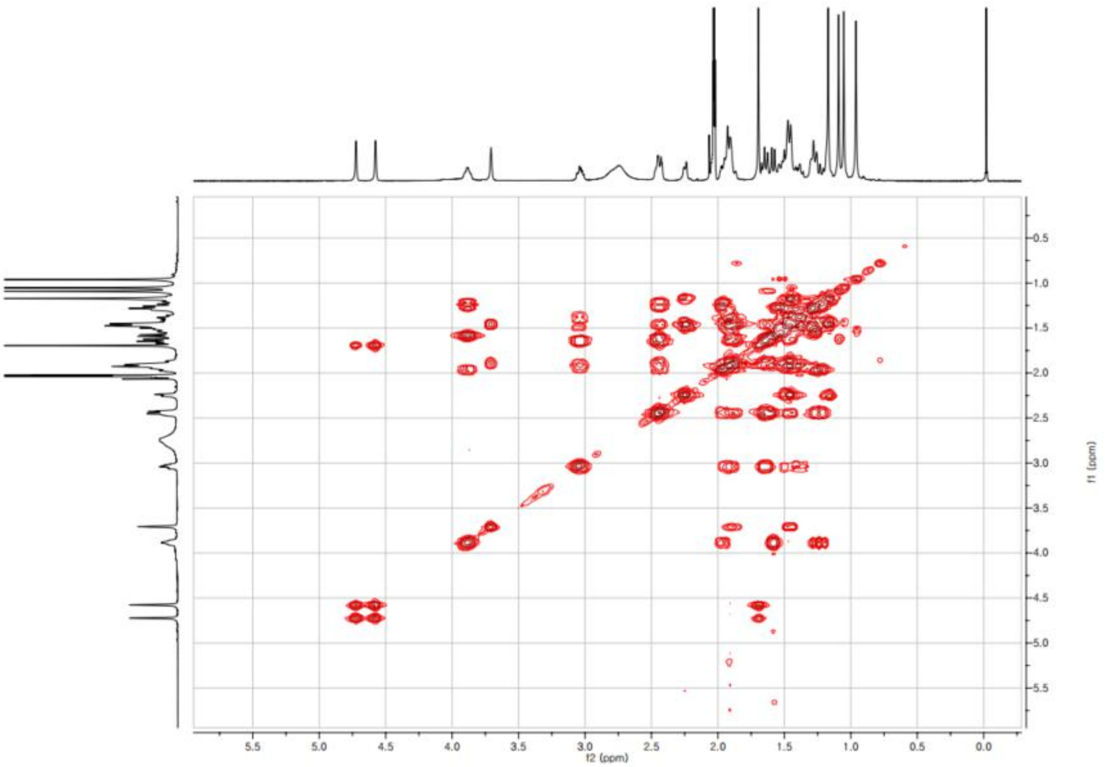

Figure S74. ^{1}H-^{1}H COSY spectrum of 3α,11α-dihydroxy-lup-20(29)-en-23,28-dioic acid in acetone-d_6

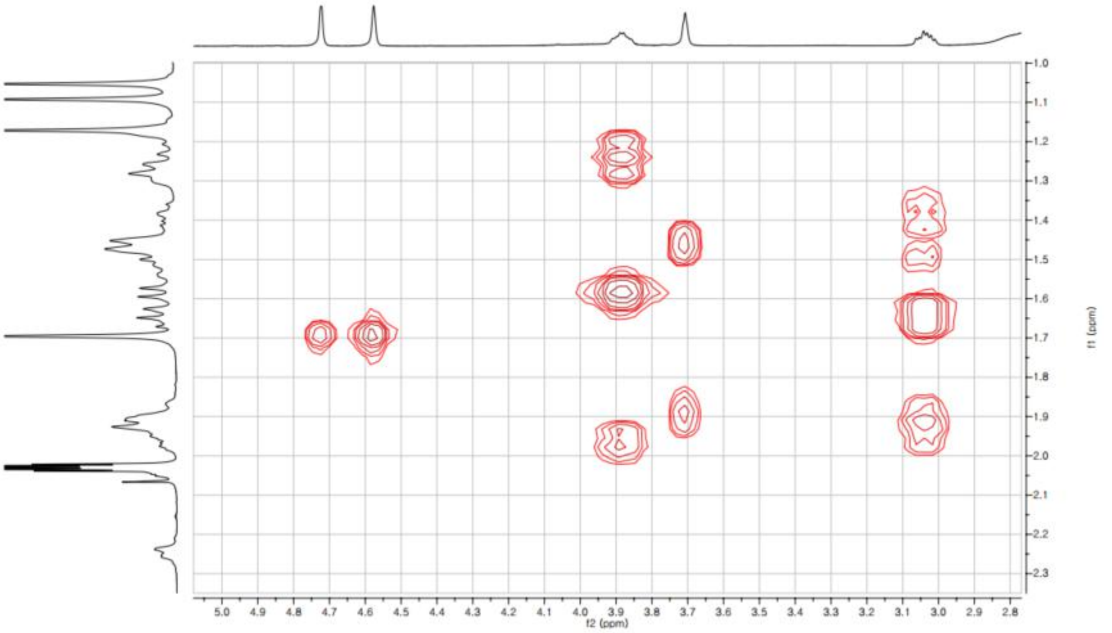

Figure S75. Expand ^{1}H-^{1}H COSY spectrum of 3α,11α-dihydroxy-lup-20(29)-en-23,28-dioic acid in acetone-d_6

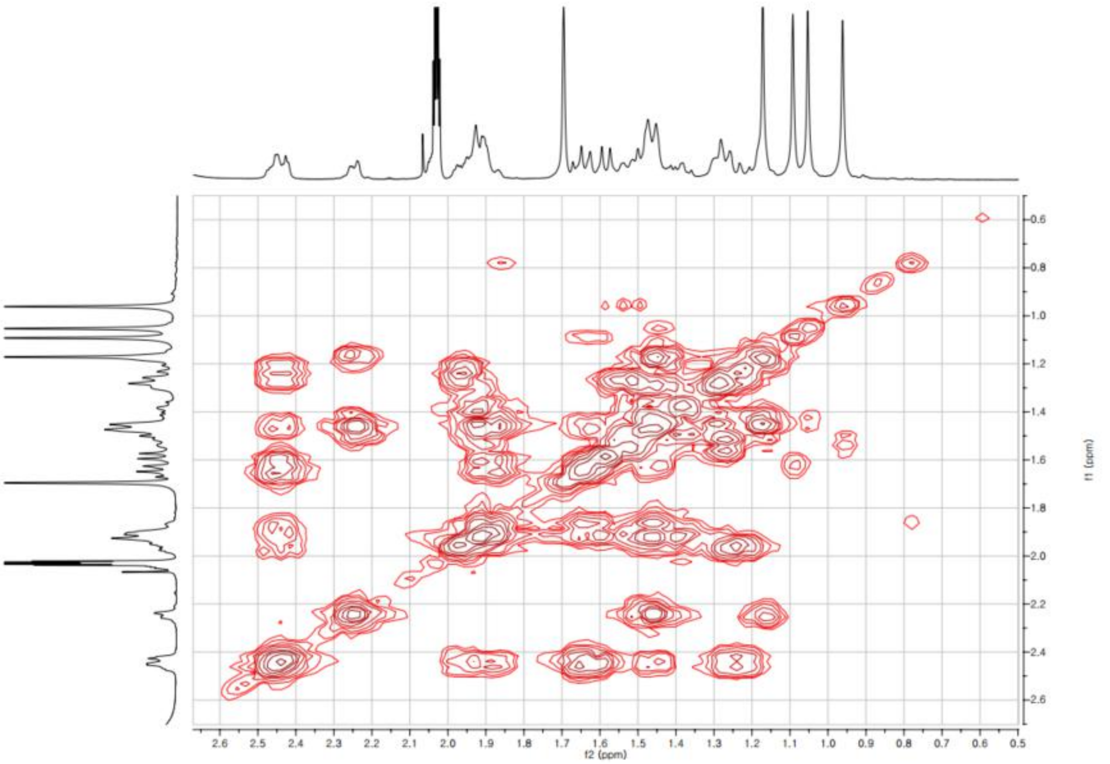

Figure S76. Expand ^{1}H-^{1}H COSY spectrum of 3α,11α-dihydroxy-lup-20(29)-en-23,28-dioic acid in acetone-d_6

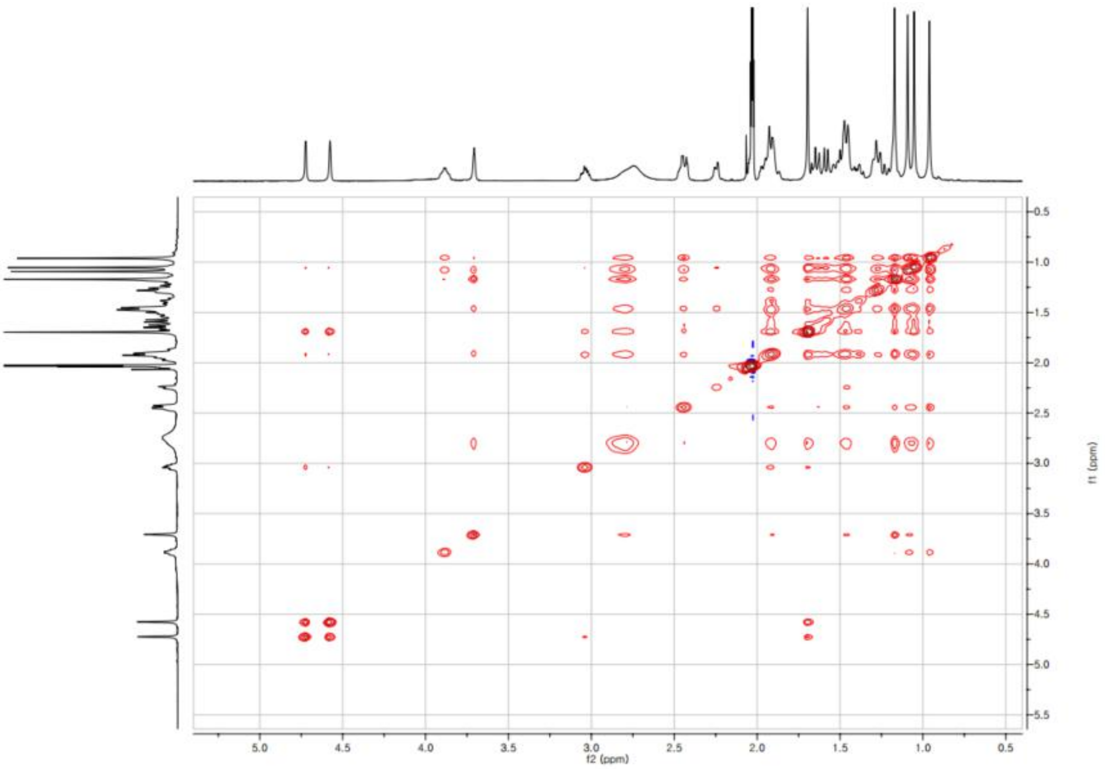

Figure S77. NOESY spectrum of 3α,11α-dihydroxy-lup-20(29)-en-23,28-dioic acid in acetone-d_6

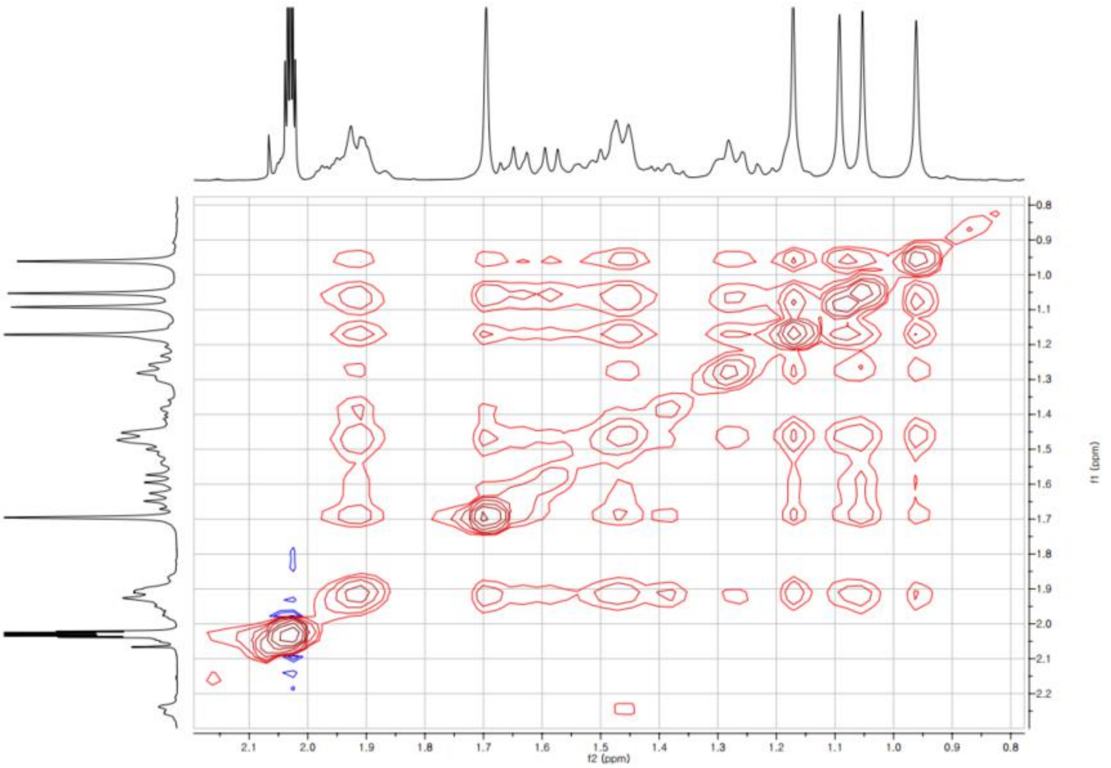

Figure S78. Expand NOESY spectrum of 3α,11α-dihydroxy-lup-20(29)-en-23,28-dioic acid in acetone-d_6

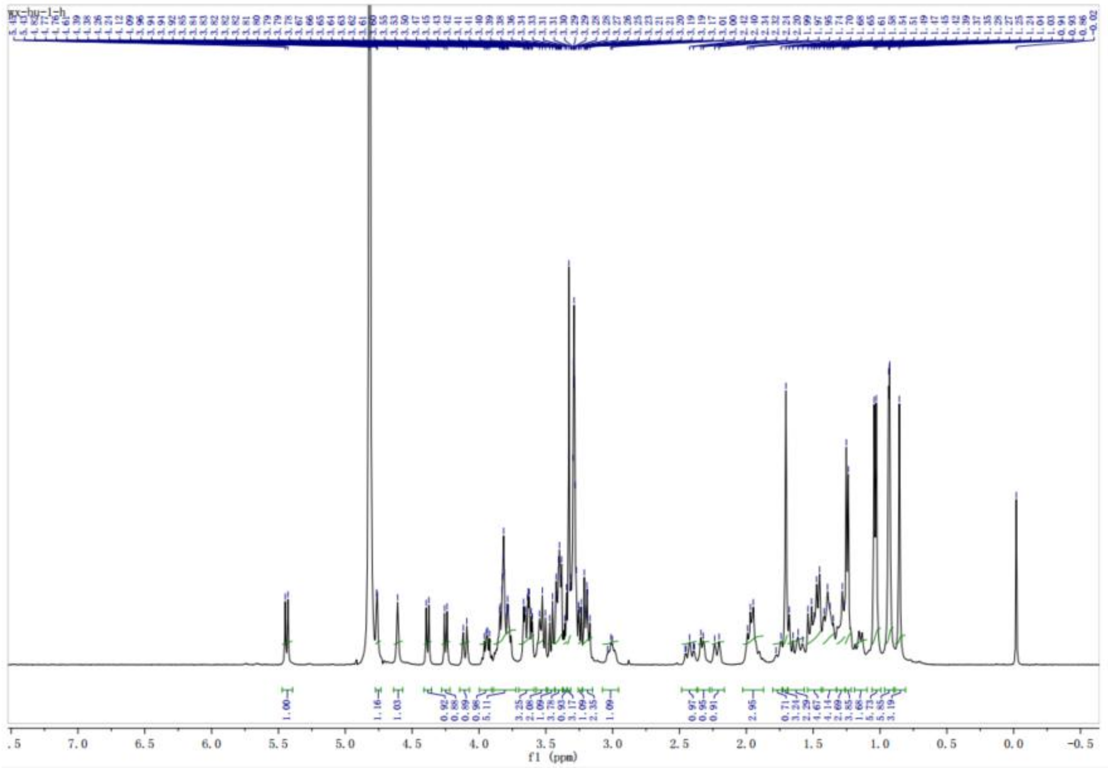

Figure S79. ^{1}H NMR spectrum of acankoreoside C in methanol-d_4

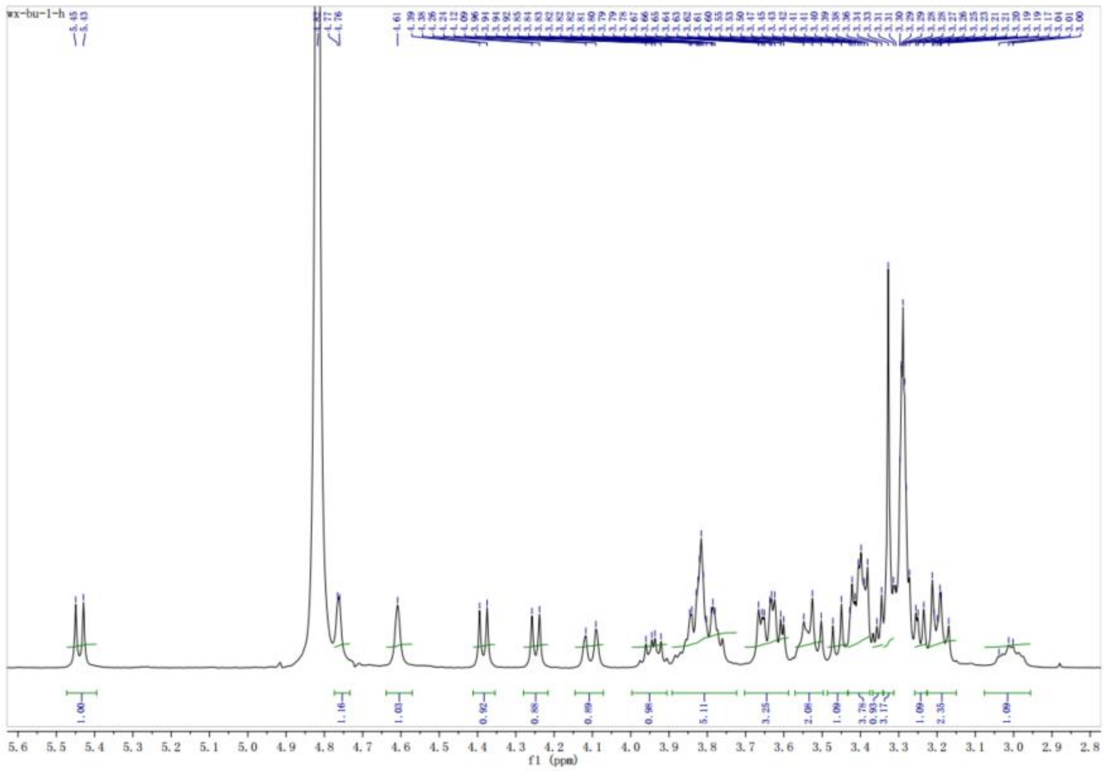

Figure S80. Expand ^{1}H NMR spectrum of acankoreoside C in methanol-d_4

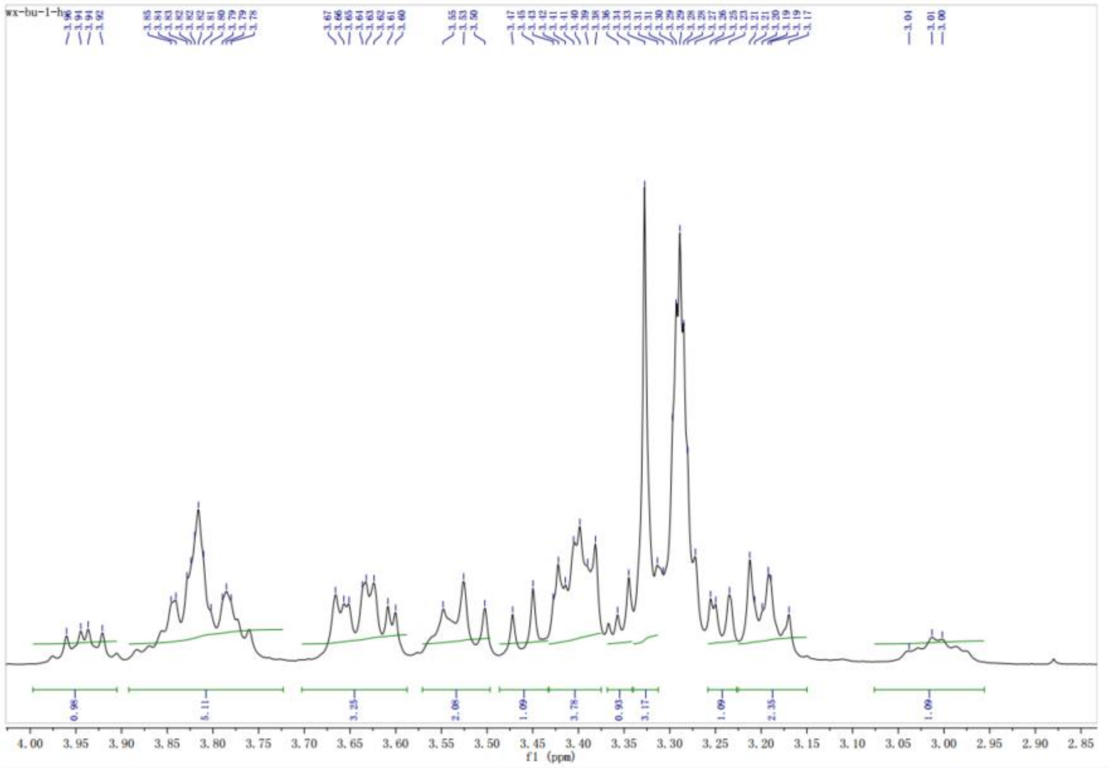

Figure S81. Expand ^{1}H NMR spectrum of acankoreoside C in methanol-d_4

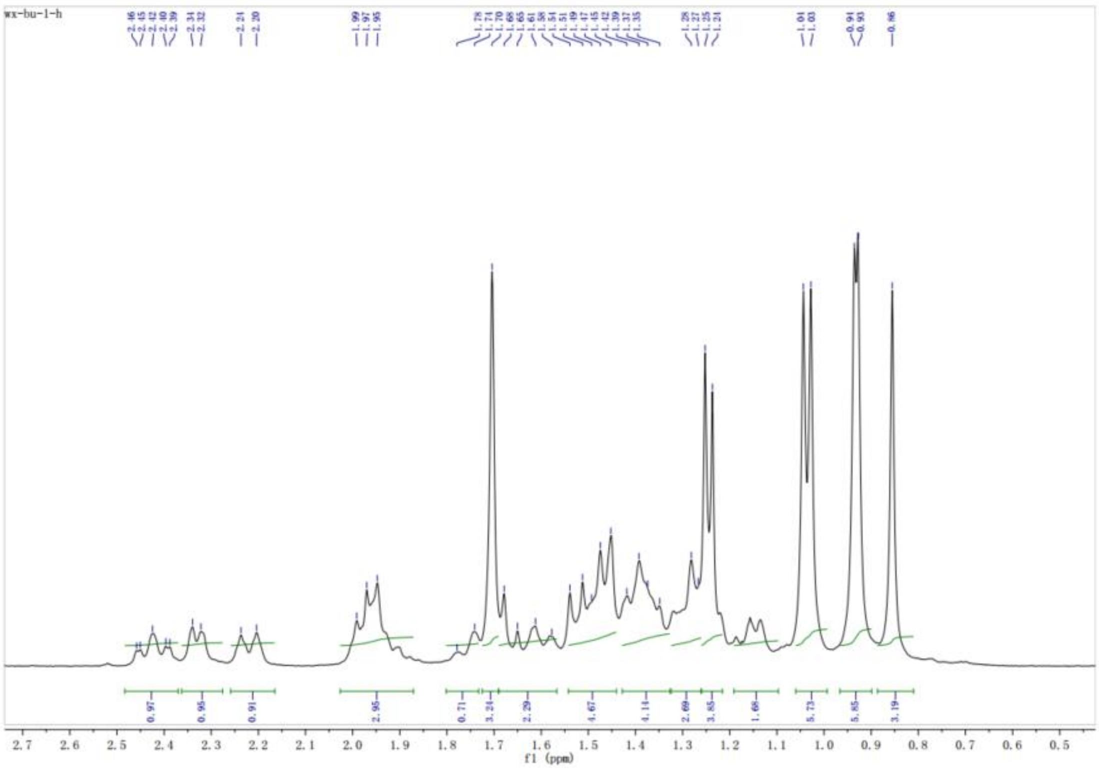

Figure S82. Expand ^{1}H NMR spectrum of acankoreoside C in methanol-d_4

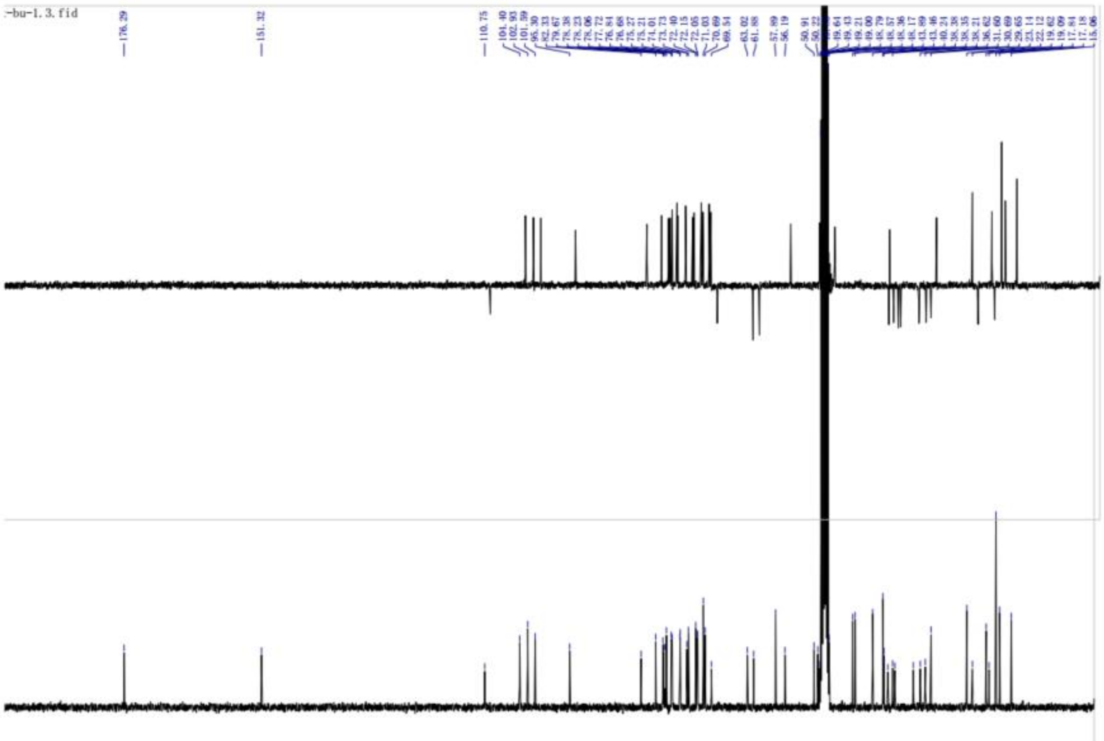

Figure S83. ^{13}C NMR spectrum of acankoreoside C in methanol-d_4

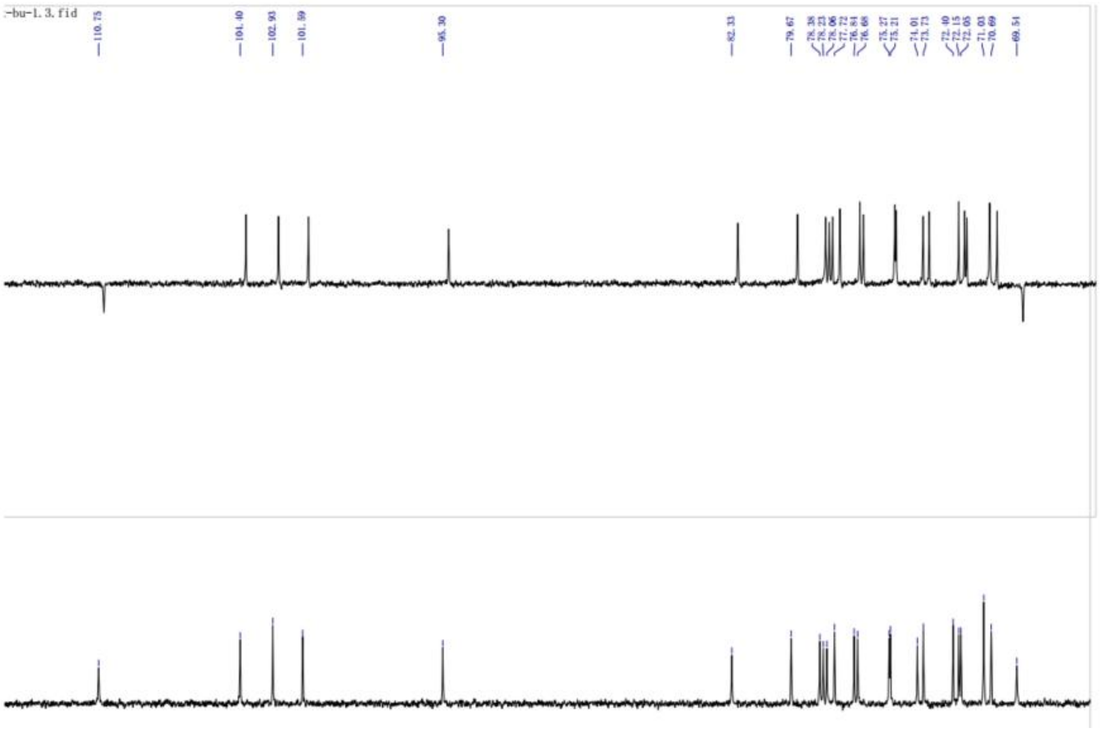

Figure S84. Expand ^{13}C NMR spectrum of acankoreoside C in methanol-d_4

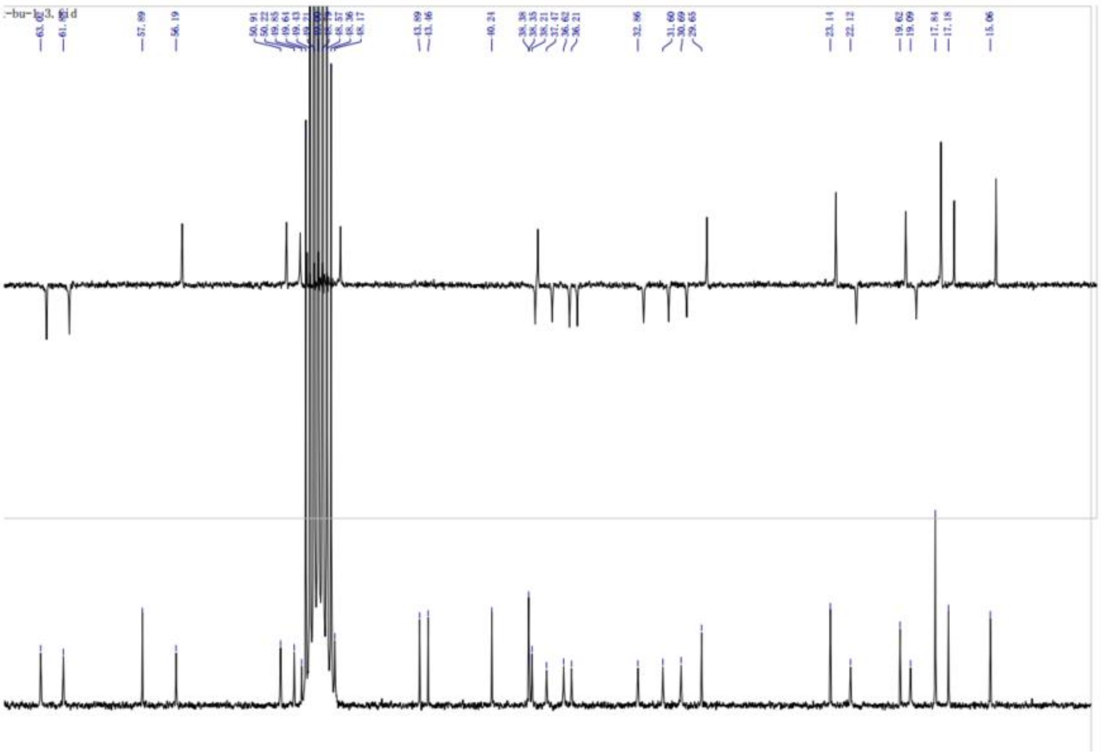

Figure S85. Expand ^{13}C NMR spectrum of acankoreoside C in methanol-d_4

Table S1. ^{1}H NMR (400 MHz) and ^{13}C NMR (100 MHz) spectral data of compound **1** (acetone-d_6, δ ppm)

Position	$δ_C$	DEPT	$δ_H$ [mult. (*J* in Hz)]	HMBC correlations
C-1	75.46	CH	3.84 (1H, dd, 8.16, 3.96)	H-2, H-25
C-2	37.36	CH_2	1.82 (1H, m);1.87 (1H, m)	H-1
C-3	73.99	CH	3.82 (1H, t, 2.40)	H-2, H-24
C-4	52.27	C	-	H-2, H-5, H-24
C-5	45.25	CH	1.95 (1H, m)	H-3, H-6, H-7, H-24, H-25
C-6	22.09	CH_2	1.35 (1H, m);1.57 (1H, m)	H-5
C-7	35.19	CH_2	1.31 (1H, m);1.56 (1H, m)	H-5, H-26
C-8	42.76	C	-	H-6, H-9, H-11, H-26, H-27
C-9	52.98	CH	1.72 (1H, dd, 10.08, 2.68)	H-1, H-11, H-25, H-26
C-10	44.33	C	-	H-1, H-5, H-11, H-25
C-11	24.68	CH_2	1.32 (1H, m); 2.43 (1H, brd, 9.80)	-
C-12	26.87	CH_2	1.12 (1H, dd, 10.52, 3.76); 1.68 (1H, m)	-
C-13	39.14	CH	2.34 (1H, td, 10.32, 3.0)	H-12, H-18, H-27
C-14	43.72	C	-	H-13, H-16, H-26, H-27
C-15	30.75	CH_2	1.20 (1H, m); 1.46 (1H, m)	H-16, H-27
C-16	33.15	CH_2	1.48 (1H, m); 2.25 (1H, dt, 9.84, 2.8)	H-18
C-17	56.98	C	-	H-13, H-15, H-16, H-18, H-22
C-18	50.30	CH	1.64 (1H, m)	H-12, H-13, H-16, H-19, H-22
C-19	48.24	CH	3.05 (1H, td, 8.76, 4.0)	H-18, H-21, H-22, H-29, H-30
C-20	151.89	C	-	H-18, H-19, H-21, H-29, H-30
C-21	31.61	CH_2	1.34 (1H, m); 1.90 (1H, m)	H-19, H-22
C-22	37.84	CH_2	1.48 (1H, m); 1.92 (1H, m)	H-18, H-21
C-23	178.16	COOH	-	H-3, H-5, H-24
C-24	17.92	CH_3	1.17 (3H, s)	H-5
C-25	13.24	CH_3	0.97 (3H, s)	H-1, H-5, H-9
C-26	17.31	CH_3	0.99 (3H, s)	H-7, H-9
C-27	15.40	CH_3	1.07 (3H, s)	H-13, H-15
C-28	177.84	COOH	-	H-16, H-18, H-22
C-29	110.26	CH_2	4.58 (1H, m); 4.72 (1H, d, 1.68)	H-19, H-30
C-30	19.72	CH_3	1.71 (3H, s)	H-19, H-29

Table S2. ^{1}H NMR (400 MHz) and ^{13}C NMR (100 MHz) spectral data of compound **2** (methanol-d_4, δ ppm)

Position	$δ_C$	DEPT	$δ_H$ [mult. (*J* in Hz)]	HMBC correlations
Aglycone				
C-1	76.33	CH	3.80 (1H, m)	H-2, H-3, H-5, H-25
C-2	36.79	CH_2	1.72 (1H, m);1.80 (1H, m)	H-1
C-3	74.14	CH	3.67 (1H, m)	H-1, H-2, H-24
C-4	52.24	C	-	H-2, H-5, H-24
C-5	46.11	CH	1.87 (1H, m)	H-3, H-6, H-7, H-9, H-24, H-25
C-6	22.17	CH_2	1.26 (1H, m); 1.58 (1H, m)	H-5, H-7
C-7	35.09	CH_2	1.30 (1H, m); 1.55 (1H, m)	H-5, H-6, H-26
C-8	42.96	C	-	H-6, H-7, H-9, H-26, H-27
C-9	53.06	CH	1.72 (1H, m)	H-1/H-5/H-7/H-11/H-12/H-25/H-26
C-10	44.47	C	-	H-1, H-5, H-6, H-9, H-11, H-25
C-11	24.82	CH_2	1.36 (1H, m); 2.28 (1H, m)	H-12
C-12	26.94	CH_2	1.12 (1H, m); 1.68 (1H, m)	H-13
C-13	39.13	CH	2.27 (1H, m)	H-12, H-18, H-19, H-27
C-14	43.82	C	-	H-13, H-16, H-18, H-26, H-27
C-15	30.86	CH_2	1.15 (1H, m); 1.54 (1H, m)	H-13, H-16, H-27
C-16	32.95	CH_2	1.44 (1H, m); 2.33 (1H, m)	H-15, H-18, H-22
C-17	57.93	C	-	H-15, H-16, H-18, H-21, H-22

C-18	50.6	CH	1.65 (1H, m)	H-13, H-16, H-19, H-21, H-22
C-19	48.36	CH	3.0 (1H, m)	H-18, H-21, H-29, H-30
C-20	151.77	C	-	H-18, H-19, H-21, H-29, H-30
C-21	31.55	CH_2	1.37 (1H, m);1.94 (1H, m)	H-19, H-22
C-22	37.68	CH_2	1.48 (1H, m);1.94 (1H, m)	H-21
C-23	182.6	COOH	-	H-5, H-24
C-24	18.08	CH_3	1.09 (3H, s)	H-5
C-25	13.17	CH_3	0.95 (3H, s)	H-1, H-5, H-9
C-26	17.14	CH_3	0.98 (3H, s)	H-7, H-9
C-27	15.1	CH_3	1.03 (3H, s)	H-13, H-15
C-28	176.4	CO-S	-	H-16, H-18, H-22, glc-H-1
C-29	110.41	CH_2	4.58 (1H, brs); 4.72 (1H, brs)	H-30
C-30	19.49	CH_3	1.70 (3H, s)	H-19, H-29
C-28-O-inner glc				
1	95.26	CH	5.45 (1H, d, 6.56)	glcH-2
2	74.00	CH	3.33 (1H, m)	glcH-3
3	78.28	CH	3.42 (1H, m)	glcH-2, glcH-4, glcH-5
4	70.95	CH	3.43 (1H, m)	glcH-2, glcH-3, glcH-5, glcH-6
5	78.06	CH	3.54 (1H, m)	glcH-1, glcH-4, glcH-6
6	69.55	CH_2	3.81 (1H, m);<br>4.11 (1H, dd, 9.48, 1.36)	glcH-1′
glc′(1→6)glc				
1′	104.56	CH	4.37 (1H, d, 6.28)	glcH-6, glcH-2′
2′	75.32	CH	3.23 (1H, m)	glcH-3′, glcH-4′
3′	76.71	CH	3.45 (1H, m)	glcH-2′, glcH-5′
4′	79.51	CH	3.53 (1H, m)	glcH-3′, glcH-6′, rha H-1″
5′	76.89	CH	3.30 (1H, m)	glcH-4′, glcH-6′
6′	61.90	CH_2	3.65 (1H, m); 3.80 (1H, m)	glcH-4′
rha(1→4)glc′				
1″	102.92	CH	4.84 (1H, overlapped)	glcH-4′
2″	72.44	CH	3.81 (1H, m)	rhaH-1″, rhaH-3″, rhaH-4″
3″	72.16	CH	3.62 (1H, m)	rhaH-1″, rhaH-2″, rhaH-4″
4″	73.75	CH	3.38 (1H, m)	rhaH-2″, rhaH-3″, rhaH-5″, rhaH-6″
5″	70.64	CH	3.95 (1H, m)	rhaH-1″, rhaH-4″, rhaH-6″
6″	17.84	CH_3	1.25 (3H, d, 4.96)	rhaH-4″

Table S3. NMR spectral data of compounds **3-4**.

**Position**	**3**<br>δ_C[a,c]	δ_H[a,d] **[mult. (*J* in Hz)]**	**4**<br>δ_C[b,c]	δ_H[b,d] **[mult. (*J* in Hz)]**
Aglycone				
C-1	32.84	1.46 (1H, m); 2.24 (1H, m)	36.62	-
C-2	26.21	1.46 (1H, m); 1.90 (1H, m)	22.12	-
C-3	72.65	3.71 (1H, brs)	82.33	3.67 (1H, m)
C-4	52.39	-	38.35	-
C-5	45.26	1.92 (1H, m)	50.91	-
C-6	21.91	1.30 (1H, m); 1.48 (1H, m)	19.09	-
C-7	35.92	1.29 (1H, m); 1.55 (1H, m)	36.21	-
C-8	43.15	-	43.46	-
C-9	56.52	1.59 (1H, m)	56.19	1.72 (1H, m)
C-10	39.64	-	40.24	-
C-11	70.28	3.90 (1H, m)	70.69	3.85 (1H, m)
C-12	38.41	1.24 (1H, m); 1.96 (1H, m)	38.38	-
C-13	37.87	2.44 (1H, m)	38.21	2.79 (1H, m)
C-14	43.56	-	43.89	-
C-15	30.26	1.18 (1H, m); 1.46 (1H, m)	30.69	-

C-16	35.11	1.63 (1H, m); 2.45 (1H, m)	32.86	-
C-17	56.74	-	57.89	-
C-18	49.65	1.64 (1H, m)	50.22	1.65 (1H, m)
C-19	47.74	3.05 (1H, m)	48.17	3.01 (1H, m)
C-20	151.14	-	151.32	-
C-21	31.44	1.38 (1H, m); 1.91 (1H, m)	31.60	-
C-22	37.40	1.48 (1H, m); 1.92 (1H, m)	37.47	-
C-23	177.78	-	29.65	1.04 (3H, s)
C-24	17.92	1.17 (3H, s)	23.14	0.93 (3H, s)
C-25	17.97	0.96 (3H, s)	17.18	1.03 (3H, s)
C-26	17.14	1.09 (3H, s)	17.84	0.94 (3H, s)
C-27	15.02	1.05 (3H, s)	15.06	0.86 (3H, s)
C-28	177.51	-	176.29	-
C-29	110.26	4.58 (1H, brs); 4.72 (1H, brs)	110.75	4.61 (1H, brs); 4.76 (1H, brs)
C-30	19.57	1.70 (3H, s)	19.62	1.70 (3H, s)
C-28-O-inner glc				
1			95.30	5.45 (1H, d, 6.56)
2			74.01	3.32 (1H, m)
3			78.23	3.40 (1H, m)
4			71.03	3.41 (1H, m)
5			77.72	3.53 (1H, m)
6			69.54	3.81 (1H, m);<br>4.12 (1H, dd, 9.48, 1.36)
glc′(1→6)glc				
1′			104.4	4.39 (1H, d, 6.28)
2′			75.21	3.23 (1H, m)
3′			76.68	3.43 (1H, m)
4′			79.67	3.52 (1H, m)
5′			76.84	3.28 (1H, m)
6′			61.8	3.65 (1H, m); 3.80 (1H, m)
rha(1→4)glc′				
1′′			102.93	4.84 (1H, overlapped)
2′′			72.40	3.81 (1H, m)
3′′			72.15	3.62 (1H, m)
4′′			73.73	3.38 (1H, m)
5′′			70.69	3.95 (1H, m)
6′′			17.84	1.25 (3H, d, 4.96)
C-3-O-glc′′				
1′′′			101.59	4.26 (1H, d, 6.56)
2′′′			75.27	3.32 (1H, m)
3′′′			78.38	3.52 (1H, m)
4′′′			72.05	3.84 (1H, m)
5′′′			78.06	3.53 (1H, m)
6′′′			63.02	3.62 (1H, m); 3.82 (1H, m)

Note: Assignments were done by HMQC, HMBC, ^{1}H-^{1}H COSY, and NOESY experiments; Glc: D-glucopyranosyl; Rha: L-rhamnopyranosyl; [a] Measured in CD_3COCD_3; [b] Measured in CD_3OD; [c] 100 MHz; [d] 400 MHz
